# Supplementary material for: The Global Impact of Alcohol Consumption on Premature Mortality and Health in 2016
Source: Nutrients. 2021 Sep 9;13(9):3145. doi: 10.3390/nu13093145 (PMC8470338; doi:10.3390/nu13093145)
Supplement: Supplementary file 1 [file nutrients-13-03145-s001.zip › nutrients-1345474-supplementary.pdf]

APPENDIX

The Global Impact of Alcohol Consumption on Premature Mortality and Health in 2016

Table of contents

Supplemental Methods **4**

Statistical analysis code and input files 4

Estimating the number of deaths among drivers resulting from motor vehicle accidents 4

Modelling alcohol consumption among current drinkers 4

Modelling population attributable fractions 5

Estimating the population-attributable fractions for ischaemic stroke, ischaemic heart disease and injuries 5

Estimating the population-attributable fraction for road injuries sustained by people other than the driver 6

Supplemental Results **10**

Table A1. Alcohol related diseases conditions and injury and the methods used to estimate the alcohol-attributable burden 7

Table A2. Global alcohol-attributable deaths by age and cause in 2016 among men and 95% uncertainty intervals 13

Table A3. Global alcohol-attributable deaths by age and cause in 2016 among women and 95% uncertainty intervals 17

Table A4. Global alcohol-attributable deaths by age and cause in 2016 and 95% uncertainty intervals 21

Table A5. Global alcohol-attributable premature deaths and cause in 2016 by sex and 95% uncertainty intervals 25

Table A6. Global alcohol-attributable years of life lost (YLL) by age and cause in 2016 among men and 95% uncertainty intervals 26

Table A7. Global alcohol-attributable years of life lost (YLL) by age and cause in 2016 among women and 95% uncertainty intervals 29

Table A8. Global alcohol-attributable years of life lost (YLL) by age and cause in 2016 and 95% uncertainty intervals 32

Table A9. Global alcohol-attributable years of life lost (YLL) by cause and sex in 2016 and 95% uncertainty intervals 35

Table A10. Global alcohol-attributable years lived with disability (YLD) by age and cause in 2016 among men and 95% uncertainty intervals 36

Table A11. Global alcohol-attributable years lived with disability (YLD) by age and cause in 2016 among women and 95% uncertainty intervals 39

Table A12. Global alcohol-attributable years lived with disability (YLD) by age and cause in 2016 and 95% uncertainty intervals 42

Table A13. Global alcohol-attributable premature years lived with disability (YLD) by cause and sex in 2016 and 95% uncertainty intervals 45
Table A14. Global alcohol-attributable disability adjusted life years (DALYs) lost by age and cause in 2016 among men and 95% uncertainty intervals....46
Table A15. Global alcohol-attributable disability adjusted life years (DALYs) lost by age and cause in 2016 among women and 95% uncertainty intervals....49
Table A16. Global alcohol-attributable disability adjusted life years (DALYs) lost by age and cause in 2016 and 95% uncertainty intervals....52
Table A17. Global alcohol-attributable premature disability adjusted life years (DALYs) lost by cause and sex in 2016 and 95% uncertainty intervals 55

Figure A1. Map of Human Development Index scores....9
Figure A2. Alcohol-attributable years of life lost (YLL) and years lived with disability (YLD) globally in 2016 among people 0 to 69 years of age 10
Figure A3. Alcohol-attributable years of life lost (YLL) and years lived with disability (YLD) among people 0 to 70 years of age by global burden of disease region....11
Figure A4. Alcohol-attributable years of life lost (YLL) and years lived with disability (YLD) among people 0 to 70 years of age by Human Development Index region 12

Supplemental Methods

Statistical analysis code and input files

All statistical code (i.e., R code) and input files used to produce the results presented in this paper are available to the general public. To obtain the code and input files, please contact the corresponding author, Kevin Shield Ph.D. (Kevin.Shield@camh.ca).

The methods summarized in this section are based on the article [1].

Estimating the number of deaths among drivers resulting from motor vehicle accidents

Formula A1 was used to estimate road injury deaths involving the driver and those involving others. This formula estimates the number of deaths (D) among drivers (d) based on the fraction (F) of injury events occurring among drivers by sex (indexed by i) and age (indexed by p). Fractions of injury events which occurred to the driver and people other than the driver were obtained from the World Health Organization (WHO) road traffic deaths database [2]. For countries where data were not available, fractions of injuries were imputed based on global burden of disease regional averages. Formula A1 assumes that all road injuries to drivers occurred among people aged 15 years or older. Th road injury years of life lost (YLL), years lived with disability (YLD), and disability adjusted life years (DALYs) lost among drivers, were based on the fraction of deaths among drivers compared to all road deaths (by age and sex).

Formula A1

$$Dd_{p,i} = \frac{F_i \cdot F_p \cdot \sum_{p=1}^{pn} \sum_{i=1}^{in} D_{p,i}}{D_{p,i}}$$

Modelling alcohol consumption among current drinkers

Alcohol use (measured in grams per day) was modelled based on (i) the prevalence of current drinkers (i.e., past year drinkers), former drinkers, and lifetime abstainers (ii) the average volume of alcohol consumption among current drinkers, and (iii) the prevalence of heavy episodic drinking (HED; defined as drinking 60 grams or more of pure alcohol on one occasion) among current drinkers. Data on alcohol consumption (drinking status and adult per capita consumption (APC)) and HED were obtained from the study by Manthey *et al.*, [3]. Data on drinking status and HED were obtained by year, age group (15–19, 20–24, 25–34, 35–49, 50–64, and 65 years of age and older), and sex.

Alcohol consumed by current drinkers was adjusted using a correction factor of 0.8 to account for (i) alcohol that was not consumed, and (ii) the underreporting of alcohol use in medical observation studies from which the relative risk (RR) estimates used in this study were obtained [4].

Average daily alcohol consumption among current drinkers was modelled using a Gamma distribution [9][10]. This method models standard deviation (σ) of the Gamma distribution of alcohol consumption based on mean (μ) consumption of alcohol by sex (see Formula A2) [9,10]. The coefficient of sex is 1 for women and 0 for men in Formula A2.

Formula A2

$$\hat{\sigma}_{shifted=}(1 \cdot 171 + 0.087 * sex) * \hat{\mu}_{shifted}$$

Modelling population attributable fractions

The Population Attributable Fraction (PAF) for alcohol related conditions (except for ischaemic diseases, injuries, 100% attributable to alcohol and injuries) were estimated using formula A3. Formula A3 is based on the prevalence of lifetime abstainers (P_A), former drinkers (P_{FD}) and current drinkers (P_{CD}) and corresponding relative risk (RR) estimates.

Formula A3

$$PAF = \frac{P_A + P_{FD}RR_{FD} + \int_{>0}^{150} P_{CD}(x)RR_{CD}(x)dx - 1}{P_A + P_{FD}RR_{FD} + \int_{>0}^{150} P_{CD}(x)RR_{CD}(x)dx}$$

Estimating the population-attributable fractions for ischaemic stroke, ischaemic heart disease and injuries

The PAF for ischaemic stroke and ischaemic heart disease were estimated based on Formulas A4 and A5. These formulae are based on the P_{FD} and P_{CD} combined with the corresponding RRs. Formula A5 accounts for the patterns of alcohol consumption (i.e., the prevalences of current drinkers who HEDs (P_{HED}) and are not HED (P_{NHED})).

Formula A4

$$PAF = \frac{P_{FD}(RR_{FD} - 1) + P_{CD}(RR_{CD} - 1)}{P_{FD}(RR_{FD} - 1) + P_{CD}(RR_{CD} - 1) + 1}$$

Formula A5

$$P_{CD}(RR_{CD} - 1) = \int_{>0}^{60} P_{NHED}(x)RR_{NHED}(x)dx + \int_{>0}^{60} P_{HED}(x)RR_{HED}(x)dx + \int_{60}^{150} P_{HED}(x)RR_{HED}(x)dx - P_{CD}$$

Estimating the population-attributable fraction for road injuries sustained by people other than the driver

The PAFs for road injuries to people other than the driver (nd) were estimated in accordance with Formula A6. This formula is based on the country-, sex- (indexed by p), and age- (indexed by i) specific deaths (D) and PAFs for road injuries affecting the driver. This method assumes that number of road injuries to non-drivers involving an intoxicated driver are equal to injuries involving a non-intoxicated driver. This method does not account for non-intoxicated drivers killed or injured by intoxicated drivers.

Formula A6

$$PAF_{nd} = \frac{\sum_{p=1}^{pn} \sum_{i=1}^{in} D_{p,i} \cdot PAF_{p,i}}{\sum_{p=1}^{pn} \sum_{i=1}^{in} D_{p,i}}$$

Table A1. Alcohol related diseases conditions and injury and the methods used to estimate the alcohol-attributable burden

Cause 2015	GHE 2015 cause category				ICD-10 coding	Method for estimating the alcohol-attributable burden	RR source	Causality
10	I.	Communicable, maternal, perinatal and nutritional conditions			A00–B99, D50–53, D64.9, E00–02, E40–46, E50–64, G00–04, G14, H65–66, J00–22, N70–73, O00–99, P00–96, U04			
20		A.	Infectious and parasitic diseases		A00–B99, G00–04, G14, N70–73, P37.3, P37.4			
30			1	Tuberculosis	A15–19, B90	Levin's PAF method	[11]	[12]
100			3	HIV/AIDS	B20–24	Levin's PAF methodology	[13]	[13,14]
380		B.	Respiratory infections		H65–66, J00–22, P23, U04			
390			1	Lower respiratory infections	J09–22, P23, U04	Levin's PAF methodology	[15]	[15-17]
600	II.	Noncommunicable diseases			C00–97, D00–48, D55–64 (minus D64.9), D65–89, E03–07, E10–34, E65–88, F01–99, G06–98 (minus G14), H00–61, H68–93, I00–99, J30–98, K00–92, L00–98, M00–99, N00–64, N75–98, Q00–99, X41–42, X44, X45, R95			
610		A.	Malignant neoplasms		C00–97			
620			1	Mouth and oropharynx cancers	C00–14			
621				a. Lip and oral cavity	C00–08	Levin's PAF methodology	[18,19]	[20,21]
623				c. other pharyngeal cancers	C09–10, C12–14	Levin's PAF methodology	[18,19]	[20,21]
630			2	Oesophagus cancer	C15	Levin's PAF methodology	[18,19]	[20,21]
650			4	Colon and rectum cancers	C18–21	Levin's PAF methodology	[18,22]	[20,21]
660			5	Liver cancer	C22	Levin's PAF methodology	[23,24]	[20,21]
700			9	Breast cancer	C50	Levin's PAF methodology	[18]	[20,21]
753			19	Larynx cancer	C32	Levin's PAF methodology	[18,19]	[20,21]
800		C.	Diabetes mellitus		E10–14 (minus E10.2–10.29, E11.2–11.29, E12.2, E13.2–13.29, E14.2)	Levin's PAF methodology	[25,26]	[25,26]
820		E.	Mental and substance use disorders		F04–99, G72.1, Q86.0, X41–42, X44, X45			
860			4	Alcohol use disorders	F10, G72.1, Q86.0, X45	100% alcohol attributable (alcohol is a necessary cause)	-	-
940		F.	Neurological conditions		F01–03, G06–98 (minus G14, G72.1)			
970			3	Epilepsy	G40–41	Levin's PAF methodology	[27]	[28-30]
1100		H.	Cardiovascular diseases		I00–99			
1120			2	Hypertensive heart disease	I10–15	Levin's PAF methodology	[31]	[32,33]
1130			3	Ischaemic heart disease	I20–25	Levin PAF methodology	[34] based on [35,36]	[37-39]
1140			4	Stroke	I60–69			
1141				a. Ischaemic stroke	G45–46.8, I63–63.9, I65–66.9, I67.2–67.848, I69.3–69.4	Levin PAF methodology	[34] based on [40]	[39,41,42]
1142				b. Intracerebral haemorrhage	I60–62.9, I67.0–67.1, I69.0–69.298	Levin PAF methodology	[43]	[39,41,42]

Cause 2015	GHE 2015 cause category			ICD-10 coding	Method for estimating the alcohol-attributable burden	RR source	Causality
1150		5	Cardiomyopathy, myocarditis, endocarditis	I30–33, I38, I40, I42	Regression based estimates	-	-
1210	J.	Digestive diseases		K20–92			
1230		2	Cirrhosis of the liver	K70, K74	Levin PAF methodology	[31]	[44]
1248		8	Pancreatitis	K85–86	Levin PAF methodology	[44-48]	[49]
1510	III.	Injuries		V01–Y89 (minus X41–42, X44, X45)			
1520	A.	Unintentional injuries		V01–X40, X43, X46–59, Y40–86, Y88, Y89			
1530		1	Road injury	V01–04, V06, V09–80, V87, V89, V99*	Levin PAF methodology	[31]	[50]
1540		2	Poisonings	X40, X43, X46–48, X49	Levin PAF methodology	[31]	[50]
1550		3	Falls	W00–19	Levin PAF methodology	[31]	[50]
1560		4	Fire, heat and hot substances	X00–19	Levin PAF methodology	[31]	[50]
1570		5	Drowning	W65–74	Levin PAF methodology	[31]	[50]
1575		6	Exposure to mechanical forces	W20–38, W40–43, W45, W46, W49–52, W75, W76	Levin PAF methodology	[31]	[50]
1590		8	Other unintentional injuries	Rest of V, W39, W44, W53–64, W77–99, X20–29, X50–59, Y40–86, Y88, Y89	Levin PAF methodology	[31]	[50]
1600	B.	Intentional injuries		X60–Y09, Y35–36, Y870, Y871			
1610		1	Self-harm	X60–84, Y870	Levin PAF methodology	[31]	[50]
1620		2	Interpersonal violence	X85–Y09, Y871	Levin PAF methodology	[31]	[50]

* Three digit ICD-10 codes include: V01.1-9, V02.1-9, V03.1-9, V04.1-9, V06.1-9, V09.2, V09.3, V10.3-9, V11.3-9, V12.3-9, V13.3-9, V14.3-9, V15.4-9, V16.4-9, V17.4-9, V18.4-9, V19.4-9, V20.3-9, V21.3-9, V22.3-9, V23.3-9, V24.3-9, V25.3-9, V26.3-9, V27.3-9, V28.3-9, V29.4-9, V30.4-9, V31.4-9, V32.4-9, V33.4-9, V34.4-9, V35.4-9, V36.4-9, V37.4-9, V38.4-9, V39.4-9, V40.4-9, V41.4-9, V42.4- 9, V43.4-9, V44.4-9, V45.4-9, V46.4-9, V47.4-9, V48.4-9, V49.4-9, V50.4-9, V51.4-9, V52.4-9, V53.4-9, V54.4-9, V55.4-9, V56.4-9, V57.4-9, V58.4-9, V59.4-9, V60.4-9, V61.4-9, V62.4-9, V63.4-9, V64.4-9, V65.4-9, V66.4-9, V67.4-9, V68.4-9, V69.4-9, V70.4-9, V71.4-9, V72.4-9, V73.4-9, V74.4-9, V75.4-9, V76.4-9, V77.4-9, V78.4-9, V79.4-9, V80.3-5, V81.1, V82.1, V82.8-9, V83.0-3, V84.0-3, V85.0-3, V86.0-3, V87.0-9, V89.2-3, V89.9, V99

This table is based on information obtained from [1]

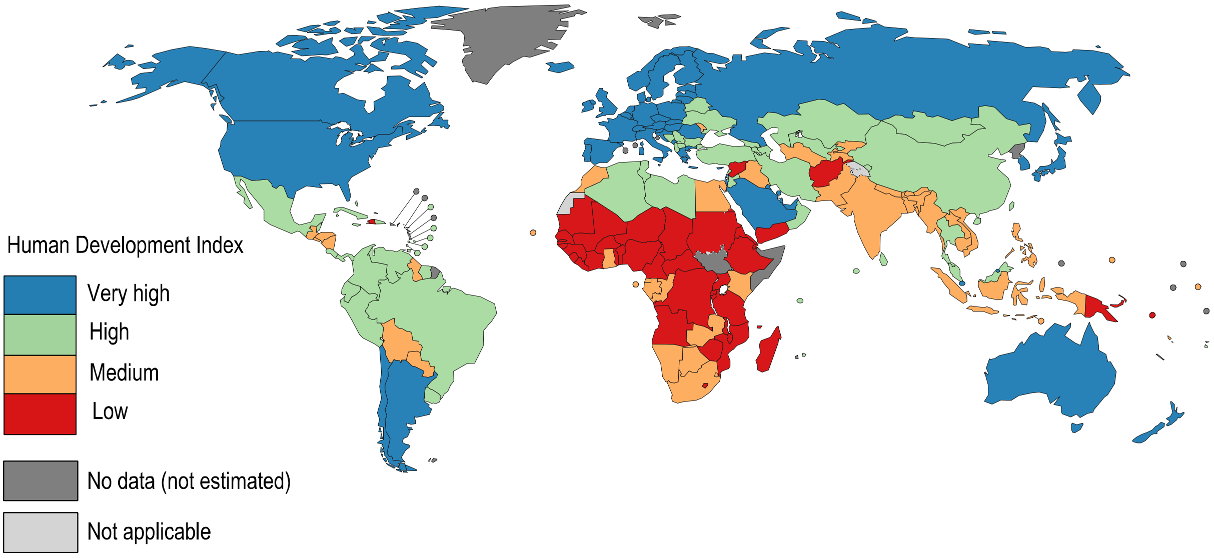

Figure A1. Map of Human Development Index scores

Supplemental Results

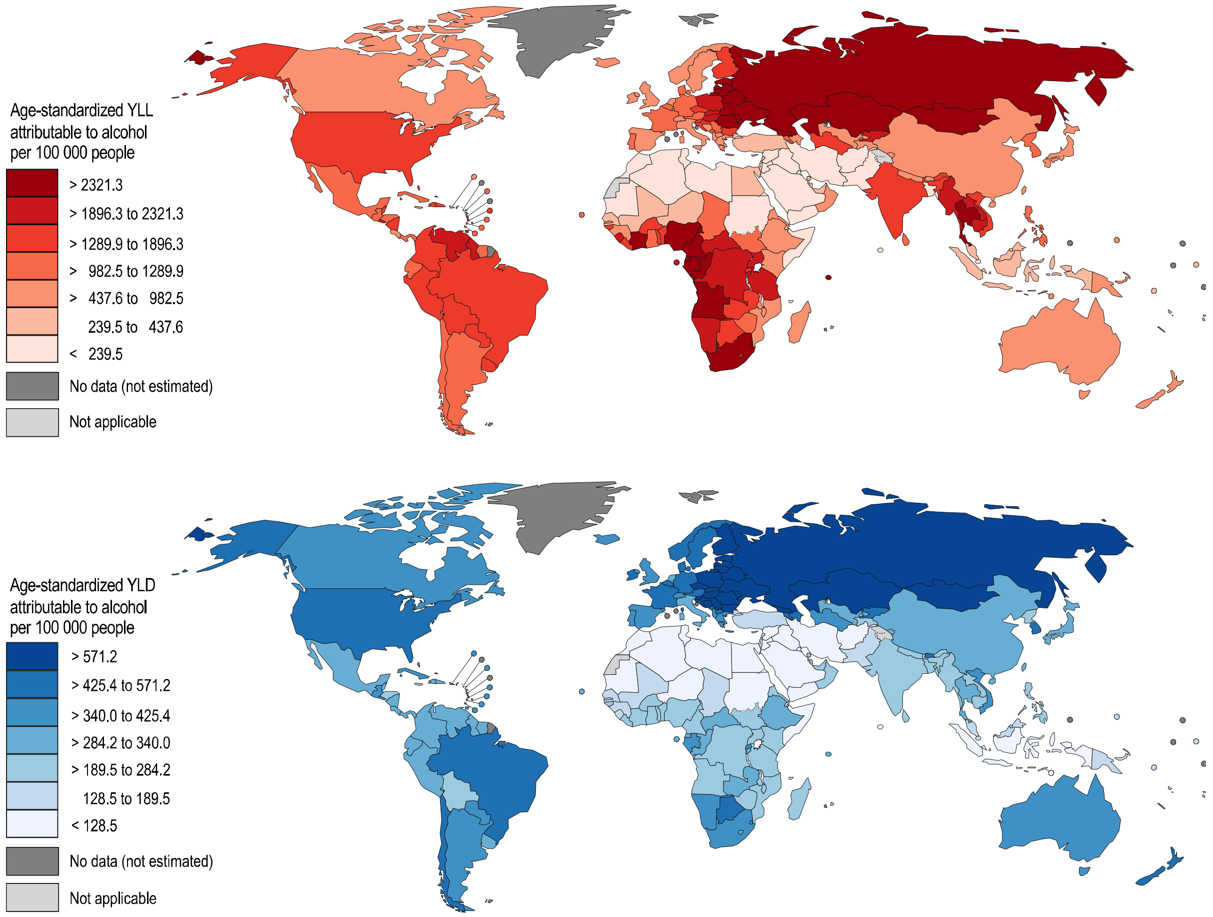

Figure A2. Alcohol-attributable years of life lost (YLL) and years lived with disability (YLD) globally in 2016 among people 0 to 69 years of age

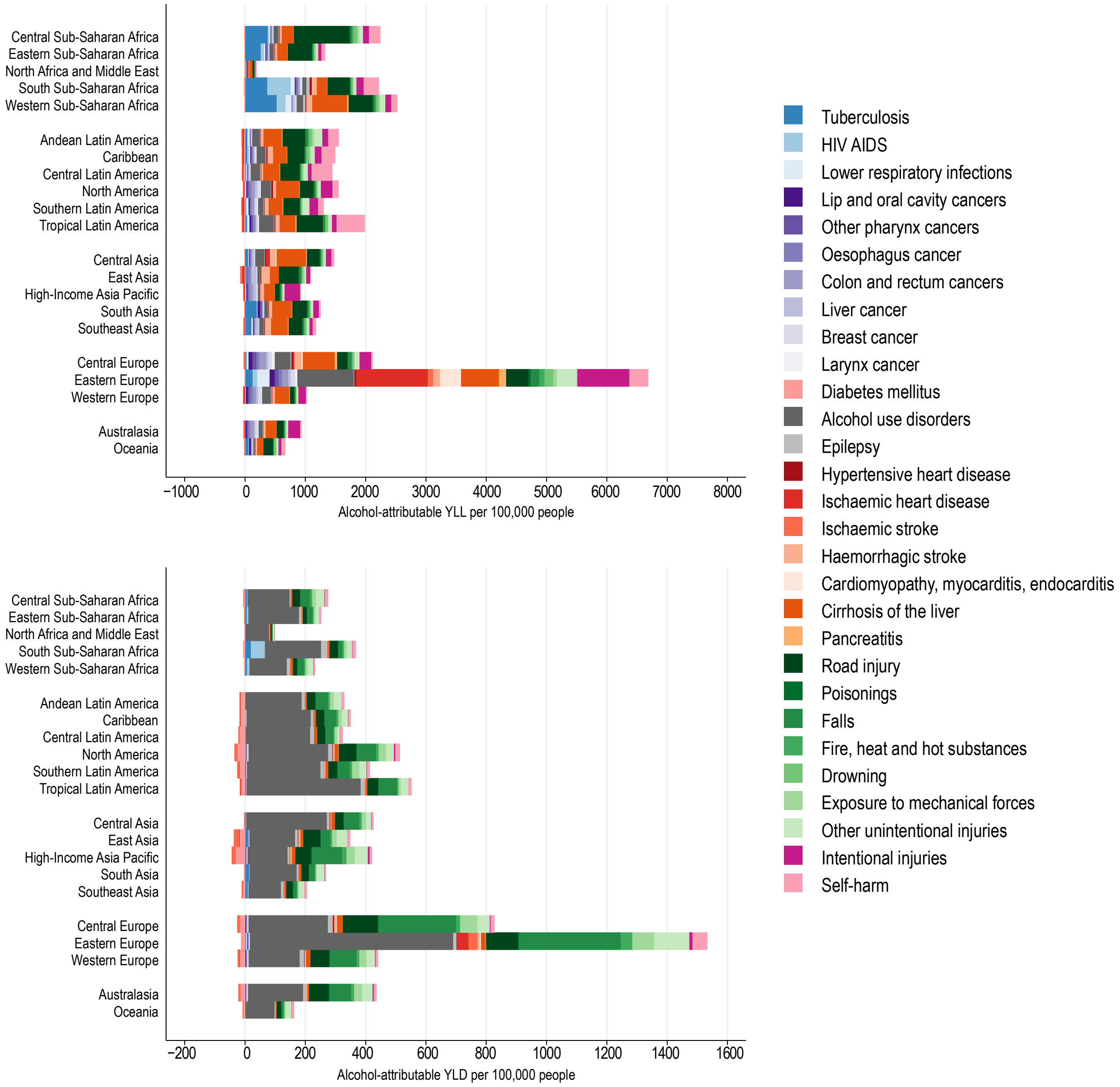

Figure A3. Alcohol-attributable years of life lost (YLL) and years lived with disability (YLD) among people 0 to 70 years of age by global burden of disease region

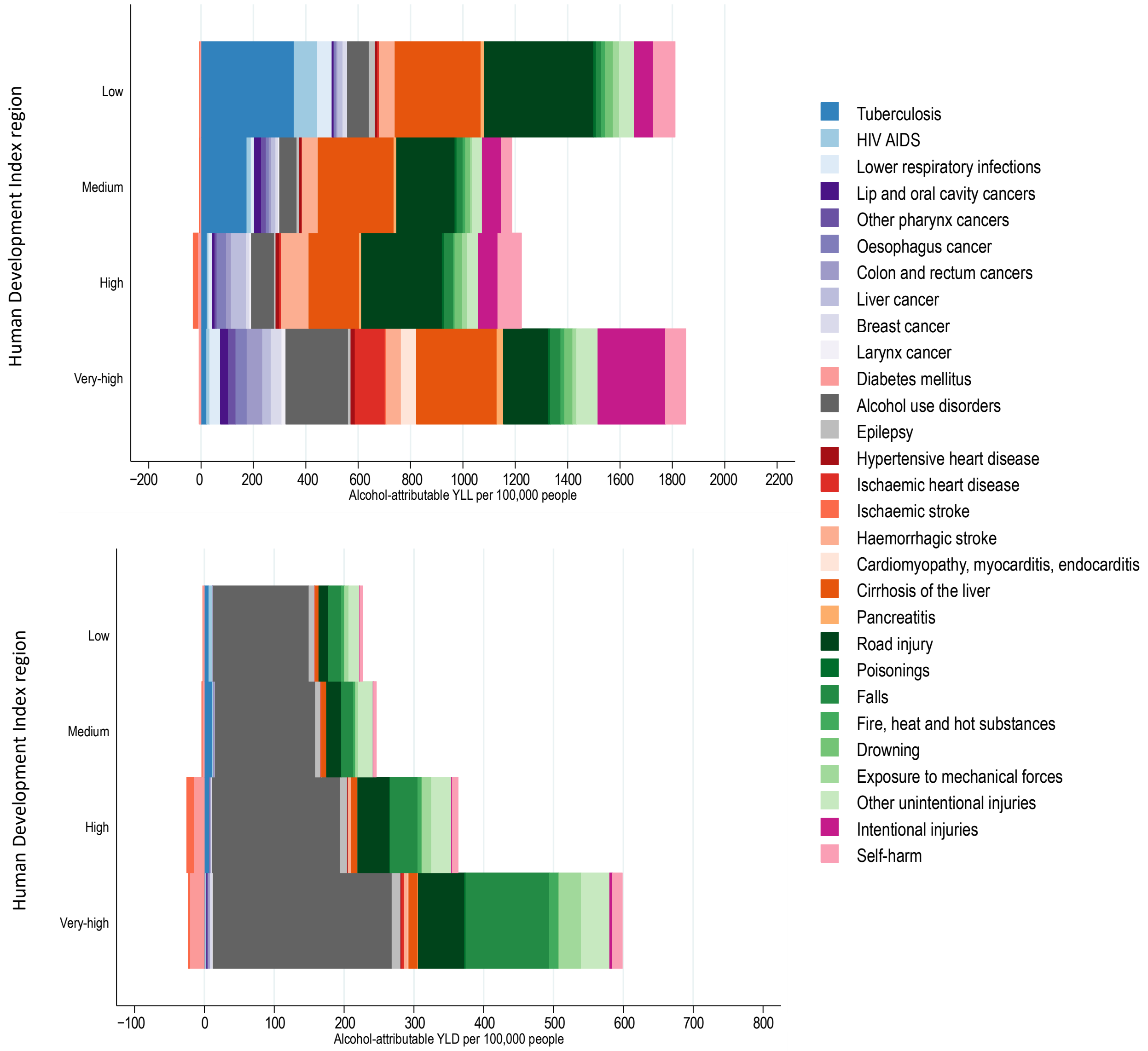

Figure A4. Alcohol-attributable years of life lost (YLL) and years lived with disability (YLD) among people 0 to 70 years of age by Human Development Index region

Table A2. Global alcohol-attributable deaths by age and cause in 2016 among men and 95% uncertainty intervals

Cause of disease or injury	Alcohol-attributable deaths								Population attributable fraction (%)							
	0 to 14	15 to 19	20 to 29	30 to 39	40 to 49	50 to 59	60 to 69	≥70	0 to 14	15 to 19	20 to 29	30 to 39	40 to 49	50 to 59	60 to 69	≥70
**All Causes**	20,149 (14,732, 33,236)	35,469 (27,123, 51,743)	202,169 (156,855, 256,557)	258,560 (212,454, 309,297)	316,489 (265,727, 368,739)	398,501 (342,386, 458,489)	404,431 (335,045, 478,517)	508,317 (344,428, 689,807)	0.6 (0.4, 1.0)	8.9 (6.8, 13.0)	17.3 (13.5, 22.0)	17.7 (14.5, 21.1)	15.5 (13.0, 18.1)	11.7 (10.0, 13.4)	7.5 (6.2, 8.9)	3.9 (2.7, 5.4)
**Communicable, maternal, perinatal and nutritional conditions**	0 (0, 0)	1,853 (787, 4,800)	22,296 (7,809, 37,774)	39,453 (16,759, 62,370)	48,583 (20,293, 77,737)	51,235 (19,968, 86,162)	46,459 (18,185, 82,289)	79,366 (33,863, 139,780)	0.0 (0.0, 0.0)	2.1 (0.9, 5.5)	9.4 (3.3, 15.9)	10.5 (4.5, 16.6)	11.7 (4.9, 18.8)	12.3 (4.8, 20.7)	9.6 (3.8, 17.0)	6.5 (2.8, 11.4)
Tuberculosis	0 (0, 0)	1,395 (286, 3,665)	17,868 (3,735, 32,892)	28,135 (6,072, 49,758)	35,615 (7,419, 63,437)	39,464 (8,377, 72,319)	33,937 (6,622, 66,748)	38,848 (6,999, 80,723)	0.0 (0.0, 0.0)	10.3 (2.1, 27.1)	27.0 (5.7, 49.8)	30.1 (6.5, 53.2)	30.3 (6.3, 53.9)	27.1 (5.7, 49.6)	22.4 (4.4, 44.1)	17.6 (3.2, 36.6)
HIV AIDS	0 (0, 0)	152 (91, 840)	2,124 (1,240, 3,286)	7,636 (4,454, 11,574)	8,006 (4,763, 12,007)	3,661 (2,173, 5,465)	1,088 (641, 1,634)	267 (147, 419)	0.0 (0.0, 0.0)	1.0 (0.6, 5.6)	3.8 (2.2, 5.8)	4.4 (2.6, 6.7)	4.7 (2.8, 7.1)	4.4 (2.6, 6.5)	3.9 (2.3, 5.9)	3.0 (1.7, 4.7)
Lower respiratory infections	0 (0, 0)	306 (88, 886)	2,304 (734, 4,182)	3,683 (1,722, 5,950)	4,962 (2,173, 8,187)	8,110 (3,504, 13,442)	11,434 (3,844, 20,789)	40,251 (10,349, 78,418)	0.0 (0.0, 0.0)	2.5 (0.7, 7.3)	7.7 (2.5, 14.0)	10.5 (4.9, 16.9)	10.5 (4.6, 17.3)	9.6 (4.2, 15.9)	7.3 (2.5, 13.3)	6.2 (1.6, 12.2)
**Noncommunicable diseases**	0 (0, 0)	3,441 (2,854, 4,706)	29,832 (25,204, 33,523)	85,505 (73,571, 93,955)	155,402 (134,200, 170,095)	248,371 (214,110, 274,866)	283,474 (228,885, 335,236)	348,141 (199,747, 507,580)	0.0 (0.0, 0.0)	3.4 (2.9, 4.7)	9.4 (7.9, 10.5)	14.5 (12.5, 16.0)	12.6 (10.9, 13.8)	9.4 (8.1, 10.5)	6.2 (5.0, 7.3)	3.1 (1.8, 4.5)
Malignant neoplasms	0 (0, 0)	0 (0, 0)	847 (630, 1,240)	7,456 (5,669, 9,313)	25,706 (20,033, 31,607)	63,262 (51,874, 74,965)	87,306 (72,530, 102,404)	119,076 (98,955, 143,516)	0.0 (0.0, 0.0)	0.0 (0.0, 0.0)	1.4 (1.0, 2.0)	6.3 (4.8, 7.8)	7.7 (6.0, 9.5)	7.9 (6.5, 9.4)	6.7 (5.5, 7.8)	4.9 (4.1, 5.9)
Lip and oral cavity cancer	0 (0, 0)	0 (0, 0)	184 (107, 287)	2,185 (1,520, 2,683)	5,639 (4,313, 6,609)	11,728 (9,589, 13,318)	12,702 (10,535, 14,516)	10,996 (9,203, 12,688)	0.0 (0.0, 0.0)	0.0 (0.0, 0.0)	8.6 (5.0, 13.5)	34.9 (24.2, 42.8)	38.3 (29.3, 44.9)	42.2 (34.5, 47.9)	41.7 (34.5, 47.6)	36.6 (30.7, 42.3)
Other pharynx cancers	0 (0, 0)	0 (0, 0)	46 (29, 71)	821 (595, 999)	3,831 (2,974, 4,470)	10,198 (8,420, 11,541)	11,422 (9,503, 13,030)	8,909 (7,310, 10,420)	0.0 (0.0, 0.0)	0.0 (0.0, 0.0)	8.7 (5.4, 13.3)	33.7 (24.4, 41.1)	39.0 (30.3, 45.5)	43.7 (36.1, 49.4)	42.3 (35.2, 48.2)	33.9 (27.8, 39.7)
Oesophagus cancer	0 (0, 0)	0 (0, 0)	37 (26, 55)	535 (438, 638)	3,934 (3,297, 4,546)	14,972 (12,453, 17,171)	24,455 (19,854, 28,474)	29,429 (23,116, 35,850)	0.0 (0.0, 0.0)	0.0 (0.0, 0.0)	4.5 (3.2, 6.8)	15.8 (12.9, 18.9)	22.7 (19.1, 26.3)	25.9 (21.6, 29.7)	25.6 (20.8, 29.8)	22.0 (17.3, 26.8)
Colon and rectum cancers	0 (0, 0)	0 (0, 0)	203 (170, 246)	1,345 (1,067, 1,642)	3,818 (3,011, 4,637)	10,210 (8,084, 12,396)	18,748 (14,927, 22,542)	41,823 (34,046, 50,055)	0.0 (0.0, 0.0)	0.0 (0.0, 0.0)	5.9 (5.0, 7.2)	13.9 (11.0, 17.0)	15.4 (12.2, 18.8)	17.2 (13.6, 20.9)	18.0 (14.3, 21.7)	18.2 (14.9, 21.8)
Liver cancer	0 (0, 0)	0 (0, 0)	377 (191, 703)	2,570 (1,201, 4,044)	8,484 (3,926, 13,483)	16,154 (7,835, 25,187)	19,978 (9,866, 31,078)	27,919 (13,882, 45,451)	0.0 (0.0, 0.0)	0.0 (0.0, 0.0)	5.4 (2.7, 10.1)	11.8 (5.5, 18.5)	12.3 (5.7, 19.6)	13.0 (6.3, 20.3)	13.3 (6.6, 20.7)	13.2 (6.6, 21.6)

Cause of disease or injury	Alcohol-attributable deaths								Population attributable fraction (%)							
	0 to 14	15 to 19	20 to 29	30 to 39	40 to 49	50 to 59	60 to 69	≥70	0 to 14	15 to 19	20 to 29	30 to 39	40 to 49	50 to 59	60 to 69	≥70
Breast cancer	0 (0, 0)	0 (0, 0)	0 (0, 0)	0 (0, 0)	0 (0, 0)	0 (0, 0)	0 (0, 0)	0 (0, 0)	0.0 (0.0, 0.0)	0.0 (0.0, 0.0)	0.0 (0.0, 1.4)	0.0 (0.0, 0.0)	0.0 (0.0, 0.0)	0.0 (0.0, 0.0)	0.0 (0.0, 0.0)	0.0 (0.0, 0.0)
Larynx cancer	0 (0, 0)	0 (0, 0)	10 (6, 17)	186 (134, 239)	1,347 (999, 1,704)	4,529 (3,470, 5,615)	6,516 (4,950, 8,111)	6,867 (5,093, 8,751)	0.0 (0.0, 0.0)	0.0 (0.0, 0.0)	4.7 (3.0, 7.5)	20.3 (14.6, 26.1)	23.1 (17.1, 29.2)	26.1 (20.0, 32.3)	25.7 (19.5, 32.0)	22.2 (16.5, 28.3)
Diabetes mellitus	0 (0, 0)	19 (-1, 86)	116 (16, 269)	265 (33, 637)	785 (89, 1,863)	1,976 (195, 4,574)	3,390 (207, 7,885)	7,281 (130, 17,083)	0.0 (0.0, 0.0)	1.1 (-0.1, 5.1)	1.8 (0.2, 4.1)	1.9 (0.2, 4.7)	1.9 (0.2, 4.6)	1.9 (0.2, 4.4)	1.8 (0.1, 4.3)	1.9 (0.0, 4.4)
Alcohol use disorders	0 (0, 0)	759 (759, 759)	6,655 (6,655, 6,655)	18,554 (18,554, 18,554)	26,935 (26,935, 26,935)	32,361 (32,361, 32,361)	23,264 (23,264, 23,264)	13,028 (13,028, 13,028)	100.0 (100.0, 100.0)	100.0 (100.0, 100.0)	100.0 (100.0, 100.0)	100.0 (100.0, 100.0)	100.0 (100.0, 100.0)	100.0 (100.0, 100.0)	100.0 (100.0, 100.0)	100.0 (100.0, 100.0)
Epilepsy	0 (0, 0)	512 (354, 830)	2,486 (1,812, 3,126)	2,387 (1,770, 2,958)	2,146 (1,614, 2,626)	1,730 (1,300, 2,150)	1,472 (1,098, 1,848)	1,931 (1,448, 2,428)	0.0 (0.0, 0.0)	6.8 (4.7, 11.1)	18.9 (13.8, 23.8)	22.0 (16.3, 27.3)	23.6 (17.7, 28.8)	23.1 (17.3, 28.7)	20.8 (15.5, 26.1)	18.7 (14.0, 23.5)
Cardiovascular diseases	0 (0, 0)	314 (216, 711)	2,510 (957, 4,300)	8,182 (3,879, 11,683)	17,651 (7,369, 23,798)	39,247 (17,423, 54,802)	59,917 (13,185, 99,780)	114,868 (-31,885, 254,655)	0.0 (0.0, 0.0)	1.6 (1.1, 3.5)	3.0 (1.1, 5.1)	4.0 (1.9, 5.7)	3.7 (1.5, 5.0)	3.6 (1.6, 5.0)	3.0 (0.7, 4.9)	2.2 (-0.6, 5.0)
Hypertensive heart disease	0 (0, 0)	27 (18, 42)	356 (262, 434)	1,011 (761, 1,218)	2,567 (1,959, 3,098)	5,797 (4,450, 7,062)	10,799 (8,011, 13,329)	32,890 (23,811, 41,297)	0.0 (0.0, 0.0)	4.5 (3.1, 7.1)	12.8 (9.4, 15.6)	14.8 (11.1, 17.8)	15.5 (11.8, 18.7)	14.9 (11.5, 18.2)	13.9 (10.3, 17.2)	13.4 (9.7, 16.8)
Ischaemic heart disease	0 (0, 0)	5 (-81, 137)	-265 (-1,688, 1,194)	3 (-3,930, 2,780)	111 (-8,996, 4,307)	3,215 (-18,086, 13,930)	6,521 (-37,063, 40,160)	13,506 (-126,768, 147,492)	0.0 (0.0, 0.0)	0.1 (-1.3, 2.3)	-0.7 (-4.8, 3.4)	0.0 (-3.5, 2.5)	0.0 (-3.1, 1.5)	0.5 (-2.8, 2.1)	0.6 (-3.3, 3.6)	0.5 (-4.6, 5.4)
Ischaemic stroke	0 (0, 0)	-6 (-10, 13)	-39 (-81, 92)	-55 (-171, 185)	-221 (-643, 468)	-576 (-2,201, 1,858)	-3,343 (-10,209, 5,654)	-13,223 (-38,925, 18,344)	0.0 (0.0, 0.0)	-0.9 (-1.5, 2.0)	-1.2 (-2.6, 3.0)	-0.8 (-2.6, 2.8)	-1.0 (-3.0, 2.2)	-0.7 (-2.7, 2.3)	-1.1 (-3.5, 1.9)	-1.4 (-4.2, 2.0)
Haemorrhagic stroke	0 (0, 0)	209 (129, 387)	1,791 (1,167, 2,451)	4,590 (3,049, 6,160)	11,943 (7,910, 16,036)	25,867 (17,447, 34,957)	42,191 (27,574, 58,890)	77,807 (49,833, 110,521)	0.0 (0.0, 0.0)	4.9 (3.0, 9.0)	9.9 (6.5, 13.6)	11.3 (7.5, 15.2)	11.8 (7.8, 15.8)	11.2 (7.5, 15.1)	10.6 (6.9, 14.7)	10.4 (6.6, 14.7)
Cardiomyopathy, myocarditis, endocarditis	0 (0, 0)	79 (63, 347)	665 (636, 1,098)	2,634 (2,611, 2,925)	3,251 (3,224, 3,527)	4,943 (4,901, 5,442)	3,750 (3,685, 4,474)	3,888 (3,793, 4,718)	0.0 (0.0, 0.0)	2.7 (2.1, 11.6)	6.9 (6.6, 11.5)	15.7 (15.6, 17.5)	14.5 (14.4, 15.7)	15.1 (15.0, 16.6)	9.7 (9.6, 11.6)	4.9 (4.8, 5.9)
Digestive diseases	0 (0, 0)	1,836 (1,305, 2,712)	17,208 (13,399, 20,124)	48,475 (39,069, 55,427)	80,832 (66,918, 91,560)	105,268 (87,864, 119,132)	101,609 (84,207, 117,002)	85,089 (68,637, 102,871)	0.0 (0.0, 0.0)	12.6 (9.0, 18.6)	31.0 (24.2, 36.3)	41.4 (33.4, 47.3)	43.1 (35.6, 48.8)	39.3 (32.8, 44.5)	32.0 (26.5, 36.8)	17.7 (14.3, 21.4)

Cause of disease or injury	Alcohol-attributable deaths								Population attributable fraction (%)							
	0 to 14	15 to 19	20 to 29	30 to 39	40 to 49	50 to 59	60 to 69	>70	0 to 14	15 to 19	20 to 29	30 to 39	40 to 49	50 to 59	60 to 69	>70
Cirrhosis of the liver	0 (0, 0)	1,759 (1,223, 2,627)	15,741 (12,091, 18,581)	45,030 (35,912, 51,768)	76,343 (62,603, 86,773)	100,871 (84,249, 114,775)	97,498 (80,347, 112,731)	79,490 (63,333, 96,837)	0.0 (0.0, 0.0)	26.0 (18.1, 38.9)	50.3 (38.6, 59.4)	54.9 (43.8, 63.1)	56.1 (46.0, 63.7)	53.9 (45.0, 61.4)	50.3 (41.5, 58.2)	42.2 (33.6, 51.4)
Pancreatitis	0 (0, 0)	78 (49, 120)	1,467 (951, 1,985)	3,445 (2,399, 4,480)	4,489 (3,012, 5,853)	4,397 (3,062, 5,661)	4,111 (2,777, 5,444)	5,599 (3,623, 7,591)	0.0 (0.0, 0.0)	14.6 (9.3, 22.7)	32.8 (21.3, 44.4)	37.5 (26.1, 48.7)	37.9 (25.4, 49.4)	36.8 (25.6, 47.3)	33.1 (22.4, 43.9)	30.7 (19.9, 41.6)
**Injuries**	20,149 (14,732, 33,236)	30,175 (21,659, 44,609)	150,041 (107,830, 200,272)	133,602 (100,058, 174,083)	112,504 (85,232, 145,942)	98,895 (75,736, 127,437)	74,498 (57,383, 98,241)	80,811 (58,750, 112,958)	5.4 (3.9, 8.8)	14.3 (10.3, 21.1)	24.6 (17.7, 32.8)	26.8 (20.1, 34.9)	28.2 (21.4, 36.6)	27.1 (20.7, 34.9)	22.9 (17.6, 30.2)	16.9 (12.3, 23.6)
Unintentional injuries	20,149 (14,732, 33,236)	21,937 (16,278, 33,032)	99,083 (73,241, 134,780)	86,513 (65,596, 115,400)	78,713 (59,661, 104,618)	71,809 (54,301, 95,384)	57,715 (42,806, 78,410)	67,220 (46,318, 97,269)	6.0 (4.4, 9.9)	17.2 (12.7, 25.9)	28.6 (21.1, 38.8)	30.1 (22.8, 40.2)	30.5 (23.2, 40.6)	28.5 (21.5, 37.8)	23.7 (17.6, 32.2)	17.1 (11.8, 24.8)
Road injury	20,149 (14,732, 33,236)	15,883 (10,467, 25,527)	67,508 (45,911, 99,117)	53,281 (36,627, 77,532)	46,060 (31,300, 67,384)	38,508 (25,887, 56,873)	28,483 (19,204, 42,909)	22,782 (15,463, 35,521)	22.8 (16.7, 37.7)	20.4 (13.5, 32.8)	30.3 (20.6, 44.5)	31.6 (21.7, 45.9)	31.9 (21.7, 46.7)	29.9 (20.1, 44.2)	27.1 (18.3, 40.8)	22.5 (15.3, 35.0)
Poisonings	0 (0, 0)	272 (133, 476)	1,933 (1,030, 2,874)	1,762 (1,020, 2,524)	1,774 (1,024, 2,558)	1,553 (944, 2,211)	1,323 (778, 1,956)	1,107 (596, 1,766)	0.0 (0.0, 0.0)	11.4 (5.6, 20.0)	25.2 (13.4, 37.5)	27.7 (16.0, 39.7)	27.3 (15.7, 39.3)	26.1 (15.9, 37.2)	20.7 (12.2, 30.5)	13.6 (7.3, 21.7)
Falls	0 (0, 0)	606 (312, 1,054)	4,609 (2,522, 6,736)	6,499 (3,768, 9,255)	8,259 (4,763, 11,827)	10,100 (5,800, 14,744)	10,677 (5,941, 16,146)	23,196 (12,134, 37,352)	0.0 (0.0, 0.0)	11.8 (6.1, 20.6)	25.6 (14.0, 37.4)	27.3 (15.8, 38.8)	28.1 (16.2, 40.2)	25.5 (14.7, 37.3)	19.5 (10.9, 29.5)	14.3 (7.5, 23.0)
Fire, heat and hot substances	0 (0, 0)	228 (115, 410)	1,642 (911, 2,427)	2,109 (1,335, 2,915)	1,926 (1,293, 2,564)	2,153 (1,502, 2,826)	1,806 (1,211, 2,471)	1,975 (1,183, 2,931)	0.0 (0.0, 0.0)	10.4 (5.2, 18.6)	22.9 (12.7, 33.9)	26.8 (17.0, 37.0)	29.9 (20.0, 39.7)	29.5 (20.6, 38.7)	24.0 (16.1, 32.8)	16.3 (9.8, 24.2)
Drowning	0 (0, 0)	2,277 (1,142, 3,884)	7,540 (4,147, 11,062)	6,322 (3,887, 8,843)	5,133 (3,135, 7,176)	4,401 (2,740, 6,195)	3,522 (2,069, 5,112)	3,262 (1,757, 5,028)	0.0 (0.0, 0.0)	12.8 (6.4, 21.8)	26.1 (14.3, 38.3)	29.3 (18.0, 41.0)	29.5 (18.0, 41.2)	27.2 (17.0, 38.4)	22.5 (13.2, 32.7)	16.9 (9.1, 26.1)
Exposure to mechanical forces	0 (0, 0)	665 (333, 1,146)	4,225 (2,248, 6,249)	4,105 (2,353, 5,871)	3,610 (2,094, 5,175)	3,076 (1,780, 4,421)	2,021 (1,131, 2,991)	1,311 (716, 2,035)	0.0 (0.0, 0.0)	12.1 (6.0, 20.8)	25.2 (13.4, 37.3)	27.6 (15.8, 39.4)	29.0 (16.8, 41.6)	27.0 (15.6, 38.8)	22.6 (12.6, 33.4)	15.1 (8.2, 23.4)
Other unintentional injuries	0 (0, 0)	2,005 (997, 3,464)	11,627 (6,231, 17,265)	12,435 (7,415, 17,659)	11,951 (7,315, 16,739)	12,017 (7,772, 16,478)	9,883 (6,128, 14,083)	13,588 (7,333, 20,938)	0.0 (0.0, 0.0)	11.9 (5.9, 20.5)	25.6 (13.7, 38.0)	28.5 (17.0, 40.4)	29.0 (17.8, 40.7)	27.8 (18.0, 38.1)	22.0 (13.6, 31.4)	16.9 (9.1, 26.0)
Intentional injuries	0 (0, 0)	8,238 (1,807, 16,220)	50,958 (14,632, 84,147)	47,089 (17,883, 73,555)	33,791 (12,656, 52,460)	27,086 (11,802, 41,388)	16,783 (7,019, 26,571)	13,591 (5,567, 22,947)	0.0 (0.0, 0.0)	9.9 (2.2, 19.5)	19.3 (5.5, 31.9)	22.3 (8.5, 34.9)	23.9 (9.0, 37.1)	23.9 (10.4, 36.6)	20.5 (8.6, 32.4)	15.9 (6.5, 26.8)

Cause of disease or injury	Alcohol-attributable deaths								Population attributable fraction (%)							
	0 to 14	15 to 19	20 to 29	30 to 39	40 to 49	50 to 59	60 to 69	≥70	0 to 14	15 to 19	20 to 29	30 to 39	40 to 49	50 to 59	60 to 69	≥70
Self-harm	0 (0, 0)	3,004 (773, 5,683)	23,219 (8,179, 37,159)	26,110 (11,455, 39,421)	21,727 (8,793, 32,915)	20,065 (8,892, 30,242)	13,618 (5,599, 21,664)	12,332 (4,966, 20,809)	0.0 (0.0, 0.0)	11.7 (3.0, 22.1)	24.0 (8.5, 38.4)	27.6 (12.1, 41.7)	28.3 (11.4, 42.8)	27.2 (12.1, 41.0)	22.3 (9.2, 35.5)	16.8 (6.7, 28.3)
Interpersonal violence	0 (0, 0)	5,234 (1,049, 10,502)	27,739 (6,355, 47,009)	20,980 (6,260, 34,227)	12,065 (3,800, 19,551)	7,021 (2,871, 11,117)	3,166 (1,430, 5,011)	1,258 (598, 2,098)	0.0 (0.0, 0.0)	11.9 (2.4, 23.9)	22.4 (5.1, 38.0)	24.2 (7.2, 39.4)	23.9 (7.5, 38.7)	22.0 (9.0, 34.8)	18.7 (8.5, 29.6)	12.5 (6.0, 20.9)

Table A3. Global alcohol-attributable deaths by age and cause in 2016 among women and 95% uncertainty intervals

Cause of disease or injury	Alcohol-attributable deaths								Population Attributable Fraction							
	0 to 14	15 to 19	20 to 29	30 to 39	40 to 49	50 to 59	60 to 69	≥70	0 to 14	15 to 19	20 to 29	30 to 39	40 to 49	50 to 59	60 to 69	≥70
**All Causes**	13,790 (10,118, 22,653)	12,250 (10,450, 18,165)	46,594 (38,785, 63,263)	58,897 (51,723, 79,052)	80,063 (71,461, 104,140)	111,947 (98,747, 149,414)	137,581 (111,400, 198,788)	362,619 (208,703, 583,096)	0.5 (0.3, 0.8)	4.2 (3.6, 6.2)	6.2 (5.2, 8.5)	6.4 (5.6, 8.6)	6.4 (5.7, 8.3)	5.3 (4.7, 7.1)	3.6 (2.9, 5.2)	2.6 (1.5, 4.1)
**Communicable, maternal, perinatal and nutritional conditions**	0 (0, 0)	1,220 (495, 3,903)	8,176 (3,121, 18,878)	11,044 (5,489, 25,852)	10,286 (4,905, 24,324)	9,411 (4,040, 24,141)	9,388 (4,058, 24,149)	23,115 (10,551, 51,856)	0.0 (0.0, 0.0)	1.1 (0.4, 3.4)	2.5 (1.0, 5.8)	3.0 (1.5, 7.1)	3.7 (1.8, 8.7)	3.8 (1.6, 9.7)	2.5 (1.1, 6.5)	1.7 (0.8, 3.9)
Tuberculosis	0 (0, 0)	904 (149, 2,976)	5,870 (840, 15,082)	6,841 (1,180, 17,691)	6,676 (1,163, 17,498)	6,633 (1,159, 18,976)	6,184 (1,030, 18,896)	7,915 (1,315, 25,899)	0.0 (0.0, 0.0)	6.6 (1.1, 21.8)	14.1 (2.0, 36.1)	14.1 (2.4, 36.6)	13.7 (2.4, 36.0)	10.4 (1.8, 29.9)	8.0 (1.3, 24.3)	5.3 (0.9, 17.5)
HIV AIDS	0 (0, 0)	117 (72, 734)	1,320 (769, 3,182)	2,930 (1,714, 8,490)	2,214 (1,314, 6,807)	691 (429, 2,627)	196 (120, 952)	35 (21, 372)	0.0 (0.0, 0.0)	0.9 (0.6, 5.7)	2.2 (1.3, 5.4)	2.2 (1.3, 6.5)	2.2 (1.3, 6.8)	1.6 (1.0, 6.2)	1.4 (0.8, 6.7)	0.8 (0.5, 8.3)
Lower respiratory infections	0 (0, 0)	199 (72, 755)	985 (402, 2,083)	1,274 (707, 2,520)	1,396 (745, 2,759)	2,087 (1,049, 4,336)	3,008 (1,297, 6,788)	15,165 (5,468, 31,829)	0.0 (0.0, 0.0)	1.8 (0.6, 6.7)	4.3 (1.8, 9.1)	5.4 (3.0, 10.7)	5.1 (2.7, 10.0)	3.8 (1.9, 8.0)	2.4 (1.0, 5.5)	2.1 (0.8, 4.4)
**Noncommunicable diseases**	0 (0, 0)	2,557 (2,061, 3,421)	12,472 (10,464, 15,567)	25,671 (22,483, 31,629)	47,969 (41,070, 61,110)	82,560 (69,135, 109,021)	109,169 (82,441, 160,357)	308,566 (156,486, 512,997)	0.0 (0.0, 0.0)	2.9 (2.4, 3.9)	5.4 (4.5, 6.8)	6.3 (5.6, 7.8)	5.7 (4.9, 7.3)	4.8 (4.0, 6.4)	3.4 (2.5, 4.9)	2.5 (1.3, 4.1)
Malignant neoplasms	0 (0, 0)	0 (0, 0)	247 (173, 384)	1,726 (1,337, 2,336)	4,947 (3,836, 6,683)	10,674 (8,450, 13,997)	15,313 (11,768, 20,398)	31,139 (22,486, 43,498)	0.0 (0.0, 0.0)	0.0 (0.0, 0.0)	0.5 (0.3, 0.7)	1.2 (0.9, 1.6)	1.4 (1.1, 1.9)	1.7 (1.4, 2.2)	1.8 (1.4, 2.4)	1.8 (1.3, 2.5)
Lip and oral cavity cancer	0 (0, 0)	0 (0, 0)	39 (26, 60)	357 (257, 476)	1,039 (732, 1,401)	2,004 (1,470, 2,681)	2,213 (1,625, 3,011)	3,089 (2,267, 4,310)	0.0 (0.0, 0.0)	0.0 (0.0, 0.0)	5.6 (3.7, 8.6)	16.6 (11.9, 22.1)	17.3 (12.2, 23.3)	18.2 (13.4, 24.4)	16.1 (11.8, 21.9)	14.9 (10.9, 20.8)
Other pharynx cancers	0 (0, 0)	0 (0, 0)	17 (11, 27)	154 (103, 210)	418 (289, 578)	827 (635, 1,095)	934 (716, 1,267)	1,013 (745, 1,440)	0.0 (0.0, 0.0)	0.0 (0.0, 0.0)	5.1 (3.3, 8.2)	15.5 (10.4, 21.2)	17.0 (11.7, 23.5)	19.2 (14.7, 25.4)	17.2 (13.2, 23.4)	14.1 (10.3, 20.0)
Oesophagus cancer	0 (0, 0)	0 (0, 0)	16 (9, 29)	177 (126, 254)	585 (430, 835)	1,544 (1,205, 2,070)	2,620 (1,930, 3,632)	4,641 (3,132, 7,096)	0.0 (0.0, 0.0)	0.0 (0.0, 0.0)	2.2 (1.3, 4.0)	8.1 (5.7, 11.5)	8.4 (6.2, 12.0)	9.6 (7.5, 12.9)	8.9 (6.5, 12.3)	7.2 (4.8, 10.9)
Colon and rectum cancers	0 (0, 0)	0 (0, 0)	45 (21, 85)	409 (243, 630)	1,006 (617, 1,541)	2,373 (1,458, 3,582)	3,539 (2,025, 5,525)	9,043 (4,352, 15,048)	0.0 (0.0, 0.0)	0.0 (0.0, 0.0)	1.5 (0.7, 2.9)	4.7 (2.8, 7.3)	5.0 (3.1, 7.7)	5.6 (3.5, 8.5)	5.1 (2.9, 7.9)	4.1 (2.0, 6.9)
Liver cancer	0 (0, 0)	0 (0, 0)	130 (64, 234)	629 (334, 1,065)	1,899 (1,007, 3,195)	3,926 (2,067, 6,562)	6,007 (3,079, 10,155)	13,354 (6,419, 22,872)	0.0 (0.0, 0.0)	0.0 (0.0, 0.0)	6.2 (3.1, 11.1)	10.6 (5.6, 17.9)	10.1 (5.4, 17.0)	10.4 (5.5, 17.5)	10.6 (5.5, 18.0)	11.0 (5.3, 18.8)

Cause of disease or injury	Alcohol-attributable deaths								Population Attributable Fraction							
	0 to 14	15 to 19	20 to 29	30 to 39	40 to 49	50 to 59	60 to 69	≥70	0 to 14	15 to 19	20 to 29	30 to 39	40 to 49	50 to 59	60 to 69	≥70
Breast cancer	0 (0, 0)	0 (0, 0)	178 (138, 281)	3,003 (2,335, 4,015)	6,743 (5,398, 8,675)	10,445 (8,500, 13,244)	9,238 (7,591, 11,667)	12,415 (10,202, 15,646)	0.0 (0.0, 0.0)	0.0 (0.0, 0.0)	2.5 (1.9, 3.9)	7.1 (5.5, 9.5)	7.4 (6.0, 9.6)	8.2 (6.6, 10.4)	7.4 (6.1, 9.4)	6.6 (5.4, 8.3)
Larynx cancer	0 (0, 0)	0 (0, 0)	3 (1, 6)	24 (15, 37)	71 (47, 107)	224 (156, 327)	333 (225, 503)	416 (246, 680)	0.0 (0.0, 0.0)	0.0 (0.0, 0.0)	3.0 (1.5, 6.0)	9.6 (5.9, 14.6)	10.2 (6.8, 15.3)	11.4 (7.9, 16.6)	10.6 (7.2, 16.0)	8.3 (4.9, 13.6)
Diabetes mellitus	0 (0, 0)	-61 (-88, 19)	-478 (-664, -233)	-946 (-1,296, -456)	-2,742 (-3,764, -1,361)	-6,449 (-8,999, -3,191)	-11,206 (-15,696, -5,123)	-27,034 (-38,551, -13,208)	0.0 (0.0, 0.0)	-3.2 (-4.6, 1.0)	-7.7 (-10.7, -3.8)	-8.2 (-11.3, -4.0)	-8.0 (-11.0, -4.0)	-6.8 (-9.5, -3.4)	-5.7 (-7.9, -2.6)	-5.3 (-7.5, -2.6)
Alcohol use disorders	0 (0, 0)	208 (208, 208)	1,093 (1,093, 1,093)	2,851 (2,851, 2,851)	4,287 (4,287, 4,287)	5,954 (5,954, 5,954)	4,838 (4,838, 4,838)	4,776 (4,776, 4,776)	100.0 (100.0, 100.0)	100.0 (100.0, 100.0)	100.0 (100.0, 100.0)	100.0 (100.0, 100.0)	100.0 (100.0, 100.0)	100.0 (100.0, 100.0)	100.0 (100.0, 100.0)	100.0 (100.0, 100.0)
Epilepsy	0 (0, 0)	182 (135, 352)	791 (581, 1,133)	632 (482, 911)	505 (385, 703)	447 (336, 620)	372 (281, 524)	820 (630, 1,138)	0.0 (0.0, 0.0)	4.3 (3.2, 8.2)	9.3 (6.9, 13.4)	9.5 (7.3, 13.8)	9.9 (7.6, 13.8)	8.3 (6.2, 11.5)	6.5 (4.9, 9.2)	6.2 (4.8, 8.6)
Cardiovascular diseases	0 (0, 0)	366 (229, 787)	1,818 (764, 3,773)	4,888 (2,524, 9,071)	12,448 (6,394, 22,877)	26,688 (13,914, 49,148)	47,791 (21,193, 93,024)	232,943 (75,420, 427,869)	0.0 (0.0, 0.0)	2.2 (1.4, 4.7)	3.4 (1.4, 7.1)	4.4 (2.3, 8.2)	4.8 (2.4, 8.7)	4.3 (2.3, 8.0)	3.4 (1.5, 6.5)	3.7 (1.2, 6.8)
Hypertensive heart disease	0 (0, 0)	13 (6, 38)	101 (46, 194)	249 (119, 510)	637 (304, 1,339)	1,281 (617, 2,821)	2,048 (961, 5,004)	8,681 (4,013, 19,971)	0.0 (0.0, 0.0)	1.9 (0.9, 5.4)	4.5 (2.1, 8.7)	4.5 (2.2, 9.2)	4.5 (2.1, 9.4)	3.5 (1.7, 7.7)	2.6 (1.2, 6.3)	2.4 (1.1, 5.4)
Ischaemic heart disease	0 (0, 0)	81 (-9, 256)	403 (-554, 1,822)	1,193 (-975, 4,316)	2,916 (-2,416, 10,587)	9,439 (-2,897, 25,841)	25,481 (-3,254, 56,776)	188,152 (21,606, 327,640)	0.0 (0.0, 0.0)	1.9 (-0.2, 6.1)	2.3 (-3.1, 10.2)	2.7 (-2.2, 9.8)	2.5 (-2.1, 9.1)	3.2 (-1.0, 8.9)	3.7 (-0.5, 8.2)	5.7 (0.7, 9.9)
Ischaemic stroke	0 (0, 0)	-5 (-9, 141)	-36 (-72, 553)	-119 (-189, 468)	-618 (-963, 224)	-2,247 (-3,627, 112)	-6,937 (-12,936, 4,583)	-31,845 (-78,376, 50,628)	0.0 (0.0, 0.0)	-0.5 (-1.0, 15.0)	-1.3 (-2.6, 20.2)	-2.3 (-3.7, 9.2)	-4.0 (-6.2, 1.4)	-4.4 (-7.1, 0.2)	-3.2 (-5.9, 2.1)	-2.7 (-6.7, 4.3)
Haemorrhagic stroke	0 (0, 0)	267 (150, 500)	1,253 (727, 2,112)	3,178 (1,898, 5,327)	9,025 (5,353, 15,007)	17,338 (10,438, 28,877)	26,388 (15,686, 44,505)	65,182 (40,076, 108,578)	0.0 (0.0, 0.0)	6.6 (3.7, 12.4)	11.2 (6.5, 18.8)	11.3 (6.7, 18.9)	11.9 (7.1, 19.8)	10.2 (6.2, 17.0)	8.5 (5.1, 14.4)	8.1 (5.0, 13.4)
Cardiomyopathy, myocarditis, endocarditis	0 (0, 0)	9 (9, 10)	98 (98, 100)	387 (387, 389)	488 (488, 490)	876 (876, 878)	812 (812, 816)	2,774 (2,773, 2,784)	0.0 (0.0, 0.0)	0.5 (0.5, 0.6)	1.7 (1.7, 1.8)	4.9 (4.9, 4.9)	4.9 (4.9, 4.9)	5.9 (5.9, 6.0)	3.7 (3.7, 3.7)	2.9 (2.9, 2.9)
Digestive diseases	0 (0, 0)	1,861 (1,422, 2,421)	8,819 (7,189, 10,418)	13,492 (11,637, 15,456)	21,709 (18,957, 24,648)	34,578 (30,001, 39,883)	42,489 (36,019, 50,616)	53,091 (44,269, 66,828)	0.0 (0.0, 0.0)	12.9 (9.9, 16.8)	24.3 (19.8, 28.7)	28.7 (24.8, 32.9)	31.2 (27.3, 35.4)	27.7 (24.0, 32.0)	21.4 (18.2, 25.5)	10.3 (8.6, 12.9)

Cause of disease or injury	Alcohol-attributable deaths								Population Attributable Fraction							
	0 to 14	15 to 19	20 to 29	30 to 39	40 to 49	50 to 59	60 to 69	≥70	0 to 14	15 to 19	20 to 29	30 to 39	40 to 49	50 to 59	60 to 69	≥70
Cirrhosis of the liver	0 (0, 0)	1,834 (1,396, 2,387)	8,628 (7,031, 10,066)	13,115 (11,262, 14,902)	21,187 (18,396, 23,862)	33,831 (29,354, 38,834)	41,607 (35,194, 49,366)	51,205 (42,255, 64,286)	0.0 (0.0, 0.0)	32.1 (24.5, 41.8)	48.5 (39.5, 56.6)	51.1 (43.9, 58.1)	52.9 (45.9, 59.5)	48.6 (42.1, 55.7)	42.7 (36.1, 50.7)	34.6 (28.6, 43.4)
Pancreatitis	0 (0, 0)	27 (6, 77)	191 (49, 508)	377 (166, 843)	522 (162, 1,316)	747 (285, 1,786)	882 (354, 2,039)	1,886 (598, 4,418)	0.0 (0.0, 0.0)	6.4 (1.4, 18.6)	11.9 (3.0, 31.7)	14.2 (6.2, 31.7)	13.0 (4.0, 32.7)	12.0 (4.6, 28.6)	9.7 (3.9, 22.4)	8.5 (2.7, 19.9)
**Injuries**	13,790 (10,118, 22,653)	8,474 (6,711, 12,307)	25,946 (19,553, 35,949)	22,182 (17,616, 30,048)	21,808 (17,292, 29,452)	19,975 (16,617, 26,898)	19,024 (15,921, 26,394)	30,938 (24,660, 45,849)	5.1 (3.8, 8.4)	9.2 (7.3, 13.4)	13.4 (10.1, 18.5)	14.8 (11.7, 20.0)	16.2 (12.9, 21.9)	13.5 (11.3, 18.2)	10.3 (8.6, 14.3)	6.9 (5.5, 10.2)
Unintentional injuries	13,790 (10,118, 22,653)	6,366 (5,250, 9,226)	17,178 (14,096, 23,612)	14,908 (12,397, 20,312)	15,748 (13,024, 21,517)	15,197 (12,912, 20,950)	15,452 (12,963, 21,832)	27,184 (21,108, 41,461)	5.9 (4.3, 9.6)	13.1 (10.8, 19.0)	17.6 (14.4, 24.1)	17.6 (14.7, 24.0)	18.7 (15.4, 25.5)	15.0 (12.7, 20.7)	11.0 (9.2, 15.5)	6.9 (5.4, 10.5)
Road injury	13,790 (10,118, 22,653)	4,649 (3,650, 6,881)	11,232 (8,852, 15,974)	9,227 (7,422, 13,092)	10,069 (8,059, 14,467)	9,540 (7,583, 14,028)	9,315 (7,393, 13,919)	10,292 (8,260, 15,537)	23.4 (17.2, 38.5)	19.6 (15.4, 29.0)	23.5 (18.5, 33.4)	23.2 (18.6, 32.9)	23.4 (18.7, 33.6)	20.6 (16.4, 30.3)	19.5 (15.5, 29.1)	17.8 (14.3, 26.9)
Poisonings	0 (0, 0)	138 (70, 267)	610 (322, 1,011)	511 (288, 827)	568 (309, 926)	416 (234, 678)	395 (224, 665)	373 (246, 623)	0.0 (0.0, 0.0)	7.9 (4.1, 15.4)	13.2 (7.0, 21.8)	14.3 (8.0, 23.1)	13.4 (7.3, 21.8)	9.7 (5.5, 15.9)	6.6 (3.8, 11.2)	5.0 (3.3, 8.3)
Falls	0 (0, 0)	203 (108, 366)	789 (443, 1,261)	848 (505, 1,330)	1,081 (623, 1,717)	1,424 (826, 2,332)	2,137 (1,261, 3,709)	8,942 (5,436, 15,845)	0.0 (0.0, 0.0)	7.5 (4.0, 13.6)	13.1 (7.4, 20.9)	14.2 (8.4, 22.2)	14.6 (8.4, 23.1)	9.1 (5.3, 14.9)	5.3 (3.2, 9.3)	4.5 (2.7, 7.9)
Fire, heat and hot substances	0 (0, 0)	303 (141, 555)	1,172 (617, 1,960)	997 (584, 1,614)	688 (435, 1,081)	621 (437, 945)	556 (392, 870)	985 (751, 1,528)	0.0 (0.0, 0.0)	5.3 (2.5, 9.7)	9.8 (5.1, 16.4)	10.1 (5.9, 16.3)	11.3 (7.2, 17.8)	9.6 (6.8, 14.6)	7.2 (5.1, 11.3)	6.0 (4.6, 9.3)
Drowning	0 (0, 0)	431 (225, 784)	1,013 (560, 1,642)	892 (537, 1,388)	850 (495, 1,340)	707 (412, 1,141)	701 (399, 1,194)	1,058 (622, 1,883)	0.0 (0.0, 0.0)	7.8 (4.1, 14.1)	13.3 (7.4, 21.6)	14.4 (8.7, 22.4)	15.4 (9.0, 24.3)	10.9 (6.3, 17.6)	8.0 (4.5, 13.5)	5.7 (3.4, 10.2)
Exposure to mechanical forces	0 (0, 0)	121 (63, 224)	430 (234, 698)	468 (266, 750)	503 (281, 821)	366 (218, 592)	290 (168, 496)	323 (210, 580)	0.0 (0.0, 0.0)	7.0 (3.6, 12.9)	12.4 (6.7, 20.1)	13.6 (7.7, 21.8)	14.4 (8.0, 23.5)	10.4 (6.2, 16.8)	7.0 (4.1, 12.1)	4.7 (3.1, 8.5)
Other unintentional injuries	0 (0, 0)	521 (272, 970)	1,932 (1,079, 3,160)	1,964 (1,231, 3,076)	1,989 (1,281, 3,055)	2,124 (1,479, 3,114)	2,059 (1,411, 3,157)	5,211 (3,167, 8,645)	0.0 (0.0, 0.0)	7.0 (3.7, 13.1)	11.9 (6.7, 19.5)	12.7 (8.0, 19.9)	13.8 (8.9, 21.2)	11.4 (8.0, 16.7)	7.9 (5.4, 12.0)	6.1 (3.7, 10.1)
Intentional injuries	0 (0, 0)	2,108 (698, 4,244)	8,768 (3,100, 16,086)	7,274 (3,379, 12,356)	6,060 (2,700, 10,395)	4,778 (2,444, 7,947)	3,572 (1,892, 6,038)	3,755 (2,400, 6,078)	0.0 (0.0, 0.0)	4.9 (1.6, 9.8)	9.1 (3.2, 16.7)	11.1 (5.2, 18.9)	12.1 (5.4, 20.7)	10.3 (5.3, 17.2)	8.2 (4.4, 13.9)	6.8 (4.4, 11.1)
Self-harm	0 (0, 0)	1,487 (460, 2,928)	6,258 (2,122, 11,576)	4,975 (2,120, 8,590)	4,480 (1,802, 7,883)	3,702 (1,688, 6,403)	2,890 (1,385, 5,061)	3,151 (1,904, 5,289)	0.0 (0.0, 0.0)	5.5 (1.7, 10.8)	9.9 (3.4, 18.3)	12.3 (5.3, 21.3)	13.4 (5.4, 23.6)	10.7 (4.9, 18.5)	8.2 (3.9, 14.4)	6.7 (4.0, 11.2)

Cause of disease or injury	Alcohol-attributable deaths								Population Attributable Fraction							
	0 to 14	15 to 19	20 to 29	30 to 39	40 to 49	50 to 59	60 to 69	≥70	0 to 14	15 to 19	20 to 29	30 to 39	40 to 49	50 to 59	60 to 69	≥70
Interpersonal violence	0 (0, 0)	621 (196, 1,296)	2,510 (956, 4,564)	2,299 (1,226, 3,788)	1,580 (896, 2,518)	1,075 (762, 1,520)	682 (509, 948)	603 (496, 803)	0.0 (0.0, 0.0)	6.8 (2.2, 14.2)	11.4 (4.3, 20.7)	13.3 (7.1, 21.9)	13.8 (7.8, 21.9)	13.0 (9.2, 18.4)	10.8 (8.1, 15.0)	9.0 (7.4, 12.0)

Table A4. Global alcohol-attributable deaths by age and cause in 2016 and 95% uncertainty intervals

Cause of disease or injury	Alcohol-attributable deaths								Population Attributable Fraction							
	0 to 14	15 to 19	20 to 29	30 to 39	40 to 49	50 to 59	60 to 69	≥70	0 to 14	15 to 19	20 to 29	30 to 39	40 to 49	50 to 59	60 to 69	≥70
**All Causes**	33,939 (24,852, 55,874)	47,719 (39,186, 67,362)	248,762 (202,427, 310,761)	317,457 (272,876, 378,621)	396,552 (345,509, 458,861)	510,448 (450,236, 586,110)	542,012 (468,276, 645,149)	870,937 (651,326, 1,176,699)	0.5 (0.4, 0.9)	6.9 (5.7, 9.7)	13.0 (10.6, 16.2)	13.3 (11.5, 15.9)	12.0 (10.5, 13.9)	9.2 (8.2, 10.6)	5.9 (5.1, 7.0)	3.2 (2.4, 4.3)
**Communicable, maternal, perinatal and nutritional conditions**	0 (0, 0)	3,073 (1,693, 7,873)	30,471 (14,692, 50,248)	50,497 (27,436, 78,838)	58,869 (30,134, 92,324)	60,646 (28,789, 100,513)	55,847 (27,698, 95,539)	102,481 (57,267, 173,095)	0.0 (0.0, 0.0)	1.5 (0.8, 3.9)	5.4 (2.6, 9.0)	6.8 (3.7, 10.6)	8.5 (4.3, 13.3)	9.1 (4.3, 15.1)	6.5 (3.2, 11.2)	4.0 (2.2, 6.7)
Tuberculosis	0 (0, 0)	2,299 (873, 5,717)	23,738 (8,111, 42,540)	34,975 (11,908, 61,189)	42,291 (13,822, 74,498)	46,097 (14,689, 84,478)	40,121 (12,536, 78,116)	46,762 (14,487, 96,113)	0.0 (0.0, 0.0)	8.5 (3.2, 21.0)	22.0 (7.5, 39.5)	24.7 (8.4, 43.1)	25.4 (8.3, 44.8)	22.0 (7.0, 40.3)	17.5 (5.5, 34.1)	12.7 (3.9, 26.1)
HIV AIDS	0 (0, 0)	269 (189, 1,428)	3,444 (2,381, 5,608)	10,566 (7,171, 17,164)	10,220 (6,976, 16,221)	4,352 (2,946, 7,051)	1,284 (849, 2,182)	302 (190, 660)	0.0 (0.0, 0.0)	1.0 (0.7, 5.1)	3.0 (2.1, 4.9)	3.5 (2.4, 5.6)	3.8 (2.6, 6.0)	3.5 (2.3, 5.6)	3.1 (2.0, 5.2)	2.3 (1.4, 4.9)
Lower respiratory infections	0 (0, 0)	505 (258, 1,550)	3,289 (1,672, 5,610)	4,956 (3,009, 7,663)	6,358 (3,690, 10,054)	10,197 (5,798, 16,213)	14,442 (7,123, 24,838)	55,417 (25,480, 98,175)	0.0 (0.0, 0.0)	2.2 (1.1, 6.6)	6.2 (3.2, 10.6)	8.4 (5.1, 13.0)	8.5 (4.9, 13.4)	7.4 (4.2, 11.7)	5.1 (2.5, 8.9)	4.1 (1.9, 7.2)
**Noncommunicable diseases**	0 (0, 0)	5,997 (5,182, 7,899)	42,304 (36,931, 47,710)	111,176 (98,486, 123,455)	203,371 (179,943, 225,818)	330,932 (292,838, 372,508)	392,643 (330,041, 470,741)	656,706 (437,980, 925,953)	0.0 (0.0, 0.0)	3.2 (2.8, 4.2)	7.7 (6.7, 8.7)	11.2 (9.9, 12.4)	9.8 (8.7, 10.9)	7.6 (6.7, 8.6)	5.0 (4.2, 6.0)	2.8 (1.9, 3.9)
Malignant neoplasms	0 (0, 0)	0 (0, 0)	1,094 (864, 1,551)	9,183 (7,384, 11,258)	30,653 (24,867, 37,282)	73,936 (62,154, 87,218)	102,619 (86,842, 120,572)	150,215 (127,076, 180,611)	0.0 (0.0, 0.0)	0.0 (0.0, 0.0)	0.9 (0.7, 1.3)	3.4 (2.7, 4.2)	4.5 (3.7, 5.5)	5.2 (4.4, 6.1)	4.8 (4.0, 5.6)	3.6 (3.0, 4.3)
Lip and oral cavity cancer	0 (0, 0)	0 (0, 0)	224 (145, 336)	2,542 (1,815, 3,107)	6,678 (5,176, 7,865)	13,732 (11,277, 15,711)	14,915 (12,470, 17,164)	14,085 (11,956, 16,436)	0.0 (0.0, 0.0)	0.0 (0.0, 0.0)	7.9 (5.1, 11.9)	30.2 (21.6, 36.9)	32.2 (25.0, 38.0)	35.4 (29.1, 40.5)	33.7 (28.2, 38.8)	27.8 (23.6, 32.4)
Other pharynx cancers	0 (0, 0)	0 (0, 0)	63 (42, 93)	975 (714, 1,187)	4,250 (3,324, 4,981)	11,026 (9,184, 12,503)	12,356 (10,344, 14,072)	9,922 (8,245, 11,608)	0.0 (0.0, 0.0)	0.0 (0.0, 0.0)	7.3 (4.9, 10.8)	28.5 (20.8, 34.7)	34.6 (27.1, 40.6)	39.9 (33.2, 45.2)	38.1 (31.9, 43.4)	29.6 (24.6, 34.7)
Oesophagus cancer	0 (0, 0)	0 (0, 0)	53 (39, 76)	712 (587, 854)	4,519 (3,834, 5,243)	16,516 (13,851, 18,976)	27,076 (22,173, 31,703)	34,070 (27,083, 41,610)	0.0 (0.0, 0.0)	0.0 (0.0, 0.0)	3.5 (2.5, 5.0)	12.8 (10.5, 15.3)	18.6 (15.8, 21.6)	22.4 (18.8, 25.7)	21.7 (17.7, 25.4)	17.2 (13.6, 21.0)
Colon and rectum cancers	0 (0, 0)	0 (0, 0)	248 (208, 315)	1,754 (1,431, 2,164)	4,823 (3,937, 5,885)	12,582 (10,336, 15,139)	22,287 (18,238, 26,701)	50,866 (41,686, 62,116)	0.0 (0.0, 0.0)	0.0 (0.0, 0.0)	3.9 (3.3, 5.0)	9.6 (7.8, 11.8)	10.8 (8.8, 13.2)	12.4 (10.2, 14.9)	12.8 (10.5, 15.3)	11.4 (9.3, 13.9)
Liver cancer	0 (0, 0)	0 (0, 0)	507 (308, 869)	3,199 (1,818, 4,767)	10,383 (5,787, 15,756)	20,080 (11,445, 30,033)	25,985 (15,450, 38,653)	41,273 (25,034, 61,613)	0.0 (0.0, 0.0)	0.0 (0.0, 0.0)	5.6 (3.4, 9.6)	11.5 (6.5, 17.2)	11.8 (6.6, 18.0)	12.4 (7.1, 18.6)	12.6 (7.5, 18.7)	12.4 (7.5, 18.5)

Cause of disease or injury	Alcohol-attributable deaths								Population Attributable Fraction							
	0 to 14	15 to 19	20 to 29	30 to 39	40 to 49	50 to 59	60 to 69	≥70	0 to 14	15 to 19	20 to 29	30 to 39	40 to 49	50 to 59	60 to 69	≥70
Breast cancer	0 (0, 0)	0 (0, 0)	178 (138, 282)	3,003 (2,335, 4,015)	6,743 (5,398, 8,675)	10,445 (8,500, 13,244)	9,238 (7,591, 11,667)	12,415 (10,202, 15,646)	0.0 (0.0, 0.0)	0.0 (0.0, 0.0)	2.4 (1.9, 3.9)	7.1 (5.5, 9.5)	7.4 (5.9, 9.5)	8.1 (6.6, 10.3)	7.4 (6.1, 9.3)	6.5 (5.3, 8.2)
Larynx cancer	0 (0, 0)	0 (0, 0)	13 (10, 20)	210 (156, 268)	1,418 (1,070, 1,784)	4,753 (3,683, 5,850)	6,849 (5,276, 8,491)	7,283 (5,461, 9,216)	0.0 (0.0, 0.0)	0.0 (0.0, 0.0)	4.2 (3.0, 6.4)	17.9 (13.3, 22.9)	21.7 (16.4, 27.3)	24.6 (19.0, 30.3)	24.0 (18.5, 29.8)	20.3 (15.2, 25.7)
Diabetes mellitus	0 (0, 0)	-41 (-74, 98)	-362 (-564, -59)	-681 (-1,081, -14)	-1,958 (-3,148, -99)	-4,474 (-7,362, -163)	-7,816 (-13,080, 118)	-19,753 (-33,296, -1,782)	0.0 (0.0, 0.0)	-1.1 (-2.0, 2.7)	-2.8 (-4.4, -0.5)	-2.7 (-4.3, -0.1)	-2.6 (-4.2, -0.1)	-2.3 (-3.7, -0.1)	-2.0 (-3.4, 0.0)	-2.2 (-3.7, -0.2)
Alcohol use disorders	0 (0, 0)	967 (967, 967)	7,748 (7,748, 7,748)	21,406 (21,406, 21,406)	31,222 (31,222, 31,222)	38,315 (38,315, 38,315)	28,103 (28,103, 28,103)	17,804 (17,804, 17,804)	100.0 (100.0, 100.0)	100.0 (100.0, 100.0)	100.0 (100.0, 100.0)	100.0 (100.0, 100.0)	100.0 (100.0, 100.0)	100.0 (100.0, 100.0)	100.0 (100.0, 100.0)	100.0 (100.0, 100.0)
Epilepsy	0 (0, 0)	694 (538, 1,132)	3,278 (2,562, 4,068)	3,019 (2,376, 3,724)	2,651 (2,097, 3,223)	2,177 (1,724, 2,634)	1,844 (1,449, 2,275)	2,751 (2,205, 3,371)	0.0 (0.0, 0.0)	5.9 (4.6, 9.6)	15.2 (11.8, 18.8)	17.3 (13.6, 21.3)	18.7 (14.8, 22.7)	16.9 (13.4, 20.4)	14.4 (11.3, 17.8)	11.7 (9.4, 14.3)
Cardiovascular diseases	0 (0, 0)	680 (517, 1,337)	4,328 (2,498, 7,061)	13,070 (8,287, 18,593)	30,099 (18,341, 41,639)	65,934 (41,085, 91,890)	107,708 (55,856, 168,906)	347,811 (134,789, 598,110)	0.0 (0.0, 0.0)	1.8 (1.4, 3.6)	3.2 (1.8, 5.2)	4.2 (2.6, 5.9)	4.1 (2.5, 5.6)	3.8 (2.4, 5.4)	3.1 (1.6, 4.9)	3.0 (1.2, 5.2)
Hypertensive heart disease	0 (0, 0)	40 (29, 75)	457 (350, 590)	1,260 (989, 1,610)	3,204 (2,524, 4,102)	7,079 (5,590, 9,114)	12,846 (9,947, 16,817)	41,571 (31,747, 55,910)	0.0 (0.0, 0.0)	3.1 (2.2, 5.8)	9.1 (7.0, 11.8)	10.2 (8.0, 13.0)	10.4 (8.2, 13.3)	9.4 (7.4, 12.1)	8.2 (6.4, 10.7)	6.8 (5.2, 9.1)
Ischaemic heart disease	0 (0, 0)	85 (-40, 325)	139 (-1,468, 2,186)	1,195 (-3,160, 5,273)	3,027 (-7,738, 10,852)	12,654 (-10,934, 31,285)	32,002 (-17,488, 77,544)	201,657 (-11,761, 401,402)	0.0 (0.0, 0.0)	0.8 (-0.4, 3.2)	0.3 (-2.8, 4.1)	0.8 (-2.0, 3.4)	0.8 (-1.9, 2.7)	1.3 (-1.2, 3.3)	1.8 (-1.0, 4.3)	3.3 (-0.2, 6.6)
Ischaemic stroke	0 (0, 0)	-11 (-16, 138)	-75 (-125, 565)	-174 (-297, 521)	-838 (-1,329, 444)	-2,823 (-4,943, 1,082)	-10,280 (-19,119, 6,159)	-45,068 (-97,368, 53,270)	0.0 (0.0, 0.0)	-0.7 (-1.0, 8.6)	-1.3 (-2.1, 9.7)	-1.5 (-2.5, 4.4)	-2.2 (-3.6, 1.2)	-2.1 (-3.7, 0.8)	-2.0 (-3.7, 1.2)	-2.1 (-4.6, 2.5)
Haemorrhagic stroke	0 (0, 0)	476 (338, 800)	3,044 (2,245, 4,131)	7,768 (5,842, 10,481)	20,968 (15,582, 28,277)	43,205 (32,197, 57,837)	68,579 (49,720, 95,338)	142,988 (104,336, 199,787)	0.0 (0.0, 0.0)	5.7 (4.1, 9.6)	10.4 (7.7, 14.1)	11.3 (8.5, 15.3)	11.8 (8.8, 16.0)	10.8 (8.0, 14.4)	9.7 (7.0, 13.4)	9.2 (6.7, 12.8)
Cardiomyopathy, myocarditis, endocarditis	0 (0, 0)	89 (72, 356)	763 (734, 1,196)	3,021 (2,998, 3,313)	3,738 (3,712, 4,015)	5,819 (5,778, 6,319)	4,562 (4,498, 5,288)	6,662 (6,568, 7,494)	0.0 (0.0, 0.0)	1.9 (1.5, 7.4)	5.0 (4.8, 7.8)	12.3 (12.2, 13.5)	11.5 (11.4, 12.4)	12.3 (12.2, 13.3)	7.5 (7.4, 8.7)	3.8 (3.8, 4.3)
Digestive diseases	0 (0, 0)	3,697 (2,981, 4,801)	26,027 (21,489, 29,787)	61,967 (52,086, 69,561)	102,541 (87,991, 114,514)	139,846 (122,325, 155,442)	144,097 (125,245, 163,225)	138,180 (119,086, 162,371)	0.0 (0.0, 0.0)	12.8 (10.3, 16.6)	28.4 (23.4, 32.5)	37.8 (31.7, 42.4)	39.9 (34.2, 44.5)	35.6 (31.2, 39.6)	27.9 (24.3, 31.6)	13.9 (11.9, 16.3)

Cause of disease or injury	Alcohol-attributable deaths								Population Attributable Fraction							
	0 to 14	15 to 19	20 to 29	30 to 39	40 to 49	50 to 59	60 to 69	≥70	0 to 14	15 to 19	20 to 29	30 to 39	40 to 49	50 to 59	60 to 69	≥70
Cirrhosis of the liver	0 (0, 0)	3,593 (2,871, 4,703)	24,369 (19,967, 27,922)	58,145 (48,310, 65,466)	97,529 (83,255, 109,043)	134,702 (117,371, 150,135)	139,105 (120,520, 157,774)	130,695 (111,668, 154,038)	0.0 (0.0, 0.0)	28.8 (23.0, 37.7)	49.6 (40.7, 56.9)	54.0 (44.9, 60.8)	55.3 (47.2, 61.9)	52.5 (45.7, 58.5)	47.8 (41.4, 54.2)	38.9 (33.2, 45.8)
Pancreatitis	0 (0, 0)	104 (69, 175)	1,658 (1,138, 2,306)	3,822 (2,802, 5,007)	5,012 (3,614, 6,648)	5,144 (3,823, 6,838)	4,993 (3,648, 6,858)	7,485 (5,207, 10,933)	0.0 (0.0, 0.0)	11.0 (7.3, 18.5)	27.3 (18.7, 38.0)	32.2 (23.6, 42.2)	31.6 (22.8, 41.9)	28.3 (21.0, 37.6)	23.2 (17.0, 31.9)	18.5 (12.9, 27.0)
**Injuries**	33,939 (24,852, 55,874)	38,649 (30,041, 55,306)	175,987 (132,734, 229,571)	155,784 (121,101, 199,599)	134,313 (105,956, 171,523)	118,870 (95,230, 150,613)	93,522 (75,533, 120,815)	111,750 (88,364, 150,409)	5.3 (3.9, 8.7)	12.8 (9.9, 18.3)	21.9 (16.5, 28.5)	24.0 (18.7, 30.8)	25.2 (19.9, 32.2)	23.2 (18.6, 29.4)	18.4 (14.8, 23.7)	12.1 (9.5, 16.2)
Unintentional injuries	33,939 (24,852, 55,874)	28,303 (22,000, 41,673)	116,260 (89,437, 156,872)	101,421 (79,935, 133,907)	94,461 (74,332, 124,706)	87,006 (68,821, 114,591)	73,167 (57,844, 97,747)	94,404 (72,182, 131,147)	5.9 (4.4, 9.8)	16.0 (12.5, 23.6)	26.1 (20.1, 35.3)	27.3 (21.5, 36.0)	27.6 (21.7, 36.5)	24.6 (19.5, 32.4)	19.1 (15.1, 25.5)	12.0 (9.2, 16.7)
Road injury	33,939 (24,852, 55,874)	20,533 (14,439, 32,255)	78,740 (55,955, 114,469)	62,508 (44,670, 90,113)	56,129 (39,962, 81,081)	48,048 (34,254, 70,620)	37,797 (27,049, 56,688)	33,073 (23,851, 51,278)	23.1 (16.9, 38.0)	20.2 (14.2, 31.8)	29.1 (20.7, 42.3)	30.0 (21.4, 43.2)	30.0 (21.3, 43.3)	27.5 (19.6, 40.4)	24.7 (17.7, 37.1)	20.8 (15.0, 32.2)
Poisonings	0 (0, 0)	409 (260, 674)	2,543 (1,618, 3,561)	2,273 (1,509, 3,120)	2,342 (1,549, 3,222)	1,969 (1,333, 2,670)	1,718 (1,148, 2,401)	1,480 (965, 2,178)	0.0 (0.0, 0.0)	10.0 (6.3, 16.4)	20.7 (13.1, 28.9)	22.9 (15.2, 31.4)	21.8 (14.4, 30.0)	19.3 (13.1, 26.1)	13.9 (9.3, 19.4)	9.5 (6.2, 13.9)
Falls	0 (0, 0)	809 (499, 1,292)	5,398 (3,302, 7,581)	7,347 (4,633, 10,187)	9,340 (5,800, 12,989)	11,524 (7,197, 16,252)	12,814 (8,082, 18,589)	32,138 (20,579, 47,964)	0.0 (0.0, 0.0)	10.3 (6.4, 16.5)	22.4 (13.7, 31.5)	24.6 (15.5, 34.1)	25.4 (15.7, 35.3)	20.9 (13.0, 29.4)	13.5 (8.5, 19.6)	8.8 (5.7, 13.2)
Fire, heat and hot substances	0 (0, 0)	531 (352, 852)	2,813 (1,893, 3,963)	3,106 (2,230, 4,165)	2,614 (1,947, 3,375)	2,773 (2,113, 3,543)	2,362 (1,750, 3,116)	2,960 (2,148, 4,126)	0.0 (0.0, 0.0)	6.7 (4.4, 10.8)	14.7 (9.9, 20.7)	17.5 (12.5, 23.4)	20.9 (15.5, 26.9)	20.1 (15.3, 25.7)	15.5 (11.5, 20.5)	10.4 (7.5, 14.5)
Drowning	0 (0, 0)	2,708 (1,552, 4,430)	8,553 (5,129, 12,090)	7,214 (4,754, 9,806)	5,983 (3,958, 8,111)	5,109 (3,388, 6,928)	4,223 (2,749, 5,918)	4,320 (2,754, 6,384)	0.0 (0.0, 0.0)	11.6 (6.6, 19.0)	23.4 (14.1, 33.1)	26.0 (17.1, 35.3)	26.1 (17.3, 35.4)	22.6 (15.0, 30.6)	17.3 (11.2, 24.2)	11.5 (7.3, 16.9)
Exposure to mechanical forces	0 (0, 0)	786 (450, 1,307)	4,655 (2,677, 6,682)	4,573 (2,818, 6,377)	4,114 (2,572, 5,680)	3,443 (2,128, 4,848)	2,311 (1,417, 3,322)	1,635 (1,042, 2,440)	0.0 (0.0, 0.0)	10.8 (6.2, 18.0)	23.0 (13.2, 33.0)	24.9 (15.4, 34.8)	25.8 (16.2, 35.7)	23.1 (14.3, 32.5)	17.7 (10.8, 25.4)	10.5 (6.7, 15.7)
Other unintentional injuries	0 (0, 0)	2,526 (1,491, 4,087)	13,558 (8,169, 19,256)	14,400 (9,248, 19,753)	13,940 (9,215, 18,886)	14,141 (9,936, 18,840)	11,942 (8,185, 16,409)	18,798 (12,273, 27,275)	0.0 (0.0, 0.0)	10.4 (6.1, 16.8)	22.0 (13.3, 31.2)	24.4 (15.6, 33.4)	25.1 (16.6, 34.0)	22.9 (16.1, 30.5)	16.8 (11.5, 23.1)	11.3 (7.4, 16.4)
Intentional injuries	0 (0, 0)	10,346 (4,058, 18,841)	59,726 (24,058, 93,803)	54,363 (25,375, 81,565)	39,851 (19,052, 59,247)	31,863 (16,521, 46,935)	20,355 (10,704, 30,771)	17,346 (9,406, 27,018)	0.0 (0.0, 0.0)	8.2 (3.2, 14.9)	16.6 (6.7, 26.1)	19.7 (9.2, 29.5)	20.8 (10.0, 31.0)	20.0 (10.4, 29.4)	16.2 (8.5, 24.5)	12.3 (6.7, 19.2)

Cause of disease or injury	Alcohol-attributable deaths								Population Attributable Fraction							
	0 to 14	15 to 19	20 to 29	30 to 39	40 to 49	50 to 59	60 to 69	≥70	0 to 14	15 to 19	20 to 29	30 to 39	40 to 49	50 to 59	60 to 69	≥70
Self-harm	0 (0, 0)	4,491 (2,202, 7,716)	29,477 (14,112, 44,586)	31,085 (16,439, 45,298)	26,207 (13,415, 38,313)	23,767 (12,634, 34,727)	16,508 (8,529, 25,050)	15,484 (8,170, 24,265)	0.0 (0.0, 0.0)	8.5 (4.2, 14.6)	18.4 (8.8, 27.9)	23.0 (12.2, 33.6)	23.8 (12.2, 34.7)	21.9 (11.7, 32.1)	17.1 (8.9, 26.0)	12.8 (6.8, 20.1)
Interpersonal violence	0 (0, 0)	5,855 (1,759, 11,248)	30,249 (9,453, 49,754)	23,278 (9,046, 36,561)	13,645 (5,658, 21,148)	8,096 (4,054, 12,334)	3,847 (2,139, 5,741)	1,862 (1,212, 2,740)	0.0 (0.0, 0.0)	11.1 (3.3, 21.2)	20.7 (6.5, 34.1)	22.4 (8.7, 35.1)	22.0 (9.1, 34.1)	20.1 (10.1, 30.7)	16.6 (9.2, 24.7)	11.1 (7.2, 16.4)

Table A5. Global alcohol-attributable premature deaths and cause in 2016 by sex and 95% uncertainty intervals

Cause of disease or injury	Men		Women		Total	
	Alcohol-attributable burden	PAF (%)	Alcohol-attributable burden	PAF (%)	Alcohol-attributable burden	PAF (%)
**All Causes**	1,635,768 (1,354,322, 1,956,578)	9.5 (7.9, 11.3)	461,122 (392,684, 635,474)	3.8 (3.2, 5.3)	2,096,890 (1,803,361, 2,502,738)	7.1 (6.1, 8.5)
**Communicable, maternal, perinatal and nutritional conditions**	209,878 (83,801, 351,133)	4.7 (1.9, 7.8)	49,525 (22,108, 121,246)	1.3 (0.6, 3.1)	259,403 (130,443, 425,336)	3.1 (1.5, 5.0)
Tuberculosis	156,414 (32,512, 288,818)	25.7 (5.3, 47.5)	33,107 (5,521, 91,119)	10.5 (1.7, 28.8)	189,521 (61,939, 346,539)	20.5 (6.7, 37.5)
HIV AIDS	22,667 (13,363, 34,806)	3.9 (2.3, 6.0)	7,468 (4,418, 22,791)	1.8 (1.1, 5.5)	30,135 (20,513, 49,654)	3.0 (2.1, 5.0)
Lower respiratory infections	30,798 (12,066, 53,435)	3.6 (1.4, 6.3)	8,949 (4,273, 19,241)	1.2 (0.6, 2.6)	39,747 (21,549, 65,929)	2.5 (1.4, 4.1)
**Noncommunicable diseases**	806,025 (678,824, 912,381)	8.1 (6.8, 9.2)	280,398 (227,655, 381,105)	4.0 (3.2, 5.4)	1,086,423 (943,421, 1,248,131)	6.4 (5.6, 7.4)
Malignant neoplasms	184,577 (150,737, 219,528)	6.8 (5.6, 8.1)	32,907 (25,564, 43,797)	1.6 (1.2, 2.1)	217,484 (182,111, 257,881)	4.5 (3.8, 5.4)
Lip and oral cavity cancer	32,438 (26,064, 37,412)	39.5 (31.7, 45.6)	5,653 (4,110, 7,629)	16.6 (12.1, 22.4)	38,090 (30,884, 44,183)	32.8 (26.6, 38.1)
Other pharynx cancers	26,319 (21,521, 30,111)	41.5 (33.9, 47.5)	2,350 (1,754, 3,178)	17.2 (12.8, 23.2)	28,669 (23,608, 32,836)	37.2 (30.6, 42.6)
Oesophagus cancer	43,934 (36,068, 50,885)	25.1 (20.6, 29.1)	4,943 (3,701, 6,819)	8.9 (6.7, 12.3)	48,876 (40,484, 56,852)	21.2 (17.6, 24.7)
Colon and rectum cancers	34,323 (27,259, 41,463)	17.0 (13.5, 20.5)	7,371 (4,363, 11,364)	5.1 (3.0, 7.9)	41,694 (34,150, 50,205)	12.0 (9.8, 14.5)
Liver cancer	47,564 (23,020, 74,494)	12.7 (6.1, 19.9)	12,591 (6,551, 21,210)	10.3 (5.4, 17.4)	60,155 (34,808, 90,079)	12.1 (7.0, 18.1)
Breast cancer	0 (0, 0)	0.0 (0.0, 0.0)	29,607 (23,961, 37,883)	7.5 (6.1, 9.6)	29,607 (23,961, 37,883)	7.5 (6.1, 9.6)
Larynx cancer	12,588 (9,560, 15,684)	25.2 (19.2, 31.4)	656 (445, 981)	10.5 (7.1, 15.7)	13,243 (10,195, 16,413)	23.6 (18.2, 29.2)
Diabetes mellitus	6,551 (539, 15,315)	1.9 (0.2, 4.4)	-21,882 (-30,508, -10,345)	-6.3 (-8.8, -3.0)	-15,331 (-25,309, -119)	-2.2 (-3.6, 0.0)
Alcohol use disorders	108,529 (108,529, 108,529)	100.0 (100.0, 100.0)	19,232 (19,232, 19,232)	100.0 (100.0, 100.0)	127,761 (127,761, 127,761)	100.0 (100.0, 100.0)
Epilepsy	10,734 (7,948, 13,537)	16.1 (11.9, 20.3)	2,929 (2,199, 4,243)	6.2 (4.7, 9.0)	13,663 (10,746, 17,056)	12.0 (9.5, 15.0)
Cardiovascular diseases	127,820 (43,027, 195,075)	3.2 (1.1, 5.0)	94,000 (45,019, 178,680)	3.8 (1.8, 7.1)	221,820 (126,584, 329,425)	3.4 (2.0, 5.1)
Hypertensive heart disease	20,557 (15,461, 25,183)	14.3 (10.8, 17.5)	4,330 (2,054, 9,906)	3.1 (1.5, 7.2)	24,887 (19,428, 32,308)	8.8 (6.9, 11.4)
Ischaemic heart disease	9,590 (-69,844, 62,507)	0.4 (-3.2, 2.8)	39,513 (-10,105, 99,599)	3.4 (-0.9, 8.5)	49,102 (-40,826, 127,464)	1.5 (-1.2, 3.8)
Ischaemic stroke	-4,240 (-13,316, 8,270)	-1.0 (-3.3, 2.0)	-9,961 (-17,797, 6,083)	-3.4 (-6.0, 2.1)	-14,202 (-25,829, 8,909)	-2.0 (-3.7, 1.3)
Haemorrhagic stroke	86,592 (57,277, 118,882)	10.8 (7.1, 14.8)	57,449 (34,253, 96,327)	9.5 (5.7, 15.9)	144,040 (105,923, 196,865)	10.2 (7.5, 14.0)
Cardiomyopathy, myocarditis, endocarditis	15,322 (15,120, 17,812)	11.8 (11.7, 13.7)	2,670 (2,670, 2,682)	3.9 (3.9, 3.9)	17,992 (17,792, 20,486)	9.1 (9.0, 10.3)
Digestive diseases	355,227 (292,762, 405,956)	35.4 (29.2, 40.5)	122,949 (105,226, 143,442)	23.2 (19.9, 27.1)	478,175 (412,119, 537,331)	31.2 (26.9, 35.1)
Cirrhosis of the liver	337,241 (276,426, 387,254)	51.9 (42.6, 59.7)	120,202 (102,633, 139,416)	44.7 (38.2, 51.9)	457,444 (392,295, 515,043)	49.8 (42.7, 56.1)
Pancreatitis	17,986 (12,251, 23,543)	35.5 (24.2, 46.4)	2,746 (1,021, 6,569)	11.3 (4.2, 27.0)	20,732 (15,094, 27,831)	27.6 (20.1, 37.1)
**Injuries**	619,864 (462,629, 823,819)	22.2 (16.6, 29.6)	131,199 (103,828, 183,702)	11.2 (8.9, 15.7)	751,063 (585,447, 983,302)	19.0 (14.8, 24.9)
Unintentional injuries	435,918 (326,614, 594,860)	23.6 (17.6, 32.1)	98,640 (80,760, 140,103)	12.5 (10.2, 17.7)	534,558 (417,221, 725,369)	20.2 (15.8, 27.4)
Road injury	269,871 (184,126, 402,578)	28.8 (19.7, 43.0)	67,822 (53,076, 101,014)	22.1 (17.3, 32.9)	337,693 (241,180, 501,100)	27.2 (19.4, 40.3)
Poisonings	8,618 (4,930, 12,599)	16.3 (9.3, 23.9)	2,637 (1,448, 4,375)	6.9 (3.8, 11.4)	11,255 (7,416, 15,647)	12.4 (8.1, 17.2)
Falls	40,750 (23,106, 59,762)	20.3 (11.5, 29.7)	6,482 (3,767, 10,716)	6.7 (3.9, 11.1)	47,232 (29,513, 66,890)	15.9 (9.9, 22.5)
Fire, heat and hot substances	9,863 (6,367, 13,614)	17.4 (11.2, 24.0)	4,337 (2,607, 7,026)	6.4 (3.9, 10.4)	14,200 (10,284, 19,014)	11.4 (8.3, 15.3)
Drowning	29,195 (17,120, 42,271)	14.9 (8.7, 21.6)	4,595 (2,628, 7,489)	5.2 (3.0, 8.5)	33,789 (21,529, 47,283)	11.9 (7.6, 16.6)
Exposure to mechanical forces	17,703 (9,939, 25,854)	18.2 (10.2, 26.6)	2,179 (1,229, 3,582)	5.8 (3.3, 9.6)	19,881 (12,061, 28,215)	14.8 (9.0, 21.0)
Other unintentional injuries	59,918 (35,857, 85,688)	19.3 (11.5, 27.6)	10,589 (6,752, 16,532)	6.8 (4.3, 10.6)	70,507 (46,245, 97,232)	15.1 (9.9, 20.8)
Intentional injuries	183,947 (65,800, 294,341)	19.7 (7.0, 31.5)	32,559 (14,213, 57,065)	8.6 (3.8, 15.1)	216,506 (99,768, 331,161)	16.5 (7.6, 25.2)
Self-harm	107,742 (43,690, 167,084)	24.8 (10.1, 38.5)	23,793 (9,576, 42,442)	10.0 (4.0, 17.8)	131,535 (67,331, 195,691)	19.6 (10.0, 29.1)
Interpersonal violence	76,204 (21,765, 127,416)	20.5 (5.9, 34.3)	8,766 (4,544, 14,634)	9.9 (5.1, 16.6)	84,970 (32,109, 136,785)	18.5 (7.0, 29.7)

PAF: Population Attributable Fraction

Table A6. Global alcohol-attributable years of life lost (YLL) by age and cause in 2016 among men and 95% uncertainty intervals

Cause of disease or injury	Alcohol-attributable YLL								Population attributable fraction (%)							
	0 to 14	15 to 19	20 to 29	30 to 39	40 to 49	50 to 59	60 to 69	≥70	0 to 14	15 to 19	20 to 29	30 to 39	40 to 49	50 to 59	60 to 69	≥70
**All Causes**	1,720,120 (1,257,595, 2,838,421)	2,644,024 (2,021,911, 3,857,198)	13,503,637 (10,469,545, 17,151,403)	14,756,450 (12,121,924, 17,659,686)	14,908,227 (12,515,804, 17,373,212)	14,882,339 (12,783,815, 17,130,296)	11,403,525 (9,472,871, 13,453,171)	7,643,206 (5,393,308, 10,173,746)	0.6 (0.4, 0.9)	8.9 (6.8, 13.0)	17.3 (13.4, 22.0)	17.7 (14.5, 21.2)	15.5 (13.0, 18.1)	11.7 (10.1, 13.5)	7.6 (6.3, 9.0)	4.2 (3.0, 5.6)
**Communicable, maternal, perinatal and nutritional conditions**	0 (0, 0)	138,127 (58,681, 357,792)	1,481,380 (517,955, 2,513,384)	2,244,958 (951,677, 3,551,078)	2,293,051 (958,772, 3,666,905)	1,918,092 (747,099, 3,224,465)	1,314,171 (515,504, 2,320,432)	1,133,752 (477,803, 2,008,801)	0.0 (0.0, 0.0)	2.1 (0.9, 5.5)	9.3 (3.3, 15.9)	10.5 (4.5, 16.6)	11.7 (4.9, 18.7)	12.3 (4.8, 20.7)	9.8 (3.8, 17.2)	6.6 (2.8, 11.7)
Tuberculosis	0 (0, 0)	104,018 (21,327, 273,191)	1,187,961 (247,852, 2,189,478)	1,603,609 (346,460, 2,836,326)	1,679,016 (349,878, 2,990,266)	1,477,452 (313,725, 2,707,065)	961,271 (188,378, 1,882,026)	612,803 (110,212, 1,276,270)	0.0 (0.0, 0.0)	10.3 (2.1, 27.1)	27.0 (5.6, 49.7)	30.1 (6.5, 53.2)	30.3 (6.3, 53.9)	27.1 (5.7, 49.6)	22.7 (4.5, 44.5)	17.6 (3.2, 36.7)
HIV AIDS	0 (0, 0)	11,299 (6,815, 62,605)	139,753 (81,606, 216,252)	431,542 (251,611, 654,288)	380,645 (226,445, 571,028)	139,388 (82,742, 208,095)	31,506 (18,561, 47,237)	4,876 (2,677, 7,669)	0.0 (0.0, 0.0)	1.0 (0.6, 5.6)	3.8 (2.2, 5.8)	4.4 (2.6, 6.7)	4.7 (2.8, 7.1)	4.4 (2.6, 6.5)	4.0 (2.4, 6.0)	3.0 (1.6, 4.7)
Lower respiratory infections	0 (0, 0)	22,810 (6,561, 66,038)	153,666 (48,701, 279,333)	209,808 (98,122, 339,020)	233,390 (102,468, 384,730)	301,252 (130,177, 499,262)	321,394 (108,931, 582,242)	516,073 (133,819, 1,005,582)	0.0 (0.0, 0.0)	2.5 (0.7, 7.3)	7.7 (2.4, 13.9)	10.5 (4.9, 16.9)	10.5 (4.6, 17.3)	9.6 (4.2, 15.9)	7.4 (2.5, 13.5)	6.1 (1.6, 11.8)
**Noncommunicable diseases**	0 (0, 0)	256,488 (212,787, 350,810)	1,976,517 (1,668,650, 2,222,033)	4,848,778 (4,172,081, 5,329,387)	7,293,188 (6,298,076, 7,981,782)	9,252,941 (7,977,862, 10,239,928)	7,962,972 (6,454,242, 9,370,713)	5,324,958 (3,296,331, 7,531,279)	0.0 (0.0, 0.0)	3.4 (2.9, 4.7)	9.3 (7.9, 10.5)	14.5 (12.5, 15.9)	12.7 (11.0, 13.9)	9.5 (8.2, 10.5)	6.3 (5.1, 7.4)	3.4 (2.1, 4.8)
Malignant neoplasms	0 (0, 0)	0 (0, 0)	55,391 (41,255, 80,878)	430,216 (328,886, 535,681)	1,258,188 (992,667, 1,539,267)	2,513,842 (2,088,462, 2,948,148)	2,616,697 (2,192,573, 3,046,997)	1,932,408 (1,613,291, 2,310,857)	0.0 (0.0, 0.0)	0.0 (0.0, 0.0)	1.3 (1.0, 1.9)	6.4 (4.9, 7.9)	8.1 (6.4, 9.9)	8.5 (7.0, 9.9)	7.2 (6.0, 8.4)	5.4 (4.5, 6.4)
Lip and oral cavity cancer	0 (0, 0)	0 (0, 0)	11,887 (6,925, 18,548)	122,686 (85,250, 150,775)	263,810 (201,459, 309,454)	435,214 (355,619, 494,329)	356,670 (295,659, 407,667)	172,736 (144,635, 198,894)	0.0 (0.0, 0.0)	0.0 (0.0, 0.0)	8.4 (4.9, 13.1)	34.8 (24.2, 42.7)	38.3 (29.2, 44.9)	42.1 (34.4, 47.9)	41.6 (34.5, 47.6)	37.3 (31.2, 42.9)
Other pharynx cancers	0 (0, 0)	0 (0, 0)	2,984 (1,866, 4,581)	45,810 (33,164, 55,759)	177,820 (137,880, 207,605)	378,514 (312,399, 428,475)	320,220 (266,485, 365,285)	146,894 (120,860, 170,973)	0.0 (0.0, 0.0)	0.0 (0.0, 0.0)	8.5 (5.3, 13.0)	33.7 (24.4, 41.0)	39.0 (30.2, 45.5)	43.6 (36.0, 49.4)	42.3 (35.2, 48.3)	34.9 (28.7, 40.7)
Oesophagus cancer	0 (0, 0)	0 (0, 0)	2,382 (1,695, 3,541)	30,024 (24,546, 35,842)	180,987 (151,677, 209,142)	553,464 (460,305, 634,840)	680,753 (552,564, 792,775)	464,019 (366,575, 561,539)	0.0 (0.0, 0.0)	0.0 (0.0, 0.0)	4.4 (3.1, 6.6)	15.8 (12.9, 18.9)	22.6 (19.0, 26.2)	25.9 (21.5, 29.7)	25.6 (20.8, 29.8)	22.5 (17.8, 27.2)
Colon and rectum cancers	0 (0, 0)	0 (0, 0)	13,095 (10,996, 15,913)	76,058 (60,331, 92,857)	177,792 (140,177, 215,975)	377,640 (299,040, 458,552)	519,247 (413,431, 624,349)	603,843 (489,643, 723,395)	0.0 (0.0, 0.0)	0.0 (0.0, 0.0)	5.7 (4.8, 7.0)	13.9 (11.0, 17.0)	15.4 (12.2, 18.7)	17.2 (13.6, 20.8)	18.0 (14.3, 21.6)	18.1 (14.7, 21.7)
Liver cancer	0 (0, 0)	0 (0, 0)	24,371 (12,361, 45,393)	145,228 (67,904, 228,519)	395,516 (183,040, 628,666)	601,629 (291,630, 938,227)	558,238 (275,565, 868,761)	435,489 (216,258, 704,676)	0.0 (0.0, 0.0)	0.0 (0.0, 0.0)	5.3 (2.7, 9.8)	11.8 (5.5, 18.5)	12.3 (5.7, 19.5)	13.0 (6.3, 20.3)	13.3 (6.6, 20.7)	13.2 (6.6, 21.4)
Breast cancer	0 (0, 0)	0 (0, 0)	0 (0, 0)	0 (0, 0)	0 (0, 0)	0 (0, 0)	0 (0, 0)	0 (0, 0)	0.0 (0.0, 0.0)	0.0 (0.0, 0.0)	0.0 (0.0, 1.4)	0.0 (0.0, 0.0)	0.0 (0.0, 0.0)	0.0 (0.0, 0.0)	0.0 (0.0, 0.0)	0.0 (0.0, 0.0)
Larynx cancer	0 (0, 0)	0 (0, 0)	671 (419, 1,068)	10,409 (7,497, 13,390)	62,263 (46,160, 78,791)	167,381 (128,201, 207,594)	181,570 (137,923, 225,984)	109,426 (81,443, 138,940)	0.0 (0.0, 0.0)	0.0 (0.0, 0.0)	4.6 (2.9, 7.3)	20.2 (14.6, 26.0)	23.0 (17.1, 29.2)	26.0 (19.9, 32.3)	25.7 (19.5, 32.0)	22.6 (16.8, 28.7)
Diabetes mellitus	0 (0, 0)	1,439 (-104, 6,431)	7,709 (1,055, 17,887)	15,031 (1,885, 36,065)	36,643 (4,163, 87,059)	73,125 (7,217, 169,299)	94,118 (6,115, 218,597)	105,354 (1,808, 247,385)	0.0 (0.0, 0.0)	1.1 (-0.1, 5.1)	1.8 (0.2, 4.1)	1.9 (0.2, 4.6)	1.9 (0.2, 4.6)	1.9 (0.2, 4.4)	1.9 (0.1, 4.3)	1.9 (0.0, 4.3)

Cause of disease or injury	Alcohol-attributable YLL								Population attributable fraction (%)							
	0 to 14	15 to 19	20 to 29	30 to 39	40 to 49	50 to 59	60 to 69	≥70	0 to 14	15 to 19	20 to 29	30 to 39	40 to 49	50 to 59	60 to 69	≥70
Alcohol use disorders	0 (0, 0)	56,596 (56,596, 56,596)	440,103 (440,103, 440,103)	1,056,150 (1,056,150, 1,056,150)	1,268,411 (1,268,411, 1,268,411)	1,213,443 (1,213,443, 1,213,443)	659,314 (659,314, 659,314)	214,977 (214,977, 214,977)	100.0 (100.0, 100.0)	100.0 (100.0, 100.0)	100.0 (100.0, 100.0)	100.0 (100.0, 100.0)	100.0 (100.0, 100.0)	100.0 (100.0, 100.0)	100.0 (100.0, 100.0)	100.0 (100.0, 100.0)
Epilepsy	0 (0, 0)	38,189 (26,409, 61,885)	166,473 (121,257, 209,359)	136,019 (100,867, 168,608)	101,608 (76,415, 124,338)	65,004 (48,833, 80,745)	41,594 (31,023, 52,139)	27,791 (20,807, 34,969)	0.0 (0.0, 0.0)	6.8 (4.7, 11.1)	18.9 (13.7, 23.7)	22.0 (16.3, 27.2)	23.5 (17.7, 28.8)	23.1 (17.3, 28.6)	21.1 (15.7, 26.4)	18.5 (13.8, 23.2)
Cardiovascular diseases	0 (0, 0)	23,378 (16,102, 53,034)	166,466 (63,737, 285,093)	463,443 (218,723, 664,688)	827,819 (347,819, 1,115,051)	1,452,874 (643,418, 2,030,149)	1,678,427 (397,569, 2,756,178)	1,672,241 (-310,131, 3,576,618)	0.0 (0.0, 0.0)	1.6 (1.1, 3.5)	3.0 (1.1, 5.1)	4.0 (1.9, 5.8)	3.7 (1.5, 5.0)	3.6 (1.6, 5.0)	3.0 (0.7, 4.9)	2.3 (-0.4, 5.0)
Hypertensive heart disease	0 (0, 0)	1,995 (1,372, 3,153)	23,622 (17,377, 28,756)	57,137 (43,012, 68,849)	119,912 (91,503, 144,692)	214,587 (164,702, 261,414)	300,862 (223,633, 370,864)	440,295 (317,615, 554,634)	0.0 (0.0, 0.0)	4.5 (3.1, 7.1)	12.7 (9.4, 15.5)	14.8 (11.1, 17.8)	15.5 (11.8, 18.7)	14.9 (11.4, 18.2)	14.0 (10.4, 17.3)	13.1 (9.5, 16.5)
Ischaemic heart disease	0 (0, 0)	339 (-6,047, 10,215)	-17,535 (-111,597, 78,874)	-86 (-224,027, 160,525)	5,788 (-419,109, 201,609)	115,464 (-674,416, 512,765)	185,118 (-1,011,054, 1,082,736)	204,552 (-1,677,713, 2,011,852)	0.0 (0.0, 0.0)	0.1 (-1.3, 2.3)	-0.7 (-4.7, 3.3)	0.0 (-3.5, 2.5)	0.0 (-3.1, 1.5)	0.5 (-2.8, 2.1)	0.6 (-3.3, 3.5)	0.5 (-4.4, 5.3)
Ischaemic stroke	0 (0, 0)	-450 (-759, 999)	-2,569 (-5,409, 6,152)	-3,143 (-9,694, 10,545)	-10,177 (-29,738, 21,721)	-21,459 (-81,345, 68,263)	-90,525 (-277,301, 156,817)	-186,801 (-547,686, 255,042)	0.0 (0.0, 0.0)	-0.9 (-1.5, 2.0)	-1.2 (-2.6, 3.0)	-0.8 (-2.6, 2.8)	-1.0 (-3.0, 2.2)	-0.7 (-2.7, 2.3)	-1.1 (-3.5, 2.0)	-1.4 (-4.1, 1.9)
Haemorrhagic stroke	0 (0, 0)	15,573 (9,624, 28,885)	119,227 (77,652, 163,177)	259,529 (172,389, 348,335)	558,446 (369,869, 749,711)	959,892 (647,384, 1,297,250)	1,176,011 (770,048, 1,637,367)	1,158,889 (740,603, 1,652,199)	0.0 (0.0, 0.0)	4.9 (3.0, 9.0)	9.9 (6.5, 13.6)	11.3 (7.5, 15.2)	11.8 (7.8, 15.8)	11.2 (7.5, 15.1)	10.6 (7.0, 14.8)	10.2 (6.5, 14.5)
Cardiomyopathy, myocarditis, endocarditis	0 (0, 0)	5,920 (4,698, 25,833)	43,721 (41,758, 72,821)	150,006 (148,682, 166,795)	153,849 (152,610, 166,939)	184,391 (182,840, 202,695)	106,961 (105,177, 126,739)	55,306 (53,871, 68,629)	0.0 (0.0, 0.0)	2.7 (2.1, 11.6)	6.9 (6.6, 11.4)	15.7 (15.6, 17.5)	14.6 (14.4, 15.8)	15.1 (15.0, 16.6)	9.9 (9.8, 11.8)	5.0 (4.9, 6.2)
Digestive diseases	0 (0, 0)	136,885 (97,300, 202,157)	1,140,375 (887,323, 1,334,432)	2,747,920 (2,213,815, 3,142,998)	3,800,519 (3,145,445, 4,305,043)	3,934,653 (3,283,705, 4,453,720)	2,872,821 (2,384,438, 3,301,783)	1,372,186 (1,107,170, 1,655,754)	0.0 (0.0, 0.0)	12.6 (9.0, 18.6)	30.9 (24.0, 36.2)	41.3 (33.3, 47.2)	43.1 (35.6, 48.8)	39.4 (32.8, 44.5)	32.3 (26.8, 37.1)	19.2 (15.5, 23.1)
Cirrhosis of the liver	0 (0, 0)	131,105 (91,197, 195,864)	1,043,537 (800,962, 1,232,513)	2,551,888 (2,034,172, 2,934,807)	3,588,782 (2,941,875, 4,079,575)	3,770,671 (3,148,755, 4,290,790)	2,756,796 (2,275,575, 3,181,936)	1,288,607 (1,027,984, 1,565,605)	0.0 (0.0, 0.0)	26.0 (18.1, 38.9)	50.2 (38.5, 59.3)	54.8 (43.7, 63.1)	56.1 (46.0, 63.7)	53.9 (45.0, 61.3)	50.7 (41.8, 58.5)	42.7 (34.0, 51.8)
Pancreatitis	0 (0, 0)	5,781 (3,674, 8,959)	96,838 (62,732, 131,185)	196,032 (136,562, 254,861)	211,737 (142,141, 276,043)	163,982 (114,292, 211,118)	116,025 (78,374, 153,456)	83,579 (54,133, 113,358)	0.0 (0.0, 0.0)	14.6 (9.3, 22.7)	32.7 (21.2, 44.3)	37.4 (26.1, 48.7)	37.9 (25.4, 49.4)	36.7 (25.6, 47.3)	33.5 (22.6, 44.2)	30.3 (19.6, 41.1)
**Injuries**	1,720,120 (1,257,595, 2,838,421)	2,249,409 (1,614,575, 3,325,347)	10,045,740 (7,216,269, 13,414,526)	7,662,714 (5,736,516, 9,983,613)	5,321,989 (4,031,974, 6,904,277)	3,711,305 (2,841,353, 4,782,682)	2,126,382 (1,637,457, 2,798,248)	1,184,497 (875,819, 1,645,518)	5.3 (3.9, 8.7)	14.3 (10.3, 21.1)	24.5 (17.6, 32.7)	26.8 (20.1, 35.0)	28.2 (21.4, 36.6)	27.0 (20.7, 34.8)	23.3 (18.0, 30.7)	16.8 (12.4, 23.3)
Unintentional injuries	1,720,120 (1,257,595, 2,838,421)	1,635,276 (1,213,427, 2,462,392)	6,635,716 (4,902,893, 9,029,797)	4,957,725 (3,759,354, 6,613,212)	3,719,715 (2,819,600, 4,943,476)	2,693,361 (2,037,153, 3,578,531)	1,645,188 (1,219,275, 2,230,358)	971,709 (681,986, 1,397,590)	5.9 (4.3, 9.8)	17.2 (12.7, 25.9)	28.5 (21.0, 38.8)	30.2 (22.9, 40.2)	30.5 (23.1, 40.6)	28.4 (21.5, 37.8)	24.1 (17.9, 32.7)	17.0 (12.0, 24.5)

Cause of disease or injury	Alcohol-attributable YLL								Population attributable fraction (%)							
	0 to 14	15 to 19	20 to 29	30 to 39	40 to 49	50 to 59	60 to 69	≥70	0 to 14	15 to 19	20 to 29	30 to 39	40 to 49	50 to 59	60 to 69	≥70
Road injury	1,720,120 (1,257,595, 2,838,421)	1,184,015 (780,241, 1,902,927)	4,523,324 (3,074,769, 6,643,881)	3,056,103 (2,101,557, 4,445,989)	2,177,914 (1,480,128, 3,186,244)	1,448,109 (973,393, 2,138,892)	810,261 (545,869, 1,218,102)	364,051 (247,085, 568,197)	22.8 (16.7, 37.6)	20.4 (13.5, 32.8)	30.2 (20.5, 44.4)	31.6 (21.7, 45.9)	31.9 (21.7, 46.7)	29.9 (20.1, 44.2)	27.4 (18.5, 41.2)	22.4 (15.2, 35.0)
Poisonings	0 (0, 0)	20,256 (9,930, 35,489)	129,673 (68,968, 193,012)	101,255 (58,584, 145,032)	84,027 (48,490, 121,150)	58,327 (35,425, 83,018)	37,919 (22,350, 55,875)	17,438 (9,376, 27,875)	0.0 (0.0, 0.0)	11.4 (5.6, 20.0)	25.1 (13.3, 37.3)	27.7 (16.0, 39.7)	27.3 (15.7, 39.3)	26.1 (15.8, 37.1)	21.2 (12.5, 31.2)	13.5 (7.3, 21.6)
Falls	0 (0, 0)	45,168 (23,223, 78,590)	307,226 (167,966, 449,315)	371,011 (215,029, 528,359)	389,173 (224,496, 557,364)	375,918 (215,959, 548,663)	303,752 (169,251, 458,043)	307,498 (161,669, 495,688)	0.0 (0.0, 0.0)	11.8 (6.1, 20.6)	25.5 (13.9, 37.3)	27.3 (15.8, 38.9)	28.1 (16.2, 40.2)	25.5 (14.7, 37.3)	20.0 (11.2, 30.2)	13.9 (7.3, 22.3)
Fire, heat and hot substances	0 (0, 0)	17,030 (8,601, 30,593)	109,565 (60,693, 162,136)	119,948 (75,963, 165,750)	90,822 (61,018, 120,899)	80,571 (56,184, 105,816)	51,666 (34,732, 70,476)	29,230 (17,642, 43,278)	0.0 (0.0, 0.0)	10.4 (5.2, 18.6)	22.9 (12.7, 33.8)	26.8 (17.0, 37.1)	29.9 (20.1, 39.7)	29.4 (20.5, 38.6)	24.5 (16.4, 33.4)	16.4 (9.9, 24.2)
Drowning	0 (0, 0)	169,736 (85,129, 289,539)	506,240 (277,982, 743,228)	362,273 (222,703, 506,772)	242,959 (148,484, 339,609)	165,166 (102,811, 232,513)	100,782 (59,286, 145,926)	50,017 (27,082, 76,914)	0.0 (0.0, 0.0)	12.8 (6.4, 21.8)	26.0 (14.3, 38.2)	29.3 (18.0, 41.0)	29.5 (18.0, 41.2)	27.2 (16.9, 38.3)	23.0 (13.5, 33.4)	16.8 (9.1, 25.8)
Exposure to mechanical forces	0 (0, 0)	49,594 (24,861, 85,463)	282,176 (149,976, 417,642)	235,223 (134,780, 336,388)	170,242 (98,790, 243,977)	115,718 (66,906, 166,348)	58,128 (32,557, 85,828)	20,619 (11,275, 31,996)	0.0 (0.0, 0.0)	12.1 (6.0, 20.8)	25.1 (13.3, 37.2)	27.6 (15.8, 39.5)	29.0 (16.9, 41.6)	27.0 (15.6, 38.8)	23.1 (12.9, 34.1)	15.0 (8.2, 23.3)
Other unintentional injuries	0 (0, 0)	149,476 (74,311, 258,198)	777,513 (416,100, 1,155,280)	711,911 (424,303, 1,011,203)	564,578 (345,606, 790,731)	449,551 (290,618, 616,634)	282,680 (175,660, 401,685)	182,856 (99,795, 281,015)	0.0 (0.0, 0.0)	11.9 (5.9, 20.5)	25.5 (13.7, 37.9)	28.5 (17.0, 40.5)	29.0 (17.8, 40.6)	27.8 (17.9, 38.1)	22.5 (14.0, 32.0)	16.4 (9.0, 25.2)
Intentional injuries	0 (0, 0)	614,133 (134,721, 1,209,097)	3,410,025 (974,972, 5,636,292)	2,704,989 (1,026,707, 4,225,990)	1,602,274 (601,047, 2,487,083)	1,017,944 (442,915, 1,556,177)	481,195 (200,751, 759,774)	212,788 (87,239, 359,707)	0.0 (0.0, 0.0)	9.9 (2.2, 19.5)	19.3 (5.5, 31.8)	22.3 (8.5, 34.9)	23.9 (9.0, 37.1)	23.9 (10.4, 36.5)	20.9 (8.7, 33.0)	15.7 (6.4, 26.6)
Self-harm	0 (0, 0)	223,962 (57,603, 423,646)	1,549,035 (543,215, 2,481,380)	1,497,803 (657,153, 2,261,693)	1,028,113 (417,016, 1,556,979)	752,959 (333,366, 1,135,193)	389,694 (159,944, 618,230)	191,212 (77,082, 322,790)	0.0 (0.0, 0.0)	11.7 (3.0, 22.1)	23.9 (8.4, 38.3)	27.6 (12.1, 41.7)	28.3 (11.5, 42.8)	27.2 (12.0, 41.0)	22.8 (9.3, 36.1)	16.7 (6.7, 28.2)
Interpersonal violence	0 (0, 0)	390,171 (78,173, 782,855)	1,860,990 (425,148, 3,155,446)	1,207,186 (359,817, 1,969,616)	574,162 (180,917, 930,337)	264,985 (108,043, 419,895)	91,501 (41,087, 144,597)	21,575 (10,123, 36,176)	0.0 (0.0, 0.0)	11.9 (2.4, 23.9)	22.3 (5.1, 37.9)	24.2 (7.2, 39.5)	23.9 (7.5, 38.7)	21.9 (8.9, 34.8)	19.1 (8.6, 30.2)	12.4 (5.8, 20.8)

Table A7. Global alcohol-attributable years of life lost (YLL) by age and cause in 2016 among women and 95% uncertainty intervals

Cause of disease or injury	Alcohol-attributable YLL								Population Attributable Fraction							
	0 to 14	15 to 19	20 to 29	30 to 39	40 to 49	50 to 59	60 to 69	≥70	0 to 14	15 to 19	20 to 29	30 to 39	40 to 49	50 to 59	60 to 69	≥70
**All Causes**	1,183,488 (868,328, 1,944,651)	913,204 (778,992, 1,354,115)	3,118,517 (2,594,273, 4,234,522)	3,361,456 (2,951,155, 4,508,630)	3,770,391 (3,366,000, 4,902,342)	4,166,028 (3,676,889, 5,560,159)	3,866,246 (3,137,969, 5,571,895)	4,754,634 (2,992,335, 7,508,328)	0.4 (0.3, 0.7)	4.2 (3.6, 6.2)	6.2 (5.2, 8.5)	6.4 (5.6, 8.6)	6.4 (5.7, 8.3)	5.3 (4.7, 7.1)	3.7 (3.0, 5.3)	2.6 (1.6, 4.1)
**Communicable, maternal, perinatal and nutritional conditions**	0 (0, 0)	90,918 (36,867, 290,917)	545,541 (207,288, 1,260,780)	629,702 (312,466, 1,472,727)	486,890 (233,042, 1,150,663)	351,106 (150,951, 901,780)	267,100 (115,166, 682,615)	296,448 (136,884, 703,161)	0.0 (0.0, 0.0)	1.1 (0.4, 3.4)	2.5 (1.0, 5.8)	3.0 (1.5, 7.0)	3.7 (1.8, 8.6)	3.8 (1.6, 9.7)	2.6 (1.1, 6.7)	1.7 (0.8, 4.0)
Tuberculosis	0 (0, 0)	67,361 (11,070, 221,826)	392,235 (55,873, 1,008,703)	390,579 (67,324, 1,009,308)	315,086 (54,989, 825,805)	247,350 (43,229, 708,025)	176,526 (29,463, 535,327)	120,551 (19,703, 398,149)	0.0 (0.0, 0.0)	6.6 (1.1, 21.8)	14.0 (2.0, 36.1)	14.1 (2.4, 36.6)	13.7 (2.4, 36.0)	10.4 (1.8, 29.9)	8.2 (1.4, 24.8)	5.3 (0.9, 17.7)
HIV AIDS	0 (0, 0)	8,736 (5,377, 54,687)	87,506 (51,004, 210,175)	166,368 (97,333, 481,505)	105,762 (62,731, 325,116)	26,323 (16,340, 100,128)	5,730 (3,496, 27,102)	643 (386, 6,757)	0.0 (0.0, 0.0)	0.9 (0.6, 5.7)	2.3 (1.3, 5.4)	2.2 (1.3, 6.5)	2.2 (1.3, 6.8)	1.6 (1.0, 6.2)	1.4 (0.9, 6.6)	0.8 (0.5, 8.2)
Lower respiratory infections	0 (0, 0)	14,822 (5,375, 56,254)	65,801 (26,710, 139,173)	72,755 (40,362, 143,788)	66,042 (35,336, 130,385)	77,434 (38,906, 161,138)	84,843 (36,780, 189,913)	175,255 (65,111, 382,312)	0.0 (0.0, 0.0)	1.8 (0.6, 6.7)	4.3 (1.7, 9.1)	5.4 (3.0, 10.6)	5.1 (2.7, 10.0)	3.8 (1.9, 8.0)	2.5 (1.1, 5.6)	2.0 (0.7, 4.3)
**Noncommunicable diseases**	0 (0, 0)	190,579 (153,662, 255,041)	831,289 (696,746, 1,037,576)	1,459,239 (1,277,608, 1,798,259)	2,252,836 (1,930,550, 2,866,697)	3,068,072 (2,570,983, 4,049,462)	3,056,673 (2,315,397, 4,480,176)	4,043,412 (2,299,378, 6,581,919)	0.0 (0.0, 0.0)	2.9 (2.4, 3.9)	5.4 (4.5, 6.8)	6.4 (5.6, 7.8)	5.7 (4.9, 7.3)	4.8 (4.0, 6.4)	3.4 (2.6, 5.0)	2.5 (1.4, 4.1)
Malignant neoplasms	0 (0, 0)	0 (0, 0)	27,671 (22,193, 41,062)	268,136 (220,862, 346,697)	550,886 (464,872, 696,119)	794,378 (685,350, 986,627)	692,441 (590,042, 866,998)	625,939 (502,358, 814,291)	0.0 (0.0, 0.0)	0.0 (0.0, 0.0)	0.8 (0.6, 1.1)	3.2 (2.6, 4.1)	3.4 (2.9, 4.3)	3.4 (2.9, 4.2)	2.9 (2.5, 3.7)	2.5 (2.0, 3.3)
Lip and oral cavity cancer	0 (0, 0)	0 (0, 0)	2,552 (1,694, 3,878)	20,043 (14,421, 26,688)	48,654 (34,288, 65,564)	74,266 (54,480, 99,366)	61,673 (45,260, 83,940)	44,024 (32,574, 61,031)	0.0 (0.0, 0.0)	0.0 (0.0, 0.0)	5.5 (3.6, 8.3)	16.6 (11.9, 22.1)	17.3 (12.2, 23.3)	18.2 (13.3, 24.3)	16.1 (11.8, 21.9)	15.1 (11.2, 20.9)
Other pharynx cancers	0 (0, 0)	0 (0, 0)	1,079 (697, 1,757)	8,625 (5,802, 11,799)	19,632 (13,552, 27,113)	30,624 (23,481, 40,539)	26,123 (20,027, 35,460)	15,601 (11,612, 22,031)	0.0 (0.0, 0.0)	0.0 (0.0, 0.0)	4.9 (3.2, 8.0)	15.5 (10.4, 21.2)	17.0 (11.7, 23.5)	19.2 (14.7, 25.4)	17.2 (13.2, 23.4)	14.6 (10.9, 20.6)
Oesophagus cancer	0 (0, 0)	0 (0, 0)	1,038 (613, 1,861)	10,006 (7,123, 14,295)	27,320 (20,048, 39,020)	57,160 (44,606, 76,691)	72,457 (53,375, 100,479)	67,726 (46,376, 101,913)	0.0 (0.0, 0.0)	0.0 (0.0, 0.0)	2.2 (1.3, 3.9)	8.0 (5.7, 11.5)	8.4 (6.2, 12.0)	9.6 (7.5, 12.9)	8.9 (6.5, 12.3)	7.3 (5.0, 11.1)
Colon and rectum cancers	0 (0, 0)	0 (0, 0)	2,906 (1,339, 5,507)	23,153 (13,732, 35,689)	47,011 (28,788, 72,050)	87,895 (54,012, 132,698)	97,895 (56,043, 152,874)	122,015 (60,330, 201,223)	0.0 (0.0, 0.0)	0.0 (0.0, 0.0)	1.5 (0.7, 2.8)	4.7 (2.8, 7.3)	5.0 (3.1, 7.7)	5.6 (3.4, 8.5)	5.0 (2.9, 7.9)	4.2 (2.1, 7.0)
Liver cancer	0 (0, 0)	0 (0, 0)	8,400 (4,145, 15,088)	35,568 (18,871, 60,199)	88,716 (47,059, 149,224)	145,921 (76,800, 244,008)	167,083 (85,615, 282,505)	190,783 (92,551, 326,379)	0.0 (0.0, 0.0)	0.0 (0.0, 0.0)	6.1 (3.0, 10.9)	10.6 (5.6, 17.9)	10.1 (5.4, 17.0)	10.4 (5.5, 17.4)	10.6 (5.4, 18.0)	10.9 (5.3, 18.6)
Breast cancer	0 (0, 0)	0 (0, 0)	11,498 (8,888, 18,178)	169,356 (131,667, 226,473)	316,251 (253,056, 406,930)	390,233 (317,504, 494,932)	257,990 (211,916, 325,914)	179,449 (147,623, 226,094)	0.0 (0.0, 0.0)	0.0 (0.0, 0.0)	2.4 (1.9, 3.8)	7.1 (5.5, 9.5)	7.4 (5.9, 9.6)	8.2 (6.6, 10.3)	7.4 (6.1, 9.4)	6.7 (5.5, 8.4)
Larynx cancer	0 (0, 0)	0 (0, 0)	198 (96, 390)	1,386 (855, 2,107)	3,302 (2,185, 4,956)	8,280 (5,751, 12,098)	9,219 (6,247, 13,939)	6,341 (3,835, 10,216)	0.0 (0.0, 0.0)	0.0 (0.0, 0.0)	3.0 (1.4, 5.8)	9.6 (5.9, 14.6)	10.2 (6.8, 15.3)	11.3 (7.9, 16.6)	10.6 (7.2, 16.1)	8.6 (5.2, 13.8)
Diabetes mellitus	0 (0, 0)	-4,518 (-6,554, 1,422)	-31,800 (-44,201, -15,464)	-53,636 (-73,474, -25,853)	-127,679 (-175,256, -63,303)	-238,273 (-332,502, -117,832)	-317,175 (-443,835, -146,480)	-353,935 (-504,730, -166,953)	0.0 (0.0, 0.0)	-3.2 (-4.6, 1.0)	-7.7 (-10.7, -3.7)	-8.2 (-11.3, -4.0)	-8.0 (-11.0, -4.0)	-6.8 (-9.5, -3.4)	-5.8 (-8.1, -2.7)	-4.9 (-7.1, -2.3)
Alcohol use disorders	0 (0, 0)	15,517 (15,517, 15,517)	72,082 (72,082, 72,082)	161,981 (161,981, 161,981)	202,109 (202,109, 202,109)	222,743 (222,743, 222,743)	136,277 (136,277, 136,277)	70,148 (70,148, 70,148)	100.0 (100.0, 100.0)	100.0 (100.0, 100.0)	100.0 (100.0, 100.0)	100.0 (100.0, 100.0)	100.0 (100.0, 100.0)	100.0 (100.0, 100.0)	100.0 (100.0, 100.0)	100.0 (100.0, 100.0)

Cause of disease or injury	Alcohol-attributable YLL								Population Attributable Fraction							
	0 to 14	15 to 19	20 to 29	30 to 39	40 to 49	50 to 59	60 to 69	≥70	0 to 14	15 to 19	20 to 29	30 to 39	40 to 49	50 to 59	60 to 69	≥70
Epilepsy	0 (0, 0)	13,564 (10,037, 26,230)	53,064 (38,945, 75,940)	36,301 (27,654, 52,334)	23,984 (18,299, 33,406)	16,806 (12,637, 23,335)	10,622 (8,016, 14,943)	10,647 (8,187, 14,920)	0.0 (0.0, 0.0)	4.3 (3.2, 8.2)	9.3 (6.8, 13.4)	9.5 (7.3, 13.7)	9.9 (7.6, 13.9)	8.3 (6.2, 11.5)	6.7 (5.0, 9.4)	5.8 (4.5, 8.1)
Cardiovascular diseases	0 (0, 0)	27,300 (17,089, 58,652)	121,117 (51,097, 251,237)	277,069 (142,172, 515,726)	582,055 (299,759, 1,068,450)	985,884 (512,975, 1,817,671)	1,334,779 (596,187, 2,595,653)	2,884,235 (1,083,374, 5,274,465)	0.0 (0.0, 0.0)	2.2 (1.4, 4.7)	3.4 (1.4, 7.1)	4.4 (2.3, 8.2)	4.8 (2.5, 8.7)	4.3 (2.3, 8.0)	3.4 (1.5, 6.6)	3.6 (1.4, 6.6)
Hypertensive heart disease	0 (0, 0)	993 (467, 2,796)	6,721 (3,065, 12,894)	14,114 (6,738, 28,879)	29,718 (14,159, 62,395)	47,327 (22,806, 104,401)	57,882 (27,153, 139,787)	102,275 (47,653, 248,083)	0.0 (0.0, 0.0)	1.9 (0.9, 5.4)	4.5 (2.1, 8.7)	4.5 (2.1, 9.2)	4.5 (2.1, 9.4)	3.5 (1.7, 7.7)	2.7 (1.3, 6.4)	2.2 (1.0, 5.4)
Ischaemic heart disease	0 (0, 0)	6,027 (-673, 19,087)	26,869 (-36,692, 121,105)	67,719 (-56,112, 246,142)	136,486 (-111,984, 493,986)	345,793 (-110,114, 952,016)	705,871 (-89,078, 1,583,663)	2,250,207 (324,206, 3,913,283)	0.0 (0.0, 0.0)	1.9 (-0.2, 6.1)	2.3 (-3.1, 10.2)	2.7 (-2.2, 9.8)	2.5 (-2.1, 9.1)	3.2 (-1.0, 8.8)	3.7 (-0.5, 8.3)	5.4 (0.8, 9.5)
Ischaemic stroke	0 (0, 0)	-355 (-692, 10,535)	-2,350 (-4,754, 37,094)	-6,711 (-10,638, 27,498)	-28,514 (-44,481, 10,541)	-83,093 (-133,873, 3,886)	-193,786 (-357,553, 119,309)	-374,800 (-908,325, 608,166)	0.0 (0.0, 0.0)	-0.5 (-1.0, 15.0)	-1.3 (-2.6, 20.3)	-2.3 (-3.7, 9.5)	-3.9 (-6.1, 1.5)	-4.4 (-7.1, 0.2)	-3.2 (-5.9, 2.0)	-2.5 (-6.1, 4.1)
Haemorrhagic stroke	0 (0, 0)	19,936 (11,170, 37,260)	83,471 (48,443, 140,783)	179,873 (107,445, 301,407)	421,209 (249,979, 700,498)	643,470 (387,295, 1,071,963)	741,635 (440,390, 1,248,304)	873,892 (536,922, 1,470,015)	0.0 (0.0, 0.0)	6.6 (3.7, 12.4)	11.2 (6.5, 18.8)	11.3 (6.7, 18.9)	11.9 (7.1, 19.8)	10.2 (6.2, 17.0)	8.7 (5.2, 14.6)	7.7 (4.7, 13.0)
Cardiomyopathy, myocarditis, endocarditis	0 (0, 0)	698 (697, 763)	6,406 (6,403, 6,536)	22,074 (22,070, 22,186)	23,156 (23,153, 23,250)	32,387 (32,384, 32,471)	23,177 (23,174, 23,270)	32,661 (32,654, 32,802)	0.0 (0.0, 0.0)	0.5 (0.5, 0.6)	1.7 (1.7, 1.7)	4.9 (4.9, 4.9)	4.9 (4.9, 4.9)	5.9 (5.9, 5.9)	3.8 (3.8, 3.8)	2.8 (2.8, 2.8)
Digestive diseases	0 (0, 0)	138,717 (106,014, 180,452)	589,155 (479,834, 696,214)	769,389 (663,387, 881,615)	1,021,481 (891,927, 1,159,702)	1,286,535 (1,115,969, 1,483,950)	1,199,730 (1,018,451, 1,425,129)	806,377 (671,581, 1,013,490)	0.0 (0.0, 0.0)	12.9 (9.9, 16.8)	24.2 (19.7, 28.7)	28.7 (24.7, 32.9)	31.2 (27.3, 35.5)	27.8 (24.1, 32.0)	21.8 (18.5, 25.9)	11.5 (9.6, 14.4)
Cirrhosis of the liver	0 (0, 0)	136,720 (104,038, 177,924)	576,412 (469,316, 672,731)	747,867 (641,877, 849,941)	996,962 (865,663, 1,122,797)	1,258,845 (1,092,133, 1,445,173)	1,174,918 (995,632, 1,390,024)	780,190 (643,852, 979,473)	0.0 (0.0, 0.0)	32.1 (24.5, 41.8)	48.4 (39.4, 56.5)	51.1 (43.9, 58.1)	52.9 (45.9, 59.5)	48.6 (42.1, 55.7)	43.2 (36.6, 51.2)	34.6 (28.6, 43.5)
Pancreatitis	0 (0, 0)	1,997 (447, 5,760)	12,742 (3,184, 33,995)	21,522 (9,454, 48,096)	24,518 (7,703, 61,525)	27,690 (10,540, 66,283)	24,812 (9,970, 57,330)	26,187 (9,129, 60,413)	0.0 (0.0, 0.0)	6.4 (1.4, 18.6)	11.9 (3.0, 31.7)	14.2 (6.2, 31.7)	13.0 (4.1, 32.6)	12.0 (4.5, 28.6)	9.8 (4.0, 22.8)	8.5 (2.9, 19.5)
**Injuries**	1,183,488 (868,328, 1,944,651)	631,707 (500,245, 917,457)	1,741,687 (1,310,891, 2,413,762)	1,272,514 (1,010,716, 1,723,550)	1,030,665 (817,598, 1,391,671)	746,850 (621,288, 1,006,065)	542,473 (453,160, 750,356)	414,773 (340,266, 610,033)	5.1 (3.7, 8.3)	9.2 (7.3, 13.4)	13.4 (10.1, 18.5)	14.8 (11.7, 20.0)	16.2 (12.9, 21.9)	13.5 (11.3, 18.2)	10.6 (8.8, 14.6)	7.0 (5.7, 10.2)
Unintentional injuries	1,183,488 (868,328, 1,944,651)	474,577 (391,324, 687,743)	1,153,300 (946,103, 1,585,512)	854,860 (710,955, 1,164,704)	743,891 (615,273, 1,016,224)	567,817 (482,375, 783,100)	439,556 (368,829, 619,105)	360,141 (289,021, 543,285)	5.8 (4.2, 9.5)	13.1 (10.8, 19.0)	17.5 (14.4, 24.1)	17.7 (14.7, 24.0)	18.7 (15.4, 25.5)	15.0 (12.7, 20.7)	11.2 (9.4, 15.8)	7.0 (5.6, 10.6)
Road injury	1,183,488 (868,328, 1,944,651)	346,597 (272,058, 512,959)	754,239 (594,055, 1,072,680)	528,713 (425,306, 750,096)	475,500 (380,658, 683,122)	357,325 (283,989, 525,478)	263,087 (208,856, 392,539)	158,992 (127,514, 240,601)	23.4 (17.1, 38.4)	19.6 (15.4, 29.0)	23.5 (18.5, 33.4)	23.2 (18.6, 32.9)	23.4 (18.7, 33.6)	20.6 (16.4, 30.3)	19.6 (15.6, 29.3)	17.6 (14.1, 26.7)
Poisonings	0 (0, 0)	10,256 (5,242, 19,919)	41,073 (21,674, 68,064)	29,241 (16,437, 47,315)	27,026 (14,706, 44,067)	15,531 (8,749, 25,348)	11,438 (6,485, 19,228)	5,229 (3,408, 8,896)	0.0 (0.0, 0.0)	7.9 (4.1, 15.4)	13.1 (6.9, 21.8)	14.3 (8.1, 23.2)	13.3 (7.2, 21.7)	9.8 (5.5, 15.9)	6.8 (3.9, 11.5)	4.7 (3.1, 8.0)

Cause of disease or injury	Alcohol-attributable YLL								Population Attributable Fraction							
	0 to 14	15 to 19	20 to 29	30 to 39	40 to 49	50 to 59	60 to 69	≥70	0 to 14	15 to 19	20 to 29	30 to 39	40 to 49	50 to 59	60 to 69	≥70
Falls	0 (0, 0)	15,109 (8,082, 27,319)	52,799 (29,614, 84,418)	48,428 (28,810, 75,937)	50,907 (29,392, 80,844)	52,804 (30,636, 86,509)	61,127 (35,917, 105,602)	102,916 (63,386, 183,967)	0.0 (0.0, 0.0)	7.5 (4.0, 13.6)	13.1 (7.3, 20.9)	14.2 (8.4, 22.3)	14.6 (8.4, 23.1)	9.1 (5.3, 14.9)	5.6 (3.3, 9.6)	4.2 (2.6, 7.4)
Fire, heat and hot substances	0 (0, 0)	22,587 (10,511, 41,377)	79,039 (41,547, 132,350)	57,657 (33,709, 93,301)	32,482 (20,548, 50,999)	23,138 (16,287, 35,291)	15,999 (11,260, 24,933)	13,275 (10,106, 20,983)	0.0 (0.0, 0.0)	5.3 (2.5, 9.7)	9.8 (5.1, 16.4)	10.1 (5.9, 16.3)	11.3 (7.2, 17.8)	9.6 (6.7, 14.6)	7.5 (5.3, 11.6)	5.8 (4.4, 9.2)
Drowning	0 (0, 0)	32,164 (16,766, 58,477)	67,936 (37,532, 110,234)	51,206 (30,817, 79,674)	40,190 (23,451, 63,316)	26,420 (15,405, 42,599)	20,186 (11,445, 34,227)	14,831 (8,806, 26,614)	0.0 (0.0, 0.0)	7.8 (4.1, 14.1)	13.3 (7.4, 21.6)	14.4 (8.7, 22.4)	15.4 (9.0, 24.3)	10.9 (6.3, 17.6)	8.2 (4.7, 13.9)	5.5 (3.3, 9.8)
Exposure to mechanical forces	0 (0, 0)	9,022 (4,683, 16,712)	28,659 (15,568, 46,532)	26,895 (15,256, 43,088)	23,806 (13,289, 38,820)	13,614 (8,082, 22,036)	8,387 (4,858, 14,312)	4,438 (2,883, 8,000)	0.0 (0.0, 0.0)	7.0 (3.6, 12.9)	12.4 (6.7, 20.1)	13.6 (7.7, 21.8)	14.4 (8.0, 23.5)	10.4 (6.2, 16.9)	7.3 (4.2, 12.4)	4.7 (3.0, 8.4)
Other unintentional injuries	0 (0, 0)	38,843 (20,261, 72,340)	129,554 (72,261, 212,022)	112,721 (70,540, 176,563)	93,981 (60,566, 144,308)	78,984 (54,942, 115,863)	59,332 (40,583, 90,745)	60,461 (38,053, 99,723)	0.0 (0.0, 0.0)	7.0 (3.7, 13.1)	11.9 (6.6, 19.5)	12.7 (8.0, 19.9)	13.8 (8.9, 21.2)	11.4 (7.9, 16.7)	8.1 (5.5, 12.4)	5.8 (3.7, 9.6)
Intentional injuries	0 (0, 0)	157,130 (52,020, 316,338)	588,387 (207,083, 1,080,532)	417,654 (193,885, 709,200)	286,774 (128,108, 491,637)	179,033 (91,487, 297,941)	102,917 (54,178, 173,560)	54,633 (34,579, 89,713)	0.0 (0.0, 0.0)	4.9 (1.6, 9.8)	9.1 (3.2, 16.7)	11.1 (5.2, 18.9)	12.1 (5.4, 20.7)	10.3 (5.3, 17.2)	8.5 (4.4, 14.3)	6.5 (4.1, 10.7)
Self-harm	0 (0, 0)	110,847 (34,287, 218,293)	420,031 (141,931, 777,653)	285,531 (121,547, 492,726)	211,669 (85,344, 372,222)	138,694 (63,176, 239,972)	83,316 (39,659, 145,439)	45,294 (26,974, 77,293)	0.0 (0.0, 0.0)	5.5 (1.7, 10.8)	9.9 (3.3, 18.3)	12.3 (5.3, 21.3)	13.4 (5.4, 23.5)	10.7 (4.9, 18.5)	8.5 (4.0, 14.8)	6.4 (3.8, 10.9)
Interpersonal violence	0 (0, 0)	46,282 (14,629, 96,612)	168,356 (63,705, 306,494)	132,123 (70,356, 217,787)	75,105 (42,618, 119,676)	40,339 (28,508, 57,140)	19,601 (14,579, 27,254)	9,339 (7,610, 12,585)	0.0 (0.0, 0.0)	6.8 (2.2, 14.2)	11.4 (4.3, 20.7)	13.3 (7.1, 22.0)	13.8 (7.8, 22.0)	12.9 (9.2, 18.3)	11.1 (8.2, 15.4)	8.4 (6.9, 11.3)

Table A8. Global alcohol-attributable years of life lost (YLL) by age and cause in 2016 and 95% uncertainty intervals

Cause of disease or injury	Alcohol-attributable YLL								Population Attributable Fraction							
	0 to 14	15 to 19	20 to 29	30 to 39	40 to 49	50 to 59	60 to 69	≥70	0 to 14	15 to 19	20 to 29	30 to 39	40 to 49	50 to 59	60 to 69	≥70
**All Causes**	2,903,607 (2,126,090, 4,781,929)	3,557,228 (2,921,096, 5,021,533)	16,622,155 (13,520,172, 20,776,480)	18,117,906 (15,568,094, 21,616,419)	18,678,618 (16,274,110, 21,617,607)	19,048,367 (16,798,778, 21,877,731)	15,269,771 (13,207,843, 18,124,388)	12,397,840 (9,637,778, 16,434,235)	0.5 (0.4, 0.8)	6.9 (5.7, 9.7)	13.0 (10.6, 16.2)	13.3 (11.5, 15.9)	12.1 (10.5, 14.0)	9.3 (8.2, 10.7)	6.0 (5.2, 7.1)	3.4 (2.6, 4.5)
**Communicable, maternal, perinatal and nutritional conditions**	0 (0, 0)	229,045 (126,216, 586,882)	2,026,921 (976,096, 3,345,673)	2,874,660 (1,559,998, 4,489,738)	2,779,941 (1,425,886, 4,357,399)	2,269,199 (1,076,707, 3,760,614)	1,581,271 (784,545, 2,693,443)	1,430,200 (784,206, 2,443,201)	0.0 (0.0, 0.0)	1.5 (0.8, 3.9)	5.4 (2.6, 8.9)	6.8 (3.7, 10.6)	8.5 (4.3, 13.3)	9.1 (4.3, 15.1)	6.7 (3.3, 11.4)	4.1 (2.3, 7.0)
Tuberculosis	0 (0, 0)	171,379 (65,059, 426,199)	1,580,195 (539,665, 2,834,856)	1,994,188 (679,124, 3,489,016)	1,994,102 (652,083, 3,511,864)	1,724,802 (549,418, 3,159,153)	1,137,797 (356,739, 2,206,577)	733,353 (224,301, 1,511,186)	0.0 (0.0, 0.0)	8.5 (3.2, 21.0)	22.0 (7.5, 39.4)	24.6 (8.4, 43.1)	25.4 (8.3, 44.8)	22.0 (7.0, 40.3)	17.8 (5.6, 34.5)	12.8 (3.9, 26.4)
HIV AIDS	0 (0, 0)	20,034 (14,071, 106,461)	227,259 (157,146, 369,884)	597,910 (405,936, 971,513)	486,407 (332,014, 772,589)	165,711 (112,231, 268,604)	37,236 (24,655, 62,798)	5,519 (3,468, 11,994)	0.0 (0.0, 0.0)	1.0 (0.7, 5.1)	3.0 (2.1, 4.9)	3.5 (2.4, 5.6)	3.8 (2.6, 6.0)	3.5 (2.3, 5.6)	3.1 (2.1, 5.2)	2.3 (1.4, 4.9)
Lower respiratory infections	0 (0, 0)	37,632 (19,203, 115,550)	219,467 (111,208, 374,840)	282,562 (171,614, 436,769)	299,432 (174,099, 473,040)	378,686 (215,315, 602,226)	406,237 (201,153, 695,703)	691,328 (314,934, 1,235,003)	0.0 (0.0, 0.0)	2.2 (1.1, 6.6)	6.2 (3.1, 10.6)	8.4 (5.1, 13.0)	8.5 (4.9, 13.4)	7.4 (4.2, 11.7)	5.3 (2.6, 9.0)	4.0 (1.8, 7.1)
**Noncommunicable diseases**	0 (0, 0)	447,067 (386,273, 588,850)	2,807,807 (2,449,808, 3,167,945)	6,308,017 (5,588,148, 7,006,311)	9,546,024 (8,446,862, 10,597,044)	12,321,013 (10,903,848, 13,865,601)	11,019,644 (9,284,352, 13,167,234)	9,368,370 (6,626,540, 12,845,629)	0.0 (0.0, 0.0)	3.2 (2.8, 4.2)	7.7 (6.7, 8.7)	11.2 (9.9, 12.4)	9.9 (8.7, 11.0)	7.6 (6.8, 8.6)	5.1 (4.3, 6.1)	2.9 (2.1, 4.0)
Malignant neoplasms	0 (0, 0)	0 (0, 0)	83,062 (67,807, 115,516)	698,352 (577,752, 848,605)	1,809,074 (1,515,174, 2,165,230)	3,308,220 (2,830,321, 3,853,511)	3,309,138 (2,853,398, 3,832,616)	2,558,347 (2,200,190, 3,036,353)	0.0 (0.0, 0.0)	0.0 (0.0, 0.0)	1.1 (0.9, 1.5)	4.6 (3.8, 5.6)	5.7 (4.8, 6.8)	6.2 (5.3, 7.3)	5.5 (4.8, 6.4)	4.2 (3.6, 5.0)
Lip and oral cavity cancer	0 (0, 0)	0 (0, 0)	14,439 (9,397, 21,723)	142,729 (101,813, 174,531)	312,464 (241,935, 368,296)	509,480 (418,235, 582,998)	418,343 (349,563, 481,396)	216,760 (184,111, 251,937)	0.0 (0.0, 0.0)	0.0 (0.0, 0.0)	7.7 (5.0, 11.6)	30.1 (21.5, 36.8)	32.2 (24.9, 37.9)	35.4 (29.0, 40.5)	33.7 (28.2, 38.8)	28.7 (24.4, 33.4)
Other pharynx cancers	0 (0, 0)	0 (0, 0)	4,063 (2,740, 6,006)	54,435 (39,835, 66,299)	197,452 (154,319, 231,644)	409,137 (340,625, 464,058)	346,343 (290,020, 394,429)	162,496 (135,323, 189,340)	0.0 (0.0, 0.0)	0.0 (0.0, 0.0)	7.1 (4.8, 10.5)	28.4 (20.8, 34.6)	34.5 (27.0, 40.5)	39.8 (33.2, 45.2)	38.1 (31.9, 43.4)	30.8 (25.7, 35.9)
Oesophagus cancer	0 (0, 0)	0 (0, 0)	3,421 (2,516, 4,920)	40,030 (32,960, 48,003)	208,307 (176,700, 241,742)	610,624 (512,132, 701,615)	753,210 (616,738, 881,993)	531,746 (425,077, 645,176)	0.0 (0.0, 0.0)	0.0 (0.0, 0.0)	3.4 (2.5, 4.8)	12.7 (10.5, 15.3)	18.5 (15.7, 21.5)	22.3 (18.7, 25.7)	21.7 (17.7, 25.4)	17.8 (14.2, 21.6)
Colon and rectum cancers	0 (0, 0)	0 (0, 0)	16,001 (13,421, 20,345)	99,211 (80,954, 122,407)	224,803 (183,475, 274,366)	465,534 (382,371, 560,220)	617,142 (504,902, 739,291)	725,858 (596,080, 882,266)	0.0 (0.0, 0.0)	0.0 (0.0, 0.0)	3.8 (3.2, 4.8)	9.6 (7.8, 11.8)	10.8 (8.8, 13.1)	12.4 (10.1, 14.9)	12.8 (10.5, 15.3)	11.7 (9.6, 14.2)
Liver cancer	0 (0, 0)	0 (0, 0)	32,771 (19,900, 56,124)	180,796 (102,737, 269,422)	484,232 (269,833, 734,875)	747,549 (425,779, 1,118,601)	725,321 (431,001, 1,079,400)	626,272 (377,708, 934,453)	0.0 (0.0, 0.0)	0.0 (0.0, 0.0)	5.5 (3.3, 9.4)	11.5 (6.5, 17.1)	11.8 (6.6, 17.9)	12.4 (7.1, 18.6)	12.6 (7.5, 18.7)	12.4 (7.5, 18.5)
Breast cancer	0 (0, 0)	0 (0, 0)	11,498 (8,890, 18,192)	169,356 (131,667, 226,473)	316,251 (253,056, 406,930)	390,233 (317,504, 494,934)	257,990 (211,916, 325,914)	179,449 (147,624, 226,094)	0.0 (0.0, 0.0)	0.0 (0.0, 0.0)	2.4 (1.9, 3.8)	7.1 (5.5, 9.4)	7.4 (5.9, 9.5)	8.1 (6.6, 10.3)	7.4 (6.1, 9.3)	6.6 (5.4, 8.3)
Larynx cancer	0 (0, 0)	0 (0, 0)	870 (615, 1,321)	11,795 (8,772, 15,078)	65,565 (49,414, 82,500)	175,661 (136,098, 216,283)	190,788 (146,989, 236,531)	115,767 (86,932, 146,102)	0.0 (0.0, 0.0)	0.0 (0.0, 0.0)	4.1 (2.9, 6.2)	17.9 (13.3, 22.9)	21.7 (16.3, 27.3)	24.5 (19.0, 30.2)	24.0 (18.5, 29.8)	20.7 (15.6, 26.2)
Diabetes mellitus	0 (0, 0)	-3,079 (-5,498, 7,340)	-24,091 (-37,562, -3,950)	-38,606 (-61,225, -868)	-91,036 (-146,471, -4,280)	-165,149 (-271,793, -5,576)	-223,058 (-370,515, -1,602)	-248,580 (-429,541, -77)	0.0 (0.0, 0.0)	-1.1 (-2.0, 2.7)	-2.8 (-4.4, -0.5)	-2.7 (-4.3, -0.1)	-2.6 (-4.2, -0.1)	-2.3 (-3.7, -0.1)	-2.1 (-3.5, 0.0)	-1.9 (-3.3, 0.0)

Cause of disease or injury	Alcohol-attributable YLL								Population Attributable Fraction							
	0 to 14	15 to 19	20 to 29	30 to 39	40 to 49	50 to 59	60 to 69	≥70	0 to 14	15 to 19	20 to 29	30 to 39	40 to 49	50 to 59	60 to 69	≥70
Alcohol use disorders	0 (0, 0)	72,113 (72,113, 72,113)	512,186 (512,186, 512,186)	1,218,131 (1,218,131, 1,218,131)	1,470,520 (1,470,520, 1,470,520)	1,436,186 (1,436,186, 1,436,186)	795,591 (795,591, 795,591)	285,125 (285,125, 285,125)	100.0 (100.0, 100.0)	100.0 (100.0, 100.0)	100.0 (100.0, 100.0)	100.0 (100.0, 100.0)	100.0 (100.0, 100.0)	100.0 (100.0, 100.0)	100.0 (100.0, 100.0)	100.0 (100.0, 100.0)
Epilepsy	0 (0, 0)	51,753 (40,124, 84,371)	219,537 (171,484, 272,463)	172,320 (135,680, 212,601)	125,592 (99,332, 152,684)	81,811 (64,797, 99,017)	52,216 (41,065, 64,334)	38,439 (30,725, 47,247)	0.0 (0.0, 0.0)	5.9 (4.6, 9.6)	15.1 (11.8, 18.8)	17.2 (13.6, 21.3)	18.7 (14.8, 22.7)	16.9 (13.4, 20.4)	14.6 (11.5, 18.0)	11.5 (9.2, 14.1)
Cardiovascular diseases	0 (0, 0)	50,678 (38,503, 99,699)	287,583 (166,256, 469,018)	740,512 (468,064, 1,056,745)	1,409,874 (861,514, 1,947,918)	2,438,758 (1,518,783, 3,400,884)	3,013,206 (1,581,077, 4,691,115)	4,556,477 (1,948,432, 7,730,132)	0.0 (0.0, 0.0)	1.8 (1.4, 3.6)	3.2 (1.8, 5.2)	4.2 (2.6, 5.9)	4.1 (2.5, 5.6)	3.8 (2.4, 5.4)	3.2 (1.7, 4.9)	3.0 (1.3, 5.1)
Hypertensive heart disease	0 (0, 0)	2,988 (2,162, 5,577)	30,343 (23,202, 39,193)	71,251 (55,896, 91,050)	149,630 (117,852, 191,491)	261,913 (206,837, 337,320)	358,744 (277,944, 468,706)	542,570 (413,826, 731,059)	0.0 (0.0, 0.0)	3.1 (2.2, 5.8)	9.1 (7.0, 11.8)	10.2 (8.0, 13.0)	10.4 (8.2, 13.3)	9.4 (7.4, 12.1)	8.3 (6.4, 10.9)	6.8 (5.2, 9.2)
Ischaemic heart disease	0 (0, 0)	6,366 (-2,963, 24,242)	9,333 (-96,963, 145,018)	67,633 (-180,042, 302,321)	142,275 (-359,875, 507,033)	461,257 (-413,152, 1,150,690)	890,989 (-472,460, 2,138,165)	2,454,760 (-178,794, 4,963,202)	0.0 (0.0, 0.0)	0.8 (-0.4, 3.2)	0.3 (-2.7, 4.1)	0.8 (-2.0, 3.4)	0.8 (-1.9, 2.7)	1.3 (-1.2, 3.3)	1.8 (-0.9, 4.3)	3.1 (-0.2, 6.2)
Ischaemic stroke	0 (0, 0)	-805 (-1,205, 10,267)	-4,919 (-8,265, 37,882)	-9,854 (-16,761, 30,517)	-38,691 (-61,378, 20,746)	-104,552 (-182,651, 39,577)	-284,311 (-525,596, 165,780)	-561,601 (-1,195,383, 638,186)	0.0 (0.0, 0.0)	-0.7 (-1.0, 8.6)	-1.3 (-2.1, 9.7)	-1.5 (-2.5, 4.6)	-2.2 (-3.5, 1.2)	-2.1 (-3.7, 0.8)	-2.0 (-3.7, 1.2)	-2.0 (-4.2, 2.3)
Haemorrhagic stroke	0 (0, 0)	35,510 (25,178, 59,631)	202,698 (149,351, 275,230)	439,402 (330,514, 592,677)	979,655 (728,223, 1,321,066)	1,603,361 (1,194,799, 2,146,768)	1,917,646 (1,389,758, 2,661,428)	2,032,781 (1,485,840, 2,851,536)	0.0 (0.0, 0.0)	5.7 (4.1, 9.6)	10.4 (7.7, 14.1)	11.3 (8.5, 15.2)	11.8 (8.8, 16.0)	10.8 (8.0, 14.4)	9.8 (7.1, 13.6)	9.0 (6.5, 12.6)
Cardiomyopathy, myocarditis, endocarditis	0 (0, 0)	6,618 (5,398, 26,533)	50,128 (48,172, 79,271)	172,080 (170,761, 188,872)	177,005 (175,771, 190,107)	216,778 (215,261, 235,111)	130,138 (128,368, 149,969)	87,968 (86,549, 101,310)	0.0 (0.0, 0.0)	1.9 (1.5, 7.4)	4.9 (4.7, 7.8)	12.3 (12.2, 13.5)	11.5 (11.5, 12.4)	12.3 (12.2, 13.3)	7.7 (7.6, 8.9)	3.9 (3.8, 4.4)
Digestive diseases	0 (0, 0)	275,603 (222,219, 357,921)	1,729,530 (1,427,130, 1,980,284)	3,517,309 (2,956,160, 3,949,174)	4,821,999 (4,137,281, 5,384,981)	5,221,188 (4,565,681, 5,804,043)	4,072,551 (3,542,963, 4,604,696)	2,178,563 (1,875,194, 2,555,774)	0.0 (0.0, 0.0)	12.8 (10.3, 16.6)	28.3 (23.3, 32.4)	37.7 (31.7, 42.3)	39.9 (34.2, 44.5)	35.7 (31.2, 39.7)	28.3 (24.6, 32.0)	15.4 (13.2, 18.0)
Cirrhosis of the liver	0 (0, 0)	267,824 (214,014, 350,598)	1,619,949 (1,326,458, 1,856,937)	3,299,755 (2,741,416, 3,715,851)	4,585,744 (3,913,385, 5,126,868)	5,029,517 (4,381,001, 5,606,650)	3,931,714 (3,409,846, 4,451,590)	2,068,797 (1,765,570, 2,435,157)	0.0 (0.0, 0.0)	28.8 (23.0, 37.7)	49.5 (40.6, 56.8)	53.9 (44.8, 60.7)	55.3 (47.2, 61.9)	52.5 (45.7, 58.5)	48.2 (41.8, 54.6)	39.2 (33.5, 46.2)
Pancreatitis	0 (0, 0)	7,778 (5,179, 13,015)	109,580 (75,111, 152,667)	217,554 (159,556, 285,018)	236,255 (170,419, 313,154)	191,672 (142,516, 254,689)	140,837 (103,038, 193,168)	109,765 (77,015, 158,511)	0.0 (0.0, 0.0)	11.0 (7.3, 18.5)	27.2 (18.6, 37.9)	32.2 (23.6, 42.2)	31.6 (22.8, 41.9)	28.3 (21.0, 37.6)	23.5 (17.2, 32.3)	18.7 (13.1, 27.1)
**Injuries**	2,903,607 (2,126,090, 4,781,929)	2,881,116 (2,239,423, 4,122,814)	11,787,427 (8,885,571, 15,381,522)	8,935,228 (6,944,490, 11,447,470)	6,352,654 (5,011,585, 8,112,934)	4,458,155 (3,570,242, 5,650,068)	2,668,856 (2,155,182, 3,440,043)	1,599,270 (1,277,245, 2,148,025)	5.2 (3.8, 8.6)	12.8 (9.9, 18.3)	21.8 (16.4, 28.5)	24.0 (18.7, 30.8)	25.2 (19.9, 32.1)	23.2 (18.5, 29.4)	18.7 (15.1, 24.1)	12.3 (9.8, 16.5)
Unintentional injuries	2,903,607 (2,126,090, 4,781,929)	2,109,853 (1,640,007, 3,106,486)	7,789,015 (5,989,625, 10,513,141)	5,812,585 (4,582,070, 7,674,748)	4,463,606 (3,512,833, 5,892,377)	3,261,178 (2,579,654, 4,296,736)	2,084,744 (1,647,487, 2,778,363)	1,331,850 (1,028,870, 1,848,431)	5.9 (4.3, 9.6)	16.0 (12.5, 23.6)	26.1 (20.1, 35.2)	27.3 (21.5, 36.1)	27.6 (21.7, 36.4)	24.6 (19.5, 32.4)	19.4 (15.4, 25.9)	12.3 (9.5, 17.1)

Cause of disease or injury	Alcohol-attributable YLL								Population Attributable Fraction							
	0 to 14	15 to 19	20 to 29	30 to 39	40 to 49	50 to 59	60 to 69	≥70	0 to 14	15 to 19	20 to 29	30 to 39	40 to 49	50 to 59	60 to 69	≥70
Road injury	2,903,607 (2,126,090, 4,781,929)	1,530,612 (1,076,326, 2,404,466)	5,277,563 (3,749,479, 7,674,680)	3,584,816 (2,562,597, 5,167,116)	2,653,415 (1,889,190, 3,833,112)	1,805,434 (1,286,646, 2,653,859)	1,073,349 (768,312, 1,606,141)	523,043 (376,581, 812,471)	23.0 (16.9, 37.9)	20.2 (14.2, 31.8)	29.0 (20.6, 42.2)	30.0 (21.4, 43.2)	30.0 (21.3, 43.3)	27.5 (19.6, 40.4)	25.0 (17.9, 37.4)	20.7 (14.9, 32.2)
Poisonings	0 (0, 0)	30,512 (19,360, 50,271)	170,746 (108,517, 239,245)	130,496 (86,563, 179,098)	111,053 (73,450, 152,734)	73,858 (49,974, 100,165)	49,357 (33,030, 68,762)	22,667 (14,598, 33,542)	0.0 (0.0, 0.0)	10.0 (6.3, 16.4)	20.6 (13.1, 28.8)	22.9 (15.2, 31.5)	21.7 (14.4, 29.9)	19.3 (13.1, 26.2)	14.3 (9.5, 19.9)	9.4 (6.1, 14.0)
Falls	0 (0, 0)	60,278 (37,213, 96,315)	360,025 (220,100, 505,871)	419,439 (264,477, 581,509)	440,080 (273,312, 612,128)	428,722 (267,782, 604,599)	364,879 (230,276, 527,596)	410,414 (260,984, 617,132)	0.0 (0.0, 0.0)	10.3 (6.4, 16.5)	22.4 (13.7, 31.4)	24.7 (15.5, 34.2)	25.3 (15.7, 35.2)	20.9 (13.0, 29.4)	13.9 (8.8, 20.2)	8.8 (5.6, 13.2)
Fire, heat and hot substances	0 (0, 0)	39,617 (26,229, 63,513)	188,603 (126,804, 265,920)	177,606 (127,590, 238,115)	123,304 (91,858, 159,147)	103,708 (78,967, 132,547)	67,665 (50,199, 89,033)	42,505 (30,672, 59,514)	0.0 (0.0, 0.0)	6.7 (4.4, 10.8)	14.6 (9.8, 20.6)	17.4 (12.5, 23.3)	20.9 (15.5, 26.9)	20.1 (15.3, 25.7)	15.9 (11.8, 20.9)	10.4 (7.5, 14.6)
Drowning	0 (0, 0)	201,900 (115,676, 330,269)	574,176 (343,867, 812,121)	413,479 (272,429, 562,014)	283,148 (187,367, 383,772)	191,586 (126,983, 259,909)	120,968 (78,821, 169,061)	64,848 (41,310, 95,917)	0.0 (0.0, 0.0)	11.6 (6.6, 19.0)	23.4 (14.0, 33.0)	26.0 (17.1, 35.3)	26.1 (17.3, 35.4)	22.5 (14.9, 30.6)	17.7 (11.5, 24.8)	11.4 (7.3, 16.9)
Exposure to mechanical forces	0 (0, 0)	58,616 (33,526, 97,412)	310,835 (178,549, 446,493)	262,118 (161,505, 365,460)	194,047 (121,427, 267,887)	129,333 (79,853, 182,216)	66,515 (40,813, 95,405)	25,056 (15,837, 37,474)	0.0 (0.0, 0.0)	10.8 (6.2, 18.0)	22.9 (13.2, 32.9)	25.0 (15.4, 34.8)	25.8 (16.2, 35.6)	23.1 (14.3, 32.6)	18.1 (11.1, 26.0)	10.8 (6.8, 16.1)
Other unintentional injuries	0 (0, 0)	188,319 (111,178, 304,702)	907,066 (546,159, 1,288,946)	824,632 (529,346, 1,131,203)	658,558 (435,398, 892,232)	528,536 (371,067, 704,505)	342,012 (234,788, 468,455)	243,317 (158,329, 354,587)	0.0 (0.0, 0.0)	10.4 (6.1, 16.8)	21.9 (13.2, 31.2)	24.4 (15.6, 33.4)	25.1 (16.6, 34.0)	22.9 (16.1, 30.5)	17.2 (11.8, 23.6)	11.3 (7.4, 16.5)
Intentional injuries	0 (0, 0)	771,262 (302,537, 1,404,501)	3,998,412 (1,607,492, 6,283,985)	3,122,643 (1,456,441, 4,685,718)	1,889,048 (903,803, 2,807,991)	1,196,977 (619,726, 1,764,148)	584,112 (306,760, 880,916)	267,420 (143,371, 419,099)	0.0 (0.0, 0.0)	8.2 (3.2, 14.9)	16.5 (6.6, 26.0)	19.7 (9.2, 29.5)	20.8 (10.0, 30.9)	20.0 (10.3, 29.4)	16.6 (8.7, 25.0)	12.2 (6.5, 19.1)
Self-harm	0 (0, 0)	334,809 (164,126, 575,225)	1,969,066 (940,996, 2,980,023)	1,783,334 (943,154, 2,598,840)	1,239,782 (635,295, 1,811,676)	891,654 (473,546, 1,303,207)	473,010 (244,275, 715,974)	236,506 (123,318, 372,829)	0.0 (0.0, 0.0)	8.5 (4.2, 14.6)	18.4 (8.8, 27.8)	23.0 (12.2, 33.6)	23.8 (12.2, 34.7)	21.9 (11.6, 32.1)	17.5 (9.1, 26.5)	12.8 (6.7, 20.1)
Interpersonal violence	0 (0, 0)	436,453 (131,095, 838,454)	2,029,346 (632,678, 3,340,295)	1,339,309 (520,016, 2,103,665)	649,266 (269,241, 1,006,277)	305,324 (152,456, 465,596)	111,102 (61,525, 165,611)	30,914 (19,615, 46,105)	0.0 (0.0, 0.0)	11.1 (3.3, 21.2)	20.7 (6.5, 34.1)	22.4 (8.7, 35.2)	22.0 (9.1, 34.1)	20.1 (10.0, 30.6)	16.9 (9.4, 25.3)	10.8 (6.9, 16.2)

Table A9. Global alcohol-attributable years of life lost (YLL) by cause and sex in 2016 and 95% uncertainty intervals

Cause of disease or injury	Men		Women		Total	
	Alcohol-attributable burden	PAF (%)	Alcohol-attributable burden	PAF (%)	Alcohol-attributable burden	PAF (%)
**All Causes**	73,818,321 (60,643,464, 89,463,387)	8.5 (7.0, 10.3)	20,379,330 (17,373,605, 28,076,315)	3.2 (2.7, 4.4)	94,197,651 (80,416,185, 113,816,088)	6.3 (5.4, 7.6)
**Communicable, maternal, perinatal and nutritional conditions**	9,389,778 (3,749,688, 15,634,056)	3.0 (1.2, 4.9)	2,371,258 (1,055,781, 5,759,481)	0.8 (0.4, 2.0)	11,761,036 (5,949,448, 19,233,749)	2.0 (1.0, 3.2)
Tuberculosis	7,013,327 (1,467,619, 12,878,351)	25.3 (5.3, 46.4)	1,589,136 (261,948, 4,308,993)	10.4 (1.7, 28.1)	8,602,463 (2,842,089, 15,627,665)	20.0 (6.6, 36.3)
HIV AIDS	1,134,133 (667,780, 1,759,506)	3.6 (2.1, 5.5)	400,425 (236,282, 1,198,714)	1.7 (1.0, 5.0)	1,534,557 (1,046,054, 2,551,848)	2.8 (1.9, 4.6)
Lower respiratory infections	1,242,319 (494,961, 2,150,623)	2.1 (0.8, 3.7)	381,698 (183,468, 820,651)	0.7 (0.3, 1.5)	1,624,016 (892,594, 2,698,128)	1.5 (0.8, 2.4)
**Noncommunicable diseases**	31,590,884 (26,783,698, 35,494,654)	8.1 (6.9, 9.1)	10,858,688 (8,944,946, 14,487,210)	3.9 (3.2, 5.2)	42,449,572 (37,059,290, 48,392,985)	6.3 (5.5, 7.2)
Malignant neoplasms	6,874,334 (5,643,844, 8,150,971)	7.0 (5.7, 8.3)	2,333,512 (1,983,318, 2,937,502)	2.9 (2.5, 3.7)	9,207,846 (7,844,452, 10,815,476)	5.2 (4.4, 6.1)
Lip and oral cavity cancer	1,190,268 (944,913, 1,380,773)	38.0 (30.2, 44.1)	207,187 (150,142, 279,436)	16.3 (11.8, 22.0)	1,397,455 (1,120,943, 1,628,945)	31.7 (25.5, 37.0)
Other pharynx cancers	925,347 (751,793, 1,061,704)	40.7 (33.1, 46.7)	86,083 (63,559, 116,669)	16.5 (12.2, 22.4)	1,011,430 (827,539, 1,162,436)	36.2 (29.6, 41.6)
Oesophagus cancer	1,447,611 (1,190,786, 1,676,140)	24.7 (20.3, 28.6)	167,982 (125,765, 232,346)	8.7 (6.5, 12.1)	1,615,593 (1,341,046, 1,878,273)	20.8 (17.2, 24.1)
Colon and rectum cancers	1,163,832 (923,975, 1,407,646)	16.4 (13.0, 19.8)	258,859 (153,913, 398,818)	5.0 (3.0, 7.7)	1,422,692 (1,165,123, 1,716,629)	11.6 (9.5, 13.9)
Liver cancer	1,724,981 (830,500, 2,709,565)	12.4 (5.9, 19.4)	445,688 (232,490, 751,024)	10.1 (5.2, 16.9)	2,170,669 (1,249,250, 3,258,422)	11.8 (6.8, 17.7)
Breast cancer	0 (0, 0)	0.0 (0.0, 0.0)	1,145,328 (923,032, 1,472,427)	7.4 (6.0, 9.5)	1,145,328 (923,034, 1,472,444)	7.4 (6.0, 9.5)
Larynx cancer	422,295 (320,200, 526,828)	24.8 (18.8, 31.0)	22,384 (15,134, 33,490)	10.1 (6.9, 15.2)	444,679 (341,888, 551,713)	23.1 (17.8, 28.7)
Diabetes mellitus	228,064 (20,331, 535,338)	1.9 (0.2, 4.3)	-773,082 (-1,075,821, -367,510)	-6.5 (-9.0, -3.1)	-545,018 (-893,063, -8,936)	-2.2 (-3.7, 0.0)
Alcohol use disorders	4,694,018 (4,694,018, 4,694,018)	100.0 (100.0, 100.0)	810,708 (810,708, 810,708)	100.0 (100.0, 100.0)	5,504,726 (5,504,726, 5,504,726)	100.0 (100.0, 100.0)
Epilepsy	548,887 (404,804, 697,075)	13.8 (10.2, 17.6)	154,341 (115,587, 226,187)	5.4 (4.1, 7.9)	703,228 (552,482, 885,471)	10.3 (8.1, 13.0)
Cardiovascular diseases	4,612,408 (1,687,369, 6,904,193)	3.3 (1.2, 4.9)	3,328,202 (1,619,279, 6,307,389)	3.8 (1.8, 7.2)	7,940,610 (4,634,197, 11,665,378)	3.5 (2.0, 5.1)
Hypertensive heart disease	718,114 (541,599, 877,727)	14.3 (10.8, 17.5)	156,755 (74,386, 351,153)	3.3 (1.6, 7.4)	874,870 (683,893, 1,133,337)	9.0 (7.0, 11.6)
Ischaemic heart disease	289,088 (-2,446,249, 2,046,723)	0.4 (-3.1, 2.6)	1,288,766 (-404,654, 3,415,997)	3.3 (-1.0, 8.7)	1,577,854 (-1,525,455, 4,267,470)	1.3 (-1.3, 3.6)
Ischaemic stroke	-128,322 (-404,246, 264,497)	-1.0 (-3.2, 2.1)	-314,810 (-551,990, 208,863)	-3.4 (-6.0, 2.3)	-443,132 (-795,857, 304,770)	-2.0 (-3.6, 1.4)
Haemorrhagic stroke	3,088,678 (2,046,966, 4,224,725)	10.6 (7.1, 14.6)	2,089,594 (1,244,722, 3,500,215)	9.6 (5.7, 16.2)	5,178,272 (3,817,823, 7,056,800)	10.2 (7.5, 13.9)
Cardiomyopathy, myocarditis, endocarditis	644,849 (635,765, 761,822)	11.2 (11.0, 13.2)	107,898 (107,880, 108,476)	3.4 (3.4, 3.4)	752,747 (743,732, 869,864)	8.4 (8.3, 9.7)
Digestive diseases	14,633,173 (12,012,027, 16,740,132)	34.2 (28.1, 39.1)	5,005,007 (4,275,582, 5,827,061)	21.8 (18.6, 25.3)	19,638,180 (16,851,434, 22,081,098)	29.8 (25.6, 33.6)
Cirrhosis of the liver	13,842,779 (11,292,536, 15,915,486)	51.1 (41.6, 58.7)	4,891,724 (4,168,660, 5,658,590)	43.2 (36.8, 50.0)	18,734,504 (15,986,121, 21,108,494)	48.7 (41.6, 54.9)
Pancreatitis	790,394 (537,775, 1,035,622)	35.3 (24.0, 46.3)	113,282 (41,298, 272,989)	11.5 (4.2, 27.6)	903,676 (655,819, 1,211,712)	28.0 (20.3, 37.5)
**Injuries**	32,837,659 (24,335,738, 44,047,114)	20.6 (15.3, 27.6)	7,149,383 (5,582,225, 10,147,512)	10.4 (8.1, 14.7)	39,987,042 (30,932,584, 52,936,780)	17.5 (13.5, 23.2)
Unintentional injuries	23,007,100 (17,209,296, 31,696,187)	21.5 (16.1, 29.7)	5,417,488 (4,383,188, 7,801,039)	11.5 (9.3, 16.5)	28,424,588 (22,077,766, 39,043,781)	18.4 (14.3, 25.3)
Road injury	14,919,846 (10,213,552, 22,374,457)	28.4 (19.4, 42.5)	3,908,949 (3,033,250, 5,881,523)	22.4 (17.4, 33.7)	18,828,795 (13,458,639, 28,121,302)	26.9 (19.2, 40.2)
Poisonings	431,458 (243,746, 633,577)	13.0 (7.4, 19.1)	134,564 (73,292, 223,941)	5.6 (3.0, 9.3)	566,022 (370,893, 790,275)	9.9 (6.5, 13.8)
Falls	1,792,248 (1,015,923, 2,620,333)	18.0 (10.2, 26.3)	281,175 (162,451, 460,630)	6.1 (3.5, 10.0)	2,073,423 (1,293,159, 2,928,017)	14.3 (8.9, 20.1)
Fire, heat and hot substances	469,602 (297,191, 655,671)	13.5 (8.5, 18.8)	230,902 (133,861, 378,251)	5.4 (3.1, 8.9)	700,503 (501,646, 948,275)	9.0 (6.5, 12.2)
Drowning	1,547,156 (896,394, 2,257,586)	11.8 (6.8, 17.2)	238,101 (135,417, 388,527)	3.8 (2.2, 6.2)	1,785,257 (1,125,142, 2,517,146)	9.2 (5.8, 13.0)
Exposure to mechanical forces	911,081 (507,870, 1,335,647)	15.1 (8.4, 22.1)	110,383 (61,736, 181,500)	4.4 (2.4, 7.2)	1,021,464 (615,673, 1,454,873)	11.9 (7.2, 16.9)
Other unintentional injuries	2,935,709 (1,726,598, 4,233,731)	16.1 (9.5, 23.2)	513,415 (319,154, 811,841)	5.3 (3.3, 8.3)	3,449,124 (2,227,936, 4,790,044)	12.3 (8.0, 17.1)
Intentional injuries	9,830,560 (3,381,114, 15,874,413)	18.6 (6.4, 30.1)	1,731,895 (726,761, 3,069,208)	8.0 (3.4, 14.2)	11,562,454 (5,196,760, 17,827,258)	15.6 (7.0, 24.0)
Self-harm	5,441,565 (2,168,297, 8,477,121)	24.3 (9.7, 37.9)	1,250,089 (485,943, 2,246,305)	9.8 (3.8, 17.5)	6,691,655 (3,401,392, 9,984,946)	19.0 (9.7, 28.4)
Interpersonal violence	4,388,994 (1,193,184, 7,402,746)	19.8 (5.4, 33.3)	481,806 (234,397, 824,963)	8.9 (4.3, 15.3)	4,870,800 (1,767,011, 7,919,898)	17.6 (6.4, 28.7)

PAF: Population Attributable Fraction

Table A10. Global alcohol-attributable years lived with disability (YLD) by age and cause in 2016 among men and 95% uncertainty intervals

Cause of disease or injury	Alcohol-attributable YLD								Population attributable fraction (%)							
	0 to 14	15 to 19	20 to 29	30 to 39	40 to 49	50 to 59	60 to 69	≥70	0 to 14	15 to 19	20 to 29	30 to 39	40 to 49	50 to 59	60 to 69	≥70
**All Causes**	74,601 (66,068, 95,045)	628,921 (566,958, 728,942)	3,676,383 (3,345,223, 4,035,805)	3,909,925 (3,500,832, 4,359,902)	3,677,449 (3,198,670, 4,222,265)	3,068,411 (2,616,972, 3,603,288)	2,025,151 (1,688,637, 2,446,456)	1,291,746 (1,034,230, 1,641,578)	0.2 (0.1, 0.2)	3.4 (3.1, 4.0)	8.1 (7.4, 8.9)	8.2 (7.3, 9.1)	7.4 (6.4, 8.4)	6.1 (5.2, 7.1)	4.5 (3.7, 5.4)	2.8 (2.3, 3.6)
**Communicable, maternal, perinatal and nutritional conditions**	0 (0, 0)	9,147 (2,547, 22,955)	91,203 (29,404, 160,683)	117,107 (47,514, 192,711)	121,083 (45,505, 202,473)	101,711 (30,722, 181,228)	78,161 (19,688, 149,105)	48,401 (12,445, 97,073)	0.0 (0.0, 0.0)	0.3 (0.1, 0.7)	1.4 (0.5, 2.5)	2.0 (0.8, 3.3)	2.6 (1.0, 4.4)	3.2 (1.0, 5.7)	3.9 (1.0, 7.5)	3.6 (0.9, 7.2)
Tuberculosis	0 (0, 0)	8,489 (1,879, 21,906)	77,247 (15,282, 147,416)	87,417 (17,375, 161,573)	94,129 (18,303, 174,147)	87,552 (16,975, 166,305)	71,844 (13,487, 142,621)	44,458 (8,103, 93,028)	0.0 (0.0, 0.0)	10.3 (2.3, 26.5)	27.2 (5.4, 51.9)	30.0 (6.0, 55.5)	31.0 (6.0, 57.3)	28.3 (5.5, 53.7)	25.8 (4.8, 51.3)	21.8 (4.0, 45.5)
HIV AIDS	0 (0, 0)	392 (247, 2,148)	12,758 (7,740, 19,610)	28,352 (17,240, 42,608)	25,227 (15,424, 37,229)	11,926 (7,261, 17,292)	4,051 (2,462, 6,088)	1,242 (765, 1,936)	0.0 (0.0, 0.0)	0.9 (0.6, 5.1)	3.8 (2.3, 5.8)	4.5 (2.7, 6.7)	4.9 (3.0, 7.2)	4.7 (2.8, 6.8)	4.0 (2.4, 6.0)	2.8 (1.7, 4.4)
Lower respiratory infections	0 (0, 0)	266 (107, 586)	1,198 (421, 2,142)	1,337 (519, 2,313)	1,727 (695, 2,969)	2,234 (1,040, 3,609)	2,266 (1,000, 3,791)	2,701 (1,031, 4,797)	0.0 (0.0, 0.0)	2.6 (1.0, 5.7)	7.1 (2.5, 12.7)	8.4 (3.3, 14.6)	9.3 (3.7, 15.9)	9.5 (4.4, 15.4)	8.2 (3.6, 13.6)	6.9 (2.6, 12.2)
**Noncommunicable diseases**	40,056 (40,056, 40,056)	436,726 (429,875, 452,996)	2,586,138 (2,551,541, 2,625,033)	2,475,335 (2,436,052, 2,523,857)	1,972,885 (1,911,867, 2,053,151)	1,441,713 (1,355,374, 1,549,310)	832,168 (736,122, 957,198)	448,097 (331,349, 592,126)	0.2 (0.2, 0.2)	3.1 (3.1, 3.2)	7.4 (7.3, 7.5)	6.7 (6.6, 6.8)	5.0 (4.8, 5.2)	3.5 (3.3, 3.7)	2.1 (1.9, 2.5)	1.1 (0.8, 1.5)
Malignant neoplasms	0 (0, 0)	0 (0, 0)	594 (481, 779)	5,070 (4,159, 5,981)	16,958 (14,138, 19,837)	41,973 (36,050, 47,736)	54,324 (47,220, 62,055)	55,276 (48,234, 63,258)	0.0 (0.0, 0.0)	0.0 (0.0, 0.0)	1.1 (0.9, 1.5)	5.6 (4.6, 6.6)	8.1 (6.7, 9.5)	8.8 (7.6, 10.0)	7.0 (6.1, 8.0)	5.4 (4.7, 6.2)
Lip and oral cavity cancer	0 (0, 0)	0 (0, 0)	187 (130, 269)	1,957 (1,482, 2,335)	4,521 (3,675, 5,181)	9,879 (8,273, 11,137)	10,234 (8,625, 11,555)	8,316 (6,937, 9,542)	100.0 (100.0, 100.0)	0.0 (0.0, 0.0)	9.4 (6.5, 13.5)	32.6 (24.7, 38.9)	39.1 (31.8, 44.8)	44.2 (37.0, 49.9)	43.4 (36.6, 49.0)	38.6 (32.2, 44.3)
Other pharynx cancers	0 (0, 0)	0 (0, 0)	32 (21, 48)	516 (385, 617)	2,405 (1,973, 2,732)	6,540 (5,558, 7,300)	6,418 (5,445, 7,251)	3,414 (2,826, 3,942)	100.0 (100.0, 100.0)	0.0 (0.0, 0.0)	8.9 (5.7, 13.1)	37.8 (28.1, 45.1)	44.4 (36.4, 50.4)	47.7 (40.5, 53.2)	45.5 (38.6, 51.4)	39.1 (32.4, 45.2)
Oesophagus cancer	0 (0, 0)	0 (0, 0)	17 (12, 24)	246 (205, 285)	1,544 (1,274, 1,779)	4,698 (3,888, 5,362)	7,185 (5,776, 8,413)	6,493 (5,204, 7,759)	100.0 (100.0, 100.0)	0.0 (0.0, 0.0)	5.1 (3.7, 7.2)	19.3 (16.1, 22.4)	25.4 (21.0, 29.2)	27.5 (22.7, 31.4)	26.4 (21.2, 30.9)	23.6 (18.9, 28.2)
Colon and rectum cancers	0 (0, 0)	0 (0, 0)	199 (164, 246)	1,229 (962, 1,506)	4,048 (3,094, 4,981)	10,501 (8,082, 12,811)	18,310 (14,335, 22,207)	26,855 (21,761, 32,228)	100.0 (100.0, 100.0)	0.0 (0.0, 0.0)	6.2 (5.1, 7.7)	14.0 (11.0, 17.2)	16.3 (12.4, 20.0)	18.1 (13.9, 22.1)	18.5 (14.5, 22.5)	19.1 (15.5, 23.0)
Liver cancer	0 (0, 0)	0 (0, 0)	141 (68, 261)	899 (417, 1,434)	3,095 (1,422, 4,975)	5,606 (2,618, 8,778)	6,202 (3,090, 9,714)	5,887 (2,966, 9,458)	0.0 (0.0, 0.0)	0.0 (0.0, 0.0)	5.5 (2.6, 10.1)	11.4 (5.3, 18.2)	12.3 (5.6, 19.7)	13.2 (6.2, 20.7)	13.3 (6.6, 20.9)	14.0 (7.1, 22.6)
Breast cancer	0 (0, 0)	0 (0, 0)	0 (0, 0)	0 (0, 0)	0 (0, 0)	0 (0, 0)	0 (0, 0)	0 (0, 0)	0.0 (0.0, 0.0)	0.0 (0.0, 0.0)	0.0 (0.0, 1.4)	0.0 (0.0, 0.0)	0.0 (0.0, 0.0)	0.0 (0.0, 0.0)	0.0 (0.0, 0.0)	0.0 (0.0, 0.0)
Larynx cancer	0 (0, 0)	0 (0, 0)	18 (12, 27)	224 (168, 285)	1,347 (1,023, 1,682)	4,749 (3,655, 5,849)	5,976 (4,571, 7,418)	4,311 (3,223, 5,442)	100.0 (100.0, 100.0)	0.0 (0.0, 0.0)	6.1 (4.1, 9.3)	20.5 (15.4, 26.1)	24.7 (18.7, 30.8)	27.4 (21.1, 33.8)	26.7 (20.4, 33.1)	23.9 (17.9, 30.2)
Diabetes mellitus	0 (0, 0)	1,561 (-153, 4,838)	12,727 (2,925, 27,246)	28,018 (5,753, 61,920)	53,865 (10,927, 120,574)	70,549 (11,276, 157,783)	66,249 (6,092, 151,712)	55,592 (937, 129,685)	0.0 (0.0, 0.0)	1.1 (-0.1, 3.4)	1.5 (0.3, 3.2)	1.7 (0.3, 3.7)	1.8 (0.4, 4.1)	2.0 (0.3, 4.4)	2.1 (0.2, 4.9)	2.3 (0.0, 5.4)
Alcohol use disorders	40,056 (40,056, 40,056)	397,655 (397,655, 397,655)	2,411,766 (2,411,766, 2,411,766)	2,286,974 (2,286,974, 2,286,974)	1,710,325 (1,710,325, 1,710,325)	1,118,279 (1,118,279, 1,118,279)	537,944 (537,944, 537,944)	201,726 (201,726, 201,726)	100.0 (100.0, 100.0)	100.0 (100.0, 100.0)	100.0 (100.0, 100.0)	100.0 (100.0, 100.0)	100.0 (100.0, 100.0)	100.0 (100.0, 100.0)	100.0 (100.0, 100.0)	100.0 (100.0, 100.0)

Cause of disease or injury	Alcohol-attributable YLD								Population attributable fraction (%)							
	0 to 14	15 to 19	20 to 29	30 to 39	40 to 49	50 to 59	60 to 69	≥70	0 to 14	15 to 19	20 to 29	30 to 39	40 to 49	50 to 59	60 to 69	≥70
Epilepsy	0 (0, 0)	25,832 (19,415, 37,972)	118,468 (88,399, 149,262)	95,758 (71,812, 119,485)	84,480 (63,676, 104,570)	69,557 (52,463, 87,129)	50,779 (38,276, 63,955)	40,474 (30,426, 51,336)	0.0 (0.0, 0.0)	6.9 (5.2, 10.1)	17.4 (13.0, 21.9)	19.8 (14.8, 24.7)	21.5 (16.2, 26.6)	21.0 (15.9, 26.4)	19.7 (14.9, 24.8)	18.1 (13.6, 23.0)
Cardiovascular diseases	0 (0, 0)	433 (-64, 1,413)	2,234 (-2,365, 8,280)	6,386 (-3,143, 15,831)	15,606 (-4,181, 33,217)	29,389 (-7,700, 67,106)	35,827 (-26,937, 103,989)	41,756 (-55,932, 154,157)	0.0 (0.0, 0.0)	0.2 (0.0, 0.6)	0.3 (-0.4, 1.3)	0.8 (-0.4, 2.0)	1.0 (-0.3, 2.2)	1.1 (-0.3, 2.5)	0.9 (-0.7, 2.6)	0.7 (-1.0, 2.7)
Hypertensive heart disease	0 (0, 0)	0 (0, 0)	7 (5, 9)	2,667 (2,030, 3,228)	8,276 (6,321, 9,970)	14,287 (10,872, 17,358)	19,875 (14,873, 24,351)	30,529 (22,534, 37,587)	0.0 (0.0, 0.0)	5.8 (3.9, 13.8)	15.6 (11.5, 19.0)	14.6 (11.1, 17.7)	16.2 (12.4, 19.5)	16.2 (12.3, 19.6)	15.4 (11.5, 18.8)	14.5 (10.7, 17.8)
Ischaemic heart disease	0 (0, 0)	12 (-390, 549)	-547 (-4,640, 3,579)	-1,091 (-9,617, 4,928)	-2,616 (-18,561, 3,781)	-2,915 (-29,340, 9,619)	-2,242 (-44,286, 29,560)	-3,459 (-74,040, 64,069)	0.0 (0.0, 0.0)	0.1 (-1.9, 2.6)	-0.5 (-4.7, 3.6)	-0.5 (-4.2, 2.2)	-0.6 (-4.2, 0.9)	-0.4 (-4.1, 1.3)	-0.2 (-4.9, 3.3)	-0.3 (-6.1, 5.3)
Ischaemic stroke	0 (0, 0)	-333 (-656, 239)	-1,845 (-3,653, 2,871)	-2,991 (-6,483, 4,264)	-8,861 (-18,801, 7,785)	-16,844 (-40,062, 17,698)	-26,393 (-68,436, 31,259)	-38,484 (-107,924, 49,165)	0.0 (0.0, 0.0)	-1.5 (-3.0, 1.1)	-2.1 (-4.1, 3.3)	-1.9 (-4.1, 2.7)	-2.1 (-4.4, 1.8)	-1.8 (-4.2, 1.8)	-1.8 (-4.6, 2.1)	-1.8 (-5.0, 2.3)
Haemorrhagic stroke	0 (0, 0)	564 (350, 891)	3,819 (2,550, 5,200)	6,803 (4,600, 9,164)	17,149 (11,576, 23,259)	31,544 (21,203, 42,657)	41,545 (27,474, 57,057)	50,386 (33,050, 69,542)	0.0 (0.0, 0.0)	6.3 (3.9, 10.0)	11.5 (7.7, 15.6)	12.4 (8.4, 16.7)	13.4 (9.0, 18.2)	13.1 (8.8, 17.7)	12.5 (8.2, 17.1)	12.4 (8.1, 17.1)
Cardiomyopathy, myocarditis, endocarditis	0 (0, 0)	190 (173, 326)	801 (729, 1,237)	997 (951, 1,371)	1,658 (1,605, 2,233)	3,316 (3,241, 4,190)	3,043 (2,976, 3,888)	2,785 (2,740, 3,235)	0.0 (0.0, 0.0)	2.1 (1.9, 3.7)	2.4 (2.2, 3.7)	4.1 (3.9, 5.6)	5.6 (5.4, 7.6)	7.1 (6.9, 9.0)	5.9 (5.8, 7.6)	4.1 (4.1, 4.8)
Digestive diseases	0 (0, 0)	11,244 (8,098, 16,230)	40,348 (32,192, 47,767)	53,128 (43,366, 61,077)	91,651 (75,632, 103,963)	111,967 (93,096, 127,381)	87,044 (71,844, 100,124)	53,272 (43,652, 62,094)	0.0 (0.0, 0.0)	9.2 (6.6, 13.2)	12.6 (10.0, 14.9)	11.9 (9.7, 13.7)	14.8 (12.3, 16.8)	14.9 (12.4, 17.0)	11.9 (9.8, 13.7)	8.8 (7.2, 10.2)
Cirrhosis of the liver	0 (0, 0)	11,133 (7,997, 16,117)	39,429 (31,329, 46,821)	51,423 (41,844, 59,278)	89,207 (73,346, 101,446)	109,355 (90,614, 124,759)	84,722 (69,707, 97,885)	51,179 (41,785, 59,894)	0.0 (0.0, 0.0)	25.8 (18.5, 37.3)	47.0 (37.4, 55.9)	52.5 (42.7, 60.5)	56.6 (46.5, 64.3)	55.9 (46.3, 63.8)	53.6 (44.1, 62.0)	49.6 (40.5, 58.0)
Pancreatitis	0 (0, 0)	111 (74, 158)	920 (610, 1,243)	1,705 (1,136, 2,260)	2,444 (1,589, 3,259)	2,612 (1,742, 3,454)	2,322 (1,542, 3,107)	2,093 (1,386, 2,832)	0.0 (0.0, 0.0)	20.9 (14.0, 29.9)	35.1 (23.3, 47.4)	38.5 (25.6, 51.0)	40.5 (26.3, 54.0)	40.9 (27.3, 54.1)	39.1 (26.0, 52.4)	37.0 (24.5, 50.0)
**Injuries**	34,545 (26,012, 54,989)	183,049 (120,494, 273,864)	999,042 (683,661, 1,338,782)	1,317,484 (931,882, 1,737,147)	1,583,481 (1,138,632, 2,091,590)	1,524,987 (1,102,800, 2,013,425)	1,114,823 (810,715, 1,487,411)	795,249 (571,677, 1,100,065)	1.6 (1.2, 2.5)	14.1 (9.3, 21.0)	25.9 (17.7, 34.7)	26.8 (19.0, 35.3)	28.1 (20.2, 37.1)	26.7 (19.3, 35.2)	24.2 (17.6, 32.3)	19.7 (14.1, 27.2)
Unintentional injuries	34,545 (26,012, 54,989)	169,450 (106,190, 261,387)	915,171 (608,415, 1,253,952)	1,219,035 (837,969, 1,649,133)	1,478,725 (1,031,660, 1,986,615)	1,436,821 (1,021,692, 1,937,060)	1,061,421 (761,835, 1,436,466)	766,261 (542,327, 1,069,217)	1.7 (1.3, 2.7)	14.9 (9.3, 22.9)	27.4 (18.2, 37.5)	28.7 (19.7, 38.8)	30.0 (20.9, 40.3)	28.3 (20.1, 38.2)	25.2 (18.1, 34.1)	20.1 (14.2, 28.1)
Road injury	34,545 (26,012, 54,989)	37,174 (24,818, 57,491)	221,093 (153,959, 318,568)	331,226 (231,307, 475,083)	439,772 (306,017, 629,682)	429,728 (295,974, 618,440)	312,468 (216,792, 454,741)	210,536 (146,341, 313,002)	26.4 (19.9, 42.1)	22.8 (15.2, 35.3)	32.0 (22.3, 46.1)	32.5 (22.7, 46.7)	33.9 (23.6, 48.5)	32.7 (22.5, 47.0)	30.8 (21.4, 44.8)	27.7 (19.3, 41.2)
Poisonings	0 (0, 0)	1,849 (971, 2,948)	7,631 (4,230, 11,008)	7,587 (4,315, 10,806)	7,322 (4,171, 10,406)	5,778 (3,332, 8,300)	3,639 (2,101, 5,293)	2,147 (1,219, 3,215)	0.0 (0.0, 0.0)	15.5 (8.1, 24.7)	27.4 (15.2, 39.5)	28.5 (16.2, 40.7)	29.6 (16.9, 42.1)	27.6 (15.9, 39.7)	24.3 (14.0, 35.4)	19.1 (10.8, 28.6)
Falls	0 (0, 0)	56,986 (30,183, 91,251)	295,255 (168,924, 420,623)	395,487 (235,517, 554,170)	479,437 (285,220, 670,969)	505,966 (308,007, 710,937)	404,447 (247,276, 573,488)	336,652 (199,522, 495,854)	0.0 (0.0, 0.0)	15.8 (8.3, 25.2)	28.5 (16.3, 40.6)	29.8 (17.8, 41.8)	30.5 (18.2, 42.7)	28.5 (17.3, 40.0)	24.7 (15.1, 35.0)	19.1 (11.3, 28.2)
Fire, heat and hot substances	0 (0, 0)	9,503 (4,944, 15,703)	47,253 (25,954, 68,761)	61,166 (34,966, 87,185)	62,649 (36,377, 88,909)	53,565 (31,956, 76,050)	33,472 (20,225, 47,640)	19,683 (11,775, 28,846)	0.0 (0.0, 0.0)	13.6 (7.1, 22.5)	26.5 (14.5, 38.5)	28.0 (16.0, 39.9)	29.1 (16.9, 41.3)	27.6 (16.5, 39.2)	24.9 (15.0, 35.4)	19.7 (11.8, 28.9)

Cause of disease or injury	Alcohol-attributable YLD								Population attributable fraction (%)							
	0 to 14	15 to 19	20 to 29	30 to 39	40 to 49	50 to 59	60 to 69	≥70	0 to 14	15 to 19	20 to 29	30 to 39	40 to 49	50 to 59	60 to 69	≥70
Drowning	0 (0, 0)	768 (384, 1,296)	4,055 (2,140, 5,993)	4,827 (2,641, 7,052)	5,533 (3,090, 8,025)	4,902 (2,814, 7,155)	3,461 (2,007, 5,081)	4,630 (2,481, 7,053)	0.0 (0.0, 0.0)	12.8 (6.4, 21.7)	25.6 (13.5, 37.8)	26.6 (14.5, 38.8)	27.5 (15.4, 39.9)	25.2 (14.5, 36.8)	21.9 (12.7, 32.1)	20.2 (10.8, 30.8)
Exposure to mechanical forces	0 (0, 0)	16,393 (8,639, 26,431)	99,256 (55,636, 142,022)	136,388 (78,805, 192,687)	167,746 (97,098, 238,383)	157,811 (92,268, 225,280)	108,800 (63,908, 156,865)	64,793 (37,657, 96,443)	0.0 (0.0, 0.0)	15.7 (8.3, 25.4)	28.8 (16.2, 41.3)	29.8 (17.2, 42.1)	30.8 (17.8, 43.8)	28.8 (16.8, 41.1)	25.2 (14.8, 36.4)	19.5 (11.3, 29.0)
Other unintentional injuries	0 (0, 0)	46,776 (23,128, 79,851)	240,628 (125,731, 359,744)	282,353 (152,242, 416,772)	316,265 (174,522, 463,713)	279,071 (158,294, 411,716)	195,134 (112,633, 288,612)	127,821 (72,764, 195,164)	0.0 (0.0, 0.0)	11.3 (5.6, 19.3)	23.5 (12.3, 35.1)	24.6 (13.3, 36.3)	25.8 (14.2, 37.8)	23.7 (13.4, 35.0)	20.7 (12.0, 30.7)	15.7 (8.9, 24.0)
Intentional injuries	0 (0, 0)	13,599 (3,272, 26,365)	83,871 (22,558, 138,183)	98,448 (30,106, 158,636)	104,757 (32,225, 167,936)	88,166 (32,402, 139,873)	53,402 (21,480, 85,289)	28,988 (12,652, 48,117)	0.0 (0.0, 0.0)	8.4 (2.0, 16.3)	16.4 (4.4, 27.1)	14.8 (4.5, 23.8)	14.9 (4.6, 23.9)	13.7 (5.0, 21.7)	13.5 (5.4, 21.6)	12.5 (5.4, 20.7)
Self-harm	0 (0, 0)	1,077 (257, 2,032)	7,668 (2,254, 12,562)	10,780 (3,580, 17,281)	12,843 (4,275, 20,383)	12,552 (4,921, 19,657)	9,256 (3,740, 14,699)	7,419 (2,933, 12,325)	0.0 (0.0, 0.0)	11.7 (2.8, 22.1)	22.8 (6.7, 37.3)	23.9 (7.9, 38.3)	24.7 (8.2, 39.3)	23.7 (9.3, 37.1)	21.0 (8.5, 33.3)	17.1 (6.8, 28.5)
Interpersonal violence	0 (0, 0)	12,523 (3,016, 24,324)	76,203 (20,405, 125,643)	87,668 (26,771, 141,419)	91,914 (28,420, 147,349)	75,614 (27,544, 120,386)	44,146 (17,697, 70,697)	21,569 (9,686, 35,784)	0.0 (0.0, 0.0)	11.1 (2.7, 21.6)	23.0 (6.1, 37.8)	24.6 (7.5, 39.7)	25.6 (7.9, 41.0)	24.1 (8.8, 38.4)	21.2 (8.5, 33.9)	15.8 (7.1, 26.2)

Table A11. Global alcohol-attributable years lived with disability (YLD) by age and cause in 2016 among women and 95% uncertainty intervals

Cause of disease or injury	Alcohol-attributable YLD								Population Attributable Fraction							
	0 to 14	15 to 19	20 to 29	30 to 39	40 to 49	50 to 59	60 to 69	≥70	0 to 14	15 to 19	20 to 29	30 to 39	40 to 49	50 to 59	60 to 69	≥70
**All Causes**	47,256 (41,352, 61,150)	329,222 (303,386, 384,010)	1,599,821 (1,499,345, 1,773,692)	1,458,283 (1,354,308, 1,659,278)	1,140,142 (998,623, 1,402,860)	853,836 (718,605, 1,120,334)	537,471 (405,475, 797,887)	504,179 (325,160, 862,593)	0.1 (0.1, 0.1)	1.6 (1.5, 1.9)	3.2 (3.0, 3.5)	2.7 (2.5, 3.1)	2.0 (1.7, 2.4)	1.6 (1.3, 2.0)	1.1 (0.8, 1.6)	0.8 (0.5, 1.4)
**Communicable, maternal, perinatal and nutritional conditions**	0 (0, 0)	5,632 (1,611, 16,631)	37,999 (13,679, 83,573)	36,564 (18,534, 78,919)	30,021 (14,553, 66,239)	17,610 (6,790, 41,155)	12,471 (3,880, 31,017)	9,969 (3,293, 26,227)	0.0 (0.0, 0.0)	0.1 (0.0, 0.4)	0.4 (0.1, 0.9)	0.4 (0.2, 1.0)	0.5 (0.2, 1.0)	0.4 (0.2, 1.0)	0.5 (0.1, 1.2)	0.5 (0.2, 1.2)
Tuberculosis	0 (0, 0)	4,927 (762, 14,455)	27,938 (3,908, 68,342)	22,081 (3,615, 54,172)	19,768 (3,280, 48,871)	13,531 (2,336, 34,376)	10,520 (1,801, 28,204)	8,221 (1,441, 24,062)	0.0 (0.0, 0.0)	7.0 (1.1, 20.4)	14.6 (2.0, 35.8)	13.9 (2.3, 34.1)	13.4 (2.2, 33.1)	10.3 (1.8, 26.2)	8.4 (1.4, 22.5)	6.1 (1.1, 17.9)
HIV AIDS	0 (0, 0)	463 (295, 2,740)	9,339 (5,580, 19,386)	13,802 (8,335, 32,721)	9,464 (5,768, 23,326)	3,067 (1,974, 8,353)	915 (597, 3,303)	226 (148, 1,401)	0.0 (0.0, 0.0)	0.9 (0.6, 5.1)	2.3 (1.4, 4.8)	2.3 (1.4, 5.5)	2.3 (1.4, 5.6)	1.7 (1.1, 4.5)	1.3 (0.8, 4.7)	0.8 (0.5, 4.8)
Lower respiratory infections	0 (0, 0)	242 (142, 532)	721 (390, 1,252)	681 (390, 1,160)	789 (440, 1,333)	1,012 (653, 1,576)	1,035 (687, 1,601)	1,522 (991, 2,318)	0.0 (0.0, 0.0)	2.4 (1.4, 5.4)	4.7 (2.6, 8.2)	5.0 (2.9, 8.6)	5.1 (2.8, 8.5)	5.2 (3.3, 8.1)	4.4 (2.9, 6.7)	3.7 (2.4, 5.7)
**Noncommunicable diseases**	23,244 (23,244, 23,244)	231,105 (227,016, 244,192)	1,180,548 (1,154,587, 1,226,774)	971,752 (928,125, 1,040,142)	561,841 (477,992, 677,495)	289,910 (181,122, 440,681)	59,165 (-64,064, 244,867)	11,712 (-173,234, 307,366)	0.1 (0.1, 0.1)	1.5 (1.5, 1.6)	3.1 (3.0, 3.2)	2.3 (2.2, 2.4)	1.2 (1.0, 1.4)	0.6 (0.4, 0.9)	0.1 (-0.1, 0.6)	0.0 (-0.3, 0.6)
Malignant neoplasms	0 (0, 0)	0 (0, 0)	582 (486, 765)	5,710 (4,813, 6,963)	16,107 (13,575, 19,567)	24,995 (21,344, 30,118)	23,853 (20,662, 28,697)	25,840 (21,582, 32,231)	0.0 (0.0, 0.0)	0.0 (0.0, 0.0)	1.0 (0.8, 1.3)	3.6 (3.0, 4.4)	4.5 (3.8, 5.5)	4.8 (4.1, 5.8)	4.1 (3.6, 5.0)	3.3 (2.8, 4.2)
Lip and oral cavity cancer	0 (0, 0)	0 (0, 0)	106 (71, 157)	748 (552, 976)	1,231 (900, 1,627)	2,174 (1,644, 2,816)	2,157 (1,611, 2,874)	2,559 (1,861, 3,555)	100.0 (100.0, 100.0)	0.0 (0.0, 0.0)	5.9 (4.0, 8.8)	17.0 (12.5, 22.2)	17.4 (12.8, 23.1)	19.2 (14.5, 24.8)	16.9 (12.6, 22.5)	14.9 (10.9, 20.7)
Other pharynx cancers	0 (0, 0)	0 (0, 0)	23 (13, 36)	149 (97, 200)	378 (267, 503)	810 (603, 1,055)	741 (545, 989)	495 (354, 686)	100.0 (100.0, 100.0)	0.0 (0.0, 0.0)	6.4 (3.7, 10.1)	19.3 (12.5, 26.0)	20.4 (14.4, 27.1)	21.5 (16.0, 28.1)	18.4 (13.6, 24.6)	15.0 (10.7, 20.8)
Oesophagus cancer	0 (0, 0)	0 (0, 0)	8 (5, 13)	62 (45, 82)	239 (180, 314)	588 (449, 768)	892 (635, 1,225)	1,100 (748, 1,658)	100.0 (100.0, 100.0)	0.0 (0.0, 0.0)	3.2 (2.0, 5.2)	9.3 (6.8, 12.3)	10.3 (7.7, 13.5)	10.9 (8.3, 14.3)	9.6 (6.8, 13.2)	7.9 (5.4, 11.9)
Colon and rectum cancers	0 (0, 0)	0 (0, 0)	41 (22, 71)	361 (215, 538)	1,134 (687, 1,682)	2,618 (1,597, 3,879)	3,449 (1,956, 5,308)	5,688 (2,816, 9,374)	100.0 (100.0, 100.0)	0.0 (0.0, 0.0)	2.1 (1.1, 3.6)	5.3 (3.2, 7.9)	5.6 (3.4, 8.4)	6.0 (3.6, 8.8)	5.4 (3.0, 8.2)	4.5 (2.2, 7.4)
Liver cancer	0 (0, 0)	0 (0, 0)	45 (21, 79)	196 (103, 326)	555 (288, 930)	1,151 (597, 1,916)	1,721 (874, 2,908)	2,894 (1,420, 4,898)	0.0 (0.0, 0.0)	0.0 (0.0, 0.0)	5.5 (2.6, 9.7)	9.8 (5.2, 16.4)	9.8 (5.1, 16.4)	10.2 (5.3, 17.1)	10.6 (5.4, 17.8)	11.8 (5.8, 19.9)
Breast cancer	0 (0, 0)	0 (0, 0)	354 (284, 467)	4,158 (3,362, 5,215)	12,458 (10,155, 15,613)	17,357 (14,151, 21,676)	14,562 (12,005, 18,133)	12,808 (10,498, 16,119)	100.0 (100.0, 100.0)	0.0 (0.0, 0.0)	3.6 (2.9, 4.7)	8.3 (6.7, 10.4)	8.8 (7.2, 11.0)	9.5 (7.7, 11.8)	8.7 (7.1, 10.8)	7.3 (6.0, 9.2)
Larynx cancer	0 (0, 0)	0 (0, 0)	5 (3, 9)	36 (23, 53)	113 (75, 166)	298 (204, 430)	330 (218, 492)	295 (176, 478)	100.0 (100.0, 100.0)	0.0 (0.0, 0.0)	3.6 (2.0, 6.1)	9.7 (6.2, 14.3)	10.7 (7.1, 15.8)	12.4 (8.5, 17.9)	11.0 (7.2, 16.4)	8.7 (5.2, 14.2)
Diabetes mellitus	0 (0, 0)	-5,345 (-7,685, -2,234)	-62,527 (-86,451, -35,092)	-121,100 (-166,135, -68,466)	-234,337 (-322,699, -134,595)	-278,608 (-385,858, -160,477)	-250,042 (-349,780, -140,601)	-208,519 (-295,240, -113,944)	0.0 (0.0, 0.0)	-4.2 (-6.1, -1.8)	-9.2 (-12.8, -5.2)	-9.1 (-12.4, -5.1)	-9.9 (-13.6, -5.7)	-8.6 (-12.0, -5.0)	-8.1 (-11.3, -4.6)	-7.2 (-10.2, -3.9)
Alcohol use disorders	23,244 (23,244, 23,244)	209,827 (209,827, 209,827)	1,153,260 (1,153,260, 1,153,260)	1,012,534 (1,012,534, 1,012,534)	711,674 (711,674, 711,674)	490,995 (490,995, 490,995)	240,030 (240,030, 240,030)	118,739 (118,739, 118,739)	100.0 (100.0, 100.0)	100.0 (100.0, 100.0)	100.0 (100.0, 100.0)	100.0 (100.0, 100.0)	100.0 (100.0, 100.0)	100.0 (100.0, 100.0)	100.0 (100.0, 100.0)	100.0 (100.0, 100.0)

Cause of disease or injury	Alcohol-attributable YLD								Population Attributable Fraction							
	0 to 14	15 to 19	20 to 29	30 to 39	40 to 49	50 to 59	60 to 69	≥70	0 to 14	15 to 19	20 to 29	30 to 39	40 to 49	50 to 59	60 to 69	≥70
Epilepsy	0 (0, 0)	14,843 (11,700, 23,941)	54,591 (41,840, 74,693)	40,932 (31,657, 56,431)	35,316 (27,206, 48,235)	25,926 (20,065, 35,562)	18,051 (14,032, 25,160)	16,577 (12,965, 23,108)	0.0 (0.0, 0.0)	4.5 (3.5, 7.2)	9.1 (7.0, 12.4)	9.1 (7.0, 12.5)	9.5 (7.3, 12.9)	8.1 (6.2, 11.1)	7.0 (5.5, 9.8)	5.9 (4.7, 8.3)
Cardiovascular diseases	0 (0, 0)	574 (33, 3,831)	2,679 (-2,180, 25,335)	3,612 (-5,655, 30,452)	-5,967 (-31,745, 40,015)	-21,248 (-74,940, 57,064)	-15,496 (-99,818, 120,395)	21,302 (-135,512, 305,692)	0.0 (0.0, 0.0)	0.2 (0.0, 1.4)	0.4 (-0.4, 4.1)	0.4 (-0.7, 3.5)	-0.4 (-2.0, 2.5)	-0.8 (-2.8, 2.2)	-0.4 (-2.5, 3.1)	0.3 (-1.8, 4.1)
Hypertensive heart disease	0 (0, 0)	0 (0, 0)	3 (1, 6)	1,341 (598, 2,562)	3,781 (1,688, 7,286)	4,681 (2,085, 9,233)	5,670 (2,543, 11,127)	11,569 (5,230, 22,605)	0.0 (0.0, 0.0)	2.7 (1.3, 11.3)	6.2 (2.9, 14.0)	4.3 (1.9, 8.1)	4.3 (1.9, 8.3)	3.5 (1.6, 6.9)	3.0 (1.3, 5.8)	2.6 (1.2, 5.2)
Ischaemic heart disease	0 (0, 0)	381 (-99, 1,160)	2,184 (-2,237, 8,323)	4,459 (-4,283, 16,576)	6,966 (-9,684, 29,166)	16,618 (-9,618, 49,620)	29,439 (-8,317, 68,837)	68,724 (-12,568, 134,197)	0.0 (0.0, 0.0)	2.4 (-0.6, 7.4)	3.0 (-3.1, 11.4)	2.8 (-2.7, 10.4)	2.2 (-3.1, 9.4)	3.1 (-1.8, 9.3)	3.9 (-1.1, 9.2)	5.0 (-0.9, 9.7)
Ischaemic stroke	0 (0, 0)	-800 (-1,051, 1,964)	-5,174 (-7,731, 16,123)	-10,973 (-16,609, 10,712)	-35,500 (-55,494, -5,082)	-73,406 (-122,624, -9,859)	-88,912 (-169,921, 27,087)	-114,453 (-269,801, 134,515)	0.0 (0.0, 0.0)	-2.9 (-3.8, 7.1)	-4.8 (-7.1, 14.9)	-5.6 (-8.4, 5.4)	-7.1 (-11.1, -1.0)	-6.9 (-11.5, -0.9)	-5.3 (-10.2, 1.6)	-4.0 (-9.4, 4.7)
Haemorrhagic stroke	0 (0, 0)	926 (557, 1,527)	5,320 (3,098, 8,417)	8,322 (5,014, 13,074)	18,202 (10,772, 28,734)	29,769 (17,924, 47,231)	37,061 (22,561, 59,268)	53,451 (32,794, 85,379)	0.0 (0.0, 0.0)	8.3 (5.0, 13.7)	13.3 (7.7, 21.0)	13.1 (7.9, 20.5)	13.4 (7.9, 21.1)	11.9 (7.2, 18.9)	10.4 (6.4, 16.7)	9.8 (6.0, 15.6)
Cardiomyopathy, myocarditis, endocarditis	0 (0, 0)	67 (67, 68)	346 (346, 350)	463 (463, 467)	584 (584, 589)	1,090 (1,090, 1,097)	1,247 (1,246, 1,253)	2,012 (2,012, 2,018)	0.0 (0.0, 0.0)	0.6 (0.6, 0.6)	0.8 (0.8, 0.8)	1.6 (1.6, 1.6)	1.9 (1.9, 1.9)	2.7 (2.7, 2.7)	2.7 (2.7, 2.7)	1.9 (1.9, 1.9)
Digestive diseases	0 (0, 0)	11,206 (8,975, 14,170)	31,963 (27,249, 36,759)	30,064 (26,021, 34,295)	39,047 (34,165, 44,312)	47,850 (41,422, 54,755)	42,770 (36,741, 49,873)	37,773 (32,276, 45,049)	0.0 (0.0, 0.0)	8.6 (6.9, 10.9)	9.5 (8.1, 10.9)	5.1 (4.5, 5.9)	4.0 (3.5, 4.5)	5.7 (4.9, 6.5)	6.1 (5.2, 7.1)	5.3 (4.5, 6.3)
Cirrhosis of the liver	0 (0, 0)	11,153 (8,896, 14,027)	31,650 (26,934, 36,255)	29,623 (25,604, 33,549)	38,482 (33,579, 43,293)	47,025 (41,060, 53,519)	41,926 (36,098, 48,681)	36,873 (31,318, 43,848)	0.0 (0.0, 0.0)	33.5 (26.7, 42.1)	49.3 (41.9, 56.5)	51.1 (44.1, 57.8)	53.5 (46.6, 60.1)	50.1 (43.8, 57.0)	46.3 (39.8, 53.7)	42.2 (35.8, 50.2)
Pancreatitis	0 (0, 0)	52 (2, 156)	314 (45, 816)	441 (63, 1,154)	564 (-118, 1,878)	825 (106, 2,155)	844 (198, 2,014)	900 (276, 2,006)	0.0 (0.0, 0.0)	6.8 (0.2, 20.2)	13.2 (1.9, 34.4)	12.2 (1.7, 31.8)	8.6 (-1.8, 28.7)	10.2 (1.3, 26.7)	10.2 (2.4, 24.3)	9.9 (3.0, 22.0)
**Injuries**	24,012 (18,108, 37,906)	92,486 (65,357, 138,491)	381,274 (279,391, 531,908)	449,967 (341,184, 616,171)	548,280 (415,553, 755,262)	546,316 (441,732, 728,039)	465,835 (385,021, 616,443)	482,497 (397,621, 648,176)	1.3 (1.0, 2.0)	9.4 (6.6, 14.1)	14.7 (10.8, 20.6)	14.6 (11.1, 20.1)	16.3 (12.3, 22.4)	14.9 (12.0, 19.8)	13.3 (11.0, 17.5)	10.3 (8.5, 13.8)
Unintentional injuries	24,012 (18,108, 37,906)	82,012 (55,721, 127,146)	340,534 (238,445, 487,698)	414,115 (307,936, 577,425)	515,582 (388,476, 716,554)	523,000 (418,312, 703,119)	450,087 (368,381, 602,410)	470,290 (383,847, 634,799)	1.5 (1.1, 2.3)	10.1 (6.9, 15.7)	15.8 (11.1, 22.7)	16.0 (11.9, 22.4)	17.2 (13.0, 23.9)	15.3 (12.2, 20.5)	13.5 (11.0, 18.0)	10.3 (8.4, 14.0)
Road injury	24,012 (18,108, 37,906)	18,425 (14,662, 26,163)	80,421 (64,644, 110,767)	114,304 (92,974, 157,607)	163,469 (131,630, 228,049)	174,375 (142,217, 244,285)	151,644 (124,513, 213,356)	146,180 (120,141, 206,389)	27.7 (20.9, 43.7)	21.5 (17.1, 30.6)	25.5 (20.5, 35.1)	25.0 (20.4, 34.5)	25.9 (20.8, 36.1)	24.1 (19.7, 33.8)	23.6 (19.3, 33.1)	22.8 (18.7, 32.2)
Poisonings	0 (0, 0)	1,034 (576, 1,662)	3,586 (2,142, 5,444)	3,412 (2,092, 5,098)	3,312 (1,992, 4,965)	2,472 (1,592, 3,689)	1,655 (1,100, 2,450)	1,214 (835, 1,790)	0.0 (0.0, 0.0)	11.2 (6.2, 17.9)	16.3 (9.7, 24.7)	15.9 (9.7, 23.8)	16.4 (9.9, 24.6)	13.8 (8.9, 20.6)	11.9 (7.9, 17.7)	9.1 (6.3, 13.4)
Falls	0 (0, 0)	26,572 (15,044, 42,400)	111,082 (69,315, 165,369)	136,991 (89,134, 199,216)	171,185 (109,873, 250,726)	197,416 (134,936, 283,256)	186,463 (131,157, 265,953)	232,092 (164,185, 336,798)	0.0 (0.0, 0.0)	10.1 (5.7, 16.2)	16.1 (10.0, 23.9)	16.3 (10.6, 23.7)	16.9 (10.8, 24.7)	14.5 (9.9, 20.8)	11.9 (8.4, 17.0)	8.7 (6.1, 12.6)
Fire, heat and hot substances	0 (0, 0)	5,494 (3,016, 9,203)	22,746 (13,402, 35,277)	27,897 (17,283, 42,462)	28,838 (17,842, 43,694)	23,674 (16,029, 34,592)	16,175 (11,554, 22,999)	11,651 (8,720, 16,259)	0.0 (0.0, 0.0)	8.6 (4.7, 14.5)	13.6 (8.0, 21.1)	13.2 (8.2, 20.1)	13.8 (8.6, 20.9)	12.1 (8.2, 17.7)	11.3 (8.1, 16.1)	9.1 (6.8, 12.7)
Drowning	0 (0, 0)	432 (218, 739)	1,917 (1,020, 3,115)	2,049 (1,108, 3,308)	2,378 (1,263, 3,844)	1,779 (998, 2,891)	1,231 (731, 2,008)	1,135 (718, 1,876)	0.0 (0.0, 0.0)	9.2 (4.7, 15.8)	14.8 (7.9, 24.1)	14.2 (7.7, 22.9)	14.6 (7.8, 23.6)	11.1 (6.2, 18.0)	8.9 (5.3, 14.5)	6.4 (4.0, 10.5)

Cause of disease or injury	Alcohol-attributable YLD								Population Attributable Fraction							
	0 to 14	15 to 19	20 to 29	30 to 39	40 to 49	50 to 59	60 to 69	≥70	0 to 14	15 to 19	20 to 29	30 to 39	40 to 49	50 to 59	60 to 69	≥70
Exposure to mechanical forces	0 (0, 0)	5,820 (3,235, 9,520)	25,598 (15,460, 38,833)	31,513 (19,723, 46,911)	38,799 (23,776, 58,352)	35,010 (23,128, 51,541)	26,312 (18,130, 38,160)	20,386 (14,670, 29,236)	0.0 (0.0, 0.0)	10.3 (5.7, 16.8)	15.9 (9.6, 24.1)	15.5 (9.7, 23.1)	16.0 (9.8, 24.0)	13.6 (9.0, 20.1)	11.8 (8.1, 17.1)	8.9 (6.4, 12.8)
Other unintentional injuries	0 (0, 0)	24,234 (13,104, 41,750)	95,184 (53,828, 150,690)	97,949 (58,206, 153,115)	107,602 (63,519, 168,533)	88,274 (56,191, 134,559)	66,608 (44,870, 99,665)	57,632 (40,805, 85,649)	0.0 (0.0, 0.0)	7.5 (4.1, 12.9)	12.5 (7.0, 19.7)	12.0 (7.1, 18.8)	12.7 (7.5, 19.9)	10.5 (6.7, 16.0)	9.1 (6.1, 13.6)	6.9 (4.9, 10.2)
Intentional injuries	0 (0, 0)	10,474 (3,438, 20,482)	40,740 (14,138, 74,381)	35,852 (15,112, 62,755)	32,698 (14,118, 56,222)	23,317 (13,600, 36,270)	15,748 (10,260, 23,446)	12,207 (8,937, 17,572)	0.0 (0.0, 0.0)	6.1 (2.0, 12.0)	9.3 (3.2, 17.1)	7.3 (3.1, 12.8)	8.8 (3.8, 15.1)	9.3 (5.5, 14.5)	9.2 (6.0, 13.7)	8.1 (5.9, 11.7)
Self-harm	0 (0, 0)	863 (310, 1,529)	4,658 (1,864, 7,842)	5,461 (2,432, 9,036)	6,037 (2,621, 10,139)	5,253 (2,822, 8,242)	3,788 (2,169, 5,911)	3,123 (1,931, 4,945)	0.0 (0.0, 0.0)	9.9 (3.6, 17.6)	14.2 (5.7, 23.9)	13.9 (6.2, 23.0)	14.2 (6.2, 23.9)	12.5 (6.7, 19.6)	10.8 (6.2, 16.9)	8.3 (5.2, 13.2)
Interpersonal violence	0 (0, 0)	9,611 (3,117, 18,950)	36,082 (12,241, 66,704)	30,392 (12,831, 53,808)	26,661 (11,392, 46,183)	18,064 (10,817, 27,886)	11,959 (8,086, 17,545)	9,084 (6,964, 12,636)	0.0 (0.0, 0.0)	7.4 (2.4, 14.7)	11.4 (3.9, 21.0)	11.4 (4.8, 20.2)	12.7 (5.4, 22.0)	12.4 (7.4, 19.1)	11.9 (8.1, 17.5)	10.1 (7.7, 14.0)

Table A12. Global alcohol-attributable years lived with disability (YLD) by age and cause in 2016 and 95% uncertainty intervals

Cause of disease or injury	Alcohol-attributable YLD								Population Attributable Fraction							
	0 to 14	15 to 19	20 to 29	30 to 39	40 to 49	50 to 59	60 to 69	≥70	0 to 14	15 to 19	20 to 29	30 to 39	40 to 49	50 to 59	60 to 69	≥70
**All Causes**	121,857 (107,417, 156,196)	958,143 (894,498, 1,080,441)	5,276,204 (4,940,811, 5,703,154)	5,368,208 (4,958,737, 5,893,687)	4,817,591 (4,328,285, 5,445,044)	3,922,247 (3,467,709, 4,535,264)	2,562,622 (2,218,745, 3,095,008)	1,795,925 (1,498,919, 2,358,429)	0.1 (0.1, 0.2)	2.5 (2.3, 2.8)	5.5 (5.2, 6.0)	5.3 (4.9, 5.8)	4.5 (4.0, 5.1)	3.7 (3.3, 4.3)	2.7 (2.3, 3.3)	1.7 (1.4, 2.2)
**Communicable, maternal, perinatal and nutritional conditions**	0 (0, 0)	14,779 (6,775, 33,712)	129,202 (61,709, 213,468)	153,671 (82,249, 241,749)	151,104 (74,338, 242,164)	119,321 (48,955, 202,526)	90,632 (32,422, 164,662)	58,370 (21,761, 111,630)	0.0 (0.0, 0.0)	0.2 (0.1, 0.5)	0.8 (0.4, 1.3)	1.1 (0.6, 1.7)	1.4 (0.7, 2.2)	1.6 (0.7, 2.8)	1.9 (0.7, 3.5)	1.7 (0.6, 3.2)
Tuberculosis	0 (0, 0)	13,416 (5,128, 31,044)	105,185 (37,130, 186,411)	109,498 (37,364, 190,861)	113,897 (37,327, 198,357)	101,083 (30,494, 183,042)	82,364 (24,130, 155,537)	52,679 (16,028, 106,023)	0.0 (0.0, 0.0)	8.7 (3.3, 20.2)	22.1 (7.8, 39.2)	24.3 (8.3, 42.4)	25.2 (8.3, 43.9)	22.9 (6.9, 41.5)	20.4 (6.0, 38.5)	15.5 (4.7, 31.3)
HIV AIDS	0 (0, 0)	855 (644, 4,460)	22,098 (15,908, 34,960)	42,154 (30,224, 66,408)	34,691 (24,686, 53,525)	14,993 (10,694, 23,127)	4,966 (3,509, 8,194)	1,468 (1,039, 2,799)	0.0 (0.0, 0.0)	0.9 (0.7, 4.7)	3.0 (2.1, 4.7)	3.4 (2.4, 5.4)	3.7 (2.7, 5.8)	3.4 (2.4, 5.3)	2.9 (2.0, 4.8)	2.0 (1.4, 3.8)
Lower respiratory infections	0 (0, 0)	508 (325, 1,026)	1,919 (1,103, 3,060)	2,018 (1,189, 3,158)	2,516 (1,474, 3,912)	3,246 (2,036, 4,790)	3,301 (2,041, 4,971)	4,223 (2,523, 6,509)	0.0 (0.0, 0.0)	2.5 (1.6, 5.1)	6.0 (3.4, 9.5)	6.9 (4.0, 10.7)	7.3 (4.3, 11.4)	7.5 (4.7, 11.1)	6.4 (4.0, 9.6)	5.3 (3.1, 8.1)
**Noncommunicable diseases**	63,300 (63,300, 63,300)	667,830 (660,235, 693,324)	3,766,686 (3,723,644, 3,832,934)	3,447,087 (3,389,859, 3,534,366)	2,534,725 (2,435,634, 2,684,335)	1,731,623 (1,595,682, 1,932,088)	891,333 (740,027, 1,130,653)	459,809 (262,327, 808,057)	0.1 (0.1, 0.1)	2.3 (2.3, 2.4)	5.1 (5.1, 5.2)	4.3 (4.2, 4.4)	2.9 (2.8, 3.1)	1.9 (1.8, 2.2)	1.1 (0.9, 1.4)	0.5 (0.3, 0.8)
Malignant neoplasms	0 (0, 0)	0 (0, 0)	1,176 (1,012, 1,489)	10,780 (9,269, 12,571)	33,066 (28,607, 38,356)	66,968 (58,870, 75,947)	78,176 (68,919, 88,445)	81,116 (72,032, 93,159)	0.0 (0.0, 0.0)	0.0 (0.0, 0.0)	1.1 (0.9, 1.3)	4.3 (3.7, 5.0)	5.9 (5.1, 6.8)	6.7 (5.9, 7.6)	5.8 (5.1, 6.6)	4.5 (4.0, 5.2)
Lip and oral cavity cancer	0 (0, 0)	0 (0, 0)	292 (218, 402)	2,705 (2,098, 3,229)	5,752 (4,681, 6,648)	12,053 (10,159, 13,599)	12,391 (10,510, 14,066)	10,875 (9,164, 12,647)	100.0 (100.0, 100.0)	0.0 (0.0, 0.0)	7.7 (5.7, 10.6)	26.0 (20.2, 31.1)	30.9 (25.1, 35.7)	35.8 (30.2, 40.4)	34.1 (28.9, 38.7)	28.1 (23.7, 32.7)
Other pharynx cancers	0 (0, 0)	0 (0, 0)	55 (37, 79)	665 (493, 798)	2,783 (2,280, 3,178)	7,350 (6,316, 8,219)	7,159 (6,084, 8,103)	3,909 (3,257, 4,531)	100.0 (100.0, 100.0)	0.0 (0.0, 0.0)	7.7 (5.1, 10.9)	31.1 (23.1, 37.4)	38.3 (31.4, 43.7)	42.0 (36.1, 47.0)	39.5 (33.6, 44.7)	32.5 (27.1, 37.7)
Oesophagus cancer	0 (0, 0)	0 (0, 0)	25 (19, 35)	307 (258, 358)	1,783 (1,486, 2,061)	5,286 (4,407, 6,066)	8,077 (6,535, 9,494)	7,594 (6,110, 9,074)	100.0 (100.0, 100.0)	0.0 (0.0, 0.0)	4.3 (3.2, 5.9)	15.9 (13.3, 18.5)	21.2 (17.7, 24.5)	23.5 (19.6, 27.0)	22.1 (17.9, 26.0)	18.3 (14.7, 21.9)
Colon and rectum cancers	0 (0, 0)	0 (0, 0)	240 (200, 304)	1,591 (1,287, 1,946)	5,181 (4,141, 6,340)	13,119 (10,583, 15,874)	21,759 (17,477, 26,287)	32,543 (26,621, 39,720)	100.0 (100.0, 100.0)	0.0 (0.0, 0.0)	4.6 (3.9, 5.9)	10.2 (8.3, 12.5)	11.5 (9.2, 14.1)	12.9 (10.4, 15.6)	13.3 (10.7, 16.1)	12.2 (10.0, 14.8)
Liver cancer	0 (0, 0)	0 (0, 0)	186 (109, 314)	1,094 (605, 1,659)	3,650 (1,929, 5,705)	6,757 (3,689, 10,197)	7,924 (4,550, 11,748)	8,781 (5,378, 12,907)	0.0 (0.0, 0.0)	0.0 (0.0, 0.0)	5.5 (3.2, 9.3)	11.1 (6.1, 16.8)	11.8 (6.2, 18.5)	12.6 (6.9, 19.0)	12.6 (7.2, 18.7)	13.2 (8.1, 19.4)
Breast cancer	0 (0, 0)	0 (0, 0)	354 (284, 467)	4,158 (3,362, 5,215)	12,458 (10,155, 15,613)	17,357 (14,151, 21,676)	14,562 (12,005, 18,133)	12,808 (10,498, 16,119)	100.0 (100.0, 100.0)	0.0 (0.0, 0.0)	3.5 (2.8, 4.6)	8.3 (6.7, 10.4)	8.7 (7.1, 11.0)	9.4 (7.6, 11.7)	8.5 (7.0, 10.6)	7.2 (5.9, 9.0)
Larynx cancer	0 (0, 0)	0 (0, 0)	23 (17, 33)	260 (198, 327)	1,460 (1,132, 1,803)	5,047 (3,980, 6,178)	6,305 (4,936, 7,755)	4,606 (3,524, 5,767)	100.0 (100.0, 100.0)	0.0 (0.0, 0.0)	5.3 (3.7, 7.6)	17.8 (13.6, 22.4)	22.4 (17.4, 27.7)	25.6 (20.2, 31.3)	24.8 (19.4, 30.5)	21.5 (16.5, 27.0)
Diabetes mellitus	0 (0, 0)	-3,784 (-6,593, 1,560)	-49,800 (-74,506, -17,358)	-93,082 (-142,057, -26,687)	-180,472 (-274,878, -53,311)	-208,058 (-327,435, -49,177)	-183,792 (-298,027, -35,772)	-152,927 (-257,487, -27,640)	0.0 (0.0, 0.0)	-1.4 (-2.4, 0.6)	-3.3 (-4.9, -1.1)	-3.1 (-4.7, -0.9)	-3.4 (-5.2, -1.0)	-3.1 (-4.8, -0.7)	-3.0 (-4.8, -0.6)	-2.9 (-4.8, -0.5)
Alcohol use disorders	63,300 (63,300, 63,300)	607,482 (607,482, 607,482)	3,565,027 (3,565,027, 3,565,027)	3,299,508 (3,299,508, 3,299,508)	2,421,999 (2,421,999, 2,421,999)	1,609,274 (1,609,274, 1,609,274)	777,974 (777,974, 777,974)	320,465 (320,465, 320,465)	100.0 (100.0, 100.0)	100.0 (100.0, 100.0)	100.0 (100.0, 100.0)	100.0 (100.0, 100.0)	100.0 (100.0, 100.0)	100.0 (100.0, 100.0)	100.0 (100.0, 100.0)	100.0 (100.0, 100.0)

Cause of disease or injury	Alcohol-attributable YLD								Population Attributable Fraction							
	0 to 14	15 to 19	20 to 29	30 to 39	40 to 49	50 to 59	60 to 69	≥70	0 to 14	15 to 19	20 to 29	30 to 39	40 to 49	50 to 59	60 to 69	≥70
Epilepsy	0 (0, 0)	40,675 (33,254, 58,559)	173,059 (139,540, 212,755)	136,691 (110,440, 166,593)	119,795 (96,520, 145,158)	95,483 (76,781, 116,621)	68,831 (55,307, 84,871)	57,051 (46,322, 70,559)	0.0 (0.0, 0.0)	5.8 (4.7, 8.3)	13.5 (10.9, 16.6)	14.6 (11.8, 17.8)	15.6 (12.6, 18.9)	14.6 (11.8, 17.9)	13.4 (10.7, 16.5)	11.4 (9.2, 14.0)
Cardiovascular diseases	0 (0, 0)	1,007 (406, 4,637)	4,913 (-1,364, 28,134)	9,998 (-3,745, 38,729)	9,640 (-24,932, 60,287)	8,140 (-61,521, 104,466)	20,331 (-84,443, 181,019)	63,058 (-114,192, 369,324)	0.0 (0.0, 0.0)	0.2 (0.1, 0.9)	0.4 (-0.1, 2.2)	0.6 (-0.2, 2.3)	0.3 (-0.8, 2.0)	0.2 (-1.2, 2.0)	0.3 (-1.1, 2.3)	0.5 (-0.9, 2.8)
Hypertensive heart disease	0 (0, 0)	0 (0, 1)	10 (7, 14)	4,008 (3,014, 5,384)	12,057 (8,975, 16,098)	18,968 (14,511, 24,522)	25,544 (19,576, 32,859)	42,098 (32,111, 56,251)	0.0 (0.0, 0.0)	4.1 (2.9, 11.6)	10.8 (8.1, 15.2)	8.1 (6.1, 10.8)	8.7 (6.5, 11.6)	8.6 (6.5, 11.1)	8.0 (6.1, 10.3)	6.5 (5.0, 8.7)
Ischaemic heart disease	0 (0, 0)	393 (-235, 1,405)	1,637 (-4,570, 8,921)	3,368 (-9,376, 15,952)	4,349 (-19,718, 24,420)	13,703 (-24,678, 46,159)	27,197 (-28,182, 76,932)	65,265 (-39,550, 160,407)	0.0 (0.0, 0.0)	1.1 (-0.6, 3.8)	0.9 (-2.7, 5.2)	0.9 (-2.4, 4.1)	0.6 (-2.6, 3.3)	1.1 (-2.0, 3.7)	1.6 (-1.7, 4.6)	2.5 (-1.5, 6.2)
Ischaemic stroke	0 (0, 0)	-1,133 (-1,439, 2,015)	-7,019 (-9,857, 15,106)	-13,964 (-20,251, 9,763)	-44,360 (-67,399, -6,232)	-90,249 (-147,656, -11,333)	-115,306 (-206,051, 28,426)	-152,937 (-315,856, 138,736)	0.0 (0.0, 0.0)	-2.3 (-2.9, 4.0)	-3.6 (-5.0, 7.7)	-3.9 (-5.7, 2.7)	-4.8 (-7.3, -0.7)	-4.4 (-7.3, -0.6)	-3.6 (-6.5, 0.9)	-3.0 (-6.3, 2.8)
Haemorrhagic stroke	0 (0, 0)	1,490 (1,059, 2,205)	9,138 (6,481, 12,615)	15,125 (10,928, 20,647)	35,351 (25,383, 48,283)	61,313 (44,490, 82,654)	78,606 (57,201, 106,929)	103,836 (75,725, 140,838)	0.0 (0.0, 0.0)	7.4 (5.3, 11.0)	12.5 (8.8, 17.2)	12.7 (9.2, 17.4)	13.4 (9.6, 18.3)	12.5 (9.1, 16.8)	11.4 (8.3, 15.5)	10.9 (7.9, 14.7)
Cardiomyopathy, myocarditis, endocarditis	0 (0, 0)	257 (240, 393)	1,147 (1,076, 1,584)	1,461 (1,415, 1,836)	2,243 (2,190, 2,818)	4,406 (4,331, 5,282)	4,289 (4,223, 5,136)	4,797 (4,752, 5,248)	0.0 (0.0, 0.0)	1.3 (1.2, 2.0)	1.5 (1.4, 2.1)	2.7 (2.6, 3.4)	3.7 (3.6, 4.7)	5.1 (5.0, 6.1)	4.4 (4.3, 5.3)	2.8 (2.7, 3.0)
Digestive diseases	0 (0, 0)	22,450 (18,550, 28,590)	72,312 (62,519, 81,605)	83,192 (72,498, 92,734)	130,698 (112,961, 145,492)	159,817 (139,099, 177,829)	129,814 (112,966, 146,046)	91,045 (79,718, 103,574)	0.0 (0.0, 0.0)	8.9 (7.3, 11.3)	11.0 (9.5, 12.4)	8.1 (7.0, 9.0)	8.2 (7.0, 9.1)	10.0 (8.7, 11.2)	9.1 (7.9, 10.2)	6.9 (6.0, 7.8)
Cirrhosis of the liver	0 (0, 0)	22,287 (18,391, 28,434)	71,078 (61,255, 80,182)	81,046 (70,382, 90,477)	127,689 (110,159, 142,065)	156,380 (135,887, 173,962)	126,648 (109,915, 142,318)	88,052 (76,751, 100,300)	0.0 (0.0, 0.0)	29.1 (24.0, 37.2)	48.0 (41.4, 54.2)	52.0 (45.1, 58.0)	55.6 (48.0, 61.9)	54.0 (46.9, 60.1)	51.0 (44.2, 57.3)	46.2 (40.3, 52.6)
Pancreatitis	0 (0, 0)	163 (98, 275)	1,233 (828, 1,827)	2,146 (1,481, 3,034)	3,009 (1,940, 4,527)	3,437 (2,353, 4,963)	3,166 (2,201, 4,590)	2,993 (2,092, 4,346)	0.0 (0.0, 0.0)	12.6 (7.6, 21.2)	24.7 (16.6, 36.6)	26.6 (18.4, 37.7)	23.9 (15.4, 36.0)	23.8 (16.3, 34.3)	22.3 (15.5, 32.3)	20.2 (14.1, 29.4)
**Injuries**	58,557 (44,117, 92,896)	275,535 (209,843, 382,089)	1,380,317 (1,059,021, 1,767,436)	1,767,451 (1,375,752, 2,239,499)	2,131,761 (1,675,753, 2,689,397)	2,071,303 (1,649,540, 2,621,702)	1,580,658 (1,265,646, 2,002,629)	1,277,746 (1,023,597, 1,652,199)	1.4 (1.1, 2.3)	12.1 (9.2, 16.7)	21.4 (16.4, 27.4)	22.1 (17.2, 28.0)	23.7 (18.6, 29.9)	22.0 (17.6, 27.9)	19.5 (15.6, 24.7)	14.6 (11.7, 18.9)
Unintentional injuries	58,557 (44,117, 92,896)	251,462 (183,332, 359,797)	1,255,705 (937,119, 1,631,606)	1,633,150 (1,249,600, 2,100,703)	1,994,307 (1,542,877, 2,554,526)	1,959,820 (1,534,684, 2,513,282)	1,511,508 (1,199,039, 1,942,448)	1,236,551 (985,839, 1,612,257)	1.6 (1.2, 2.5)	12.9 (9.4, 18.4)	22.9 (17.1, 29.7)	23.9 (18.3, 30.8)	25.1 (19.5, 32.2)	23.1 (18.1, 29.6)	20.0 (15.9, 25.7)	14.8 (11.8, 19.3)
Road injury	58,557 (44,117, 92,896)	55,599 (40,192, 82,917)	301,514 (219,411, 424,300)	445,530 (325,956, 624,565)	603,241 (443,810, 846,149)	604,103 (449,338, 858,666)	464,111 (346,892, 665,722)	356,716 (267,406, 519,150)	26.9 (20.3, 42.7)	22.4 (16.2, 33.4)	29.9 (21.8, 42.1)	30.2 (22.1, 42.4)	31.3 (23.0, 43.8)	29.6 (22.0, 42.1)	28.0 (20.9, 40.2)	25.5 (19.1, 37.1)
Poisonings	0 (0, 0)	2,883 (1,892, 4,204)	11,218 (7,600, 15,002)	10,999 (7,520, 14,663)	10,634 (7,232, 14,227)	8,250 (5,674, 11,065)	5,294 (3,669, 7,164)	3,361 (2,362, 4,645)	0.0 (0.0, 0.0)	13.6 (8.9, 19.8)	22.5 (15.2, 30.1)	22.9 (15.7, 30.5)	23.7 (16.1, 31.7)	21.2 (14.6, 28.5)	18.4 (12.7, 24.9)	13.7 (9.6, 18.9)
Falls	0 (0, 0)	83,559 (54,835, 122,875)	406,337 (273,435, 543,675)	532,478 (364,249, 706,375)	650,622 (445,333, 859,897)	703,382 (496,973, 924,807)	590,910 (427,145, 782,532)	568,744 (417,025, 765,645)	0.0 (0.0, 0.0)	13.4 (8.8, 19.7)	23.5 (15.8, 31.5)	24.6 (16.8, 32.6)	25.2 (17.2, 33.3)	22.4 (15.8, 29.5)	18.4 (13.3, 24.4)	12.8 (9.4, 17.3)
Fire, heat and hot substances	0 (0, 0)	14,997 (9,832, 22,628)	69,999 (47,415, 94,917)	89,063 (61,487, 119,529)	91,487 (63,114, 122,356)	77,240 (54,803, 102,429)	49,646 (35,872, 65,758)	31,334 (22,905, 42,064)	0.0 (0.0, 0.0)	11.2 (7.4, 16.9)	20.3 (13.7, 27.5)	20.7 (14.3, 27.8)	21.6 (14.9, 28.9)	19.9 (14.1, 26.3)	17.9 (12.9, 23.7)	13.8 (10.1, 18.5)

Cause of disease or injury	Alcohol-attributable YLD								Population Attributable Fraction							
	0 to 14	15 to 19	20 to 29	30 to 39	40 to 49	50 to 59	60 to 69	≥70	0 to 14	15 to 19	20 to 29	30 to 39	40 to 49	50 to 59	60 to 69	≥70
Drowning	0 (0, 0)	1,201 (767, 1,833)	5,972 (3,909, 8,223)	6,876 (4,548, 9,454)	7,911 (5,314, 10,844)	6,680 (4,487, 9,133)	4,692 (3,184, 6,502)	5,765 (3,609, 8,356)	0.0 (0.0, 0.0)	11.3 (7.2, 17.2)	20.7 (13.6, 28.6)	21.1 (13.9, 29.0)	21.7 (14.6, 29.8)	18.8 (12.6, 25.7)	15.8 (10.7, 21.9)	14.1 (8.9, 20.5)
Exposure to mechanical forces	0 (0, 0)	22,212 (13,999, 33,164)	124,854 (79,796, 170,037)	167,901 (108,588, 227,433)	206,545 (133,859, 279,364)	192,821 (126,345, 262,784)	135,113 (89,880, 185,620)	85,179 (57,715, 119,556)	0.0 (0.0, 0.0)	13.8 (8.7, 20.6)	24.7 (15.8, 33.7)	25.4 (16.4, 34.4)	26.2 (17.0, 35.5)	24.0 (15.7, 32.7)	20.7 (13.7, 28.4)	15.2 (10.3, 21.3)
Other unintentional injuries	0 (0, 0)	71,010 (45,433, 109,642)	335,812 (217,224, 466,854)	380,302 (248,471, 527,330)	423,867 (275,379, 582,576)	367,344 (241,342, 505,333)	261,742 (176,696, 360,782)	185,453 (128,371, 261,472)	0.0 (0.0, 0.0)	9.6 (6.2, 14.9)	18.8 (12.1, 26.1)	19.4 (12.7, 26.9)	20.5 (13.3, 28.1)	18.2 (12.0, 25.0)	15.6 (10.6, 21.6)	11.2 (7.8, 15.8)
Intentional injuries	0 (0, 0)	24,073 (12,257, 41,461)	124,612 (60,056, 190,658)	134,301 (66,392, 200,438)	137,454 (65,909, 205,660)	111,482 (56,538, 166,995)	69,150 (37,476, 102,772)	41,195 (25,078, 61,051)	0.0 (0.0, 0.0)	7.2 (3.7, 12.5)	13.2 (6.3, 20.2)	11.6 (5.7, 17.3)	12.8 (6.1, 19.2)	12.5 (6.3, 18.7)	12.2 (6.6, 18.2)	10.8 (6.6, 16.0)
Self-harm	0 (0, 0)	1,940 (1,018, 3,202)	12,327 (6,332, 18,490)	16,241 (8,608, 24,003)	18,879 (9,826, 27,954)	17,804 (9,937, 25,856)	13,044 (7,418, 19,005)	10,542 (6,020, 15,856)	0.0 (0.0, 0.0)	10.8 (5.7, 17.9)	18.5 (9.5, 27.8)	19.2 (10.2, 28.4)	20.0 (10.4, 29.6)	18.7 (10.4, 27.2)	16.5 (9.4, 24.0)	13.1 (7.5, 19.7)
Interpersonal violence	0 (0, 0)	22,133 (11,242, 38,508)	112,285 (53,798, 172,047)	118,060 (57,608, 176,926)	118,575 (56,011, 178,374)	93,678 (46,232, 141,089)	56,106 (29,894, 84,067)	30,653 (18,970, 45,384)	0.0 (0.0, 0.0)	9.1 (4.6, 15.9)	17.3 (8.3, 26.5)	19.0 (9.3, 28.4)	20.8 (9.8, 31.3)	20.4 (10.1, 30.7)	18.2 (9.7, 27.3)	13.5 (8.4, 20.0)

Table A13. Global alcohol-attributable premature years lived with disability (YLD) by cause and sex in 2016 and 95% uncertainty intervals

Cause of disease or injury	Men		Women		Total	
	Alcohol-attributable burden	PAF (%)	Alcohol-attributable burden	PAF (%)	Alcohol-attributable burden	PAF (%)
**All Causes**	17,060,843 (14,983,359, 19,491,704)	5.6 (5.0, 6.4)	5,966,030 (5,321,094, 7,199,212)	1.8 (1.6, 2.2)	23,026,873 (20,916,202, 25,908,794)	3.7 (3.3, 4.1)
**Communicable, maternal, perinatal and nutritional conditions**	518,412 (175,380, 909,154)	1.2 (0.4, 2.0)	140,296 (59,048, 317,534)	0.3 (0.1, 0.6)	658,708 (306,448, 1,098,281)	0.7 (0.3, 1.1)
Tuberculosis	426,678 (83,301, 813,969)	26.6 (5.2, 50.7)	98,766 (15,700, 248,420)	11.1 (1.8, 27.8)	525,444 (171,573, 945,251)	21.0 (6.9, 37.8)
HIV AIDS	82,706 (50,373, 124,975)	4.0 (2.4, 6.1)	37,050 (22,549, 89,829)	2.0 (1.2, 4.7)	119,756 (85,666, 190,674)	3.0 (2.2, 4.8)
Lower respiratory infections	9,028 (3,782, 15,411)	4.4 (1.8, 7.5)	4,480 (2,701, 7,455)	2.3 (1.4, 3.8)	13,508 (8,169, 20,917)	3.4 (2.0, 5.2)
**Noncommunicable diseases**	9,785,020 (9,460,887, 10,201,601)	4.3 (4.1, 4.5)	3,317,564 (2,928,021, 3,897,395)	1.3 (1.1, 1.5)	13,102,584 (12,608,380, 13,871,000)	2.7 (2.6, 2.9)
Malignant neoplasms	118,920 (102,048, 136,388)	7.2 (6.2, 8.2)	71,246 (60,880, 86,110)	4.1 (3.5, 5.0)	190,166 (166,675, 216,809)	5.6 (4.9, 6.4)
Lip and oral cavity cancer	26,777 (22,185, 30,477)	40.8 (33.8, 46.4)	6,415 (4,779, 8,450)	17.1 (12.7, 22.5)	33,192 (27,666, 37,944)	32.1 (26.8, 36.7)
Other pharynx cancers	15,911 (13,380, 17,948)	45.4 (38.2, 51.2)	2,100 (1,525, 2,782)	19.4 (14.1, 25.7)	18,011 (15,210, 20,377)	39.3 (33.2, 44.4)
Oesophagus cancer	13,689 (11,156, 15,864)	26.3 (21.4, 30.5)	1,789 (1,314, 2,402)	10.0 (7.3, 13.4)	15,478 (12,705, 18,014)	22.1 (18.2, 25.7)
Colon and rectum cancers	34,287 (26,638, 41,752)	17.7 (13.7, 21.5)	7,603 (4,478, 11,478)	5.5 (3.3, 8.4)	41,890 (33,688, 50,750)	12.6 (10.2, 15.3)
Liver cancer	15,943 (7,616, 25,162)	12.7 (6.1, 20.1)	3,668 (1,883, 6,159)	10.1 (5.2, 17.0)	19,610 (10,883, 29,624)	12.1 (6.7, 18.3)
Breast cancer	0 (0, 0)	0.0 (0.0, 0.0)	48,889 (39,957, 61,104)	8.8 (7.2, 11.0)	48,889 (39,957, 61,104)	8.7 (7.1, 10.9)
Larynx cancer	12,313 (9,428, 15,260)	26.4 (20.2, 32.7)	781 (522, 1,150)	11.1 (7.4, 16.4)	13,095 (10,263, 16,096)	24.4 (19.1, 30.0)
Diabetes mellitus	232,970 (36,820, 524,073)	1.9 (0.3, 4.2)	-951,959 (-1,318,608, -541,465)	-8.7 (-12.1, -5.0)	-718,988 (-1,123,496, -180,745)	-3.1 (-4.8, -0.8)
Alcohol use disorders	8,502,999 (8,502,999, 8,502,999)	100.0 (100.0, 100.0)	3,841,564 (3,841,564, 3,841,564)	100.0 (100.0, 100.0)	12,344,562 (12,344,562, 12,344,562)	100.0 (100.0, 100.0)
Epilepsy	444,875 (334,040, 562,373)	12.1 (9.1, 15.3)	189,659 (146,499, 264,022)	5.6 (4.3, 7.8)	634,534 (511,841, 784,556)	9.0 (7.3, 11.1)
Cardiovascular diseases	89,875 (-44,390, 229,835)	0.9 (-0.4, 2.3)	-35,846 (-214,304, 277,094)	-0.4 (-2.1, 2.7)	54,029 (-175,599, 417,273)	0.3 (-0.9, 2.1)
Hypertensive heart disease	45,112 (34,102, 54,916)	15.7 (11.9, 19.1)	15,476 (6,915, 30,215)	3.5 (1.6, 6.8)	60,588 (46,083, 78,878)	8.3 (6.3, 10.8)
Ischaemic heart disease	-9,400 (-106,834, 52,016)	-0.4 (-4.4, 2.1)	60,047 (-34,238, 173,683)	3.2 (-1.8, 9.3)	50,648 (-86,759, 173,789)	1.2 (-2.0, 4.0)
Ischaemic stroke	-57,267 (-138,091, 64,117)	-1.8 (-4.4, 2.0)	-214,764 (-373,429, 40,946)	-6.0 (-10.4, 1.1)	-272,032 (-452,653, 37,744)	-4.0 (-6.7, 0.6)
Haemorrhagic stroke	101,423 (67,753, 138,229)	12.4 (8.3, 16.9)	99,599 (59,926, 158,251)	11.4 (6.8, 18.1)	201,022 (145,543, 273,334)	11.9 (8.6, 16.1)
Cardiomyopathy, myocarditis, endocarditis	10,006 (9,676, 13,246)	4.8 (4.6, 6.3)	3,797 (3,796, 3,825)	1.8 (1.8, 1.8)	13,803 (13,475, 17,049)	3.2 (3.2, 4.0)
Digestive diseases	395,382 (324,228, 456,542)	11.3 (9.2, 13.0)	202,900 (174,573, 234,164)	5.0 (4.3, 5.8)	598,282 (518,592, 672,295)	7.9 (6.9, 8.9)
Cirrhosis of the liver	385,269 (314,838, 446,306)	44.4 (36.3, 51.4)	199,859 (172,171, 229,323)	38.9 (33.5, 44.6)	585,128 (505,990, 657,436)	42.3 (36.6, 47.6)
Pancreatitis	10,113 (6,694, 13,482)	37.7 (25.0, 50.3)	3,040 (296, 8,172)	9.8 (1.0, 26.3)	13,154 (8,902, 19,217)	22.7 (15.4, 33.2)
**Injuries**	6,757,411 (4,814,198, 8,997,209)	23.9 (17.1, 31.9)	2,508,171 (1,946,346, 3,424,219)	13.2 (10.2, 18.0)	9,265,581 (7,279,672, 11,795,649)	19.6 (15.4, 24.9)
Unintentional injuries	6,315,167 (4,393,773, 8,579,603)	25.3 (17.6, 34.4)	2,349,342 (1,795,379, 3,252,259)	13.9 (10.6, 19.2)	8,664,509 (6,690,767, 11,195,258)	20.7 (16.0, 26.7)
Road injury	1,806,006 (1,254,879, 2,608,994)	32.1 (22.3, 46.3)	726,650 (588,748, 1,018,132)	24.7 (20.0, 34.6)	2,532,656 (1,869,716, 3,595,215)	29.5 (21.8, 41.9)
Poisonings	33,806 (19,121, 48,760)	22.4 (12.7, 32.3)	15,472 (9,493, 23,308)	12.6 (7.7, 19.0)	49,278 (33,587, 66,326)	18.0 (12.3, 24.2)
Falls	2,137,579 (1,275,129, 3,021,437)	25.8 (15.4, 36.5)	829,709 (549,458, 1,206,920)	13.3 (8.8, 19.4)	2,967,288 (2,061,970, 3,940,162)	20.5 (14.2, 27.2)
Fire, heat and hot substances	267,608 (154,422, 384,248)	23.6 (13.6, 33.9)	124,824 (79,125, 188,226)	11.4 (7.2, 17.2)	392,432 (272,523, 527,615)	17.6 (12.2, 23.7)
Drowning	23,546 (13,075, 34,602)	22.3 (12.4, 32.7)	9,785 (5,338, 15,906)	11.3 (6.2, 18.4)	33,332 (22,209, 45,988)	17.3 (11.5, 23.9)
Exposure to mechanical forces	686,395 (396,354, 981,668)	26.4 (15.3, 37.8)	163,051 (103,452, 243,318)	13.0 (8.3, 19.4)	849,446 (552,467, 1,158,402)	22.1 (14.4, 30.1)
Other unintentional injuries	1,360,227 (746,551, 2,020,408)	19.7 (10.8, 29.2)	479,851 (289,719, 748,312)	9.4 (5.6, 14.6)	1,840,078 (1,204,545, 2,552,518)	15.3 (10.0, 21.2)
Intentional injuries	442,244 (142,043, 716,283)	13.6 (4.4, 22.0)	158,829 (70,666, 273,556)	7.5 (3.3, 12.9)	601,072 (298,629, 907,983)	11.2 (5.6, 16.9)
Self-harm	54,175 (19,027, 86,614)	22.3 (7.8, 35.7)	26,060 (12,218, 42,699)	12.7 (6.0, 20.9)	80,235 (43,138, 118,509)	17.9 (9.6, 26.5)
Interpersonal violence	388,069 (123,852, 629,819)	21.5 (6.9, 34.9)	132,768 (58,484, 231,075)	10.0 (4.4, 17.5)	520,837 (254,784, 791,011)	16.7 (8.1, 25.3)

PAF: Population Attributable Fraction

Table A14. Global alcohol-attributable disability adjusted life years (DALYs) lost by age and cause in 2016 among men and 95% uncertainty intervals

Cause of disease or injury	Alcohol-attributable DALYs lost								Population attributable fraction (%)							
	0 to 14	15 to 19	20 to 29	30 to 39	40 to 49	50 to 59	60 to 69	≥70	0 to 14	15 to 19	20 to 29	30 to 39	40 to 49	50 to 59	60 to 69	≥70
**All Causes**	1,794,719 (1,323,529, 2,933,116)	3,272,942 (2,631,977, 4,550,980)	17,180,017 (13,951,679, 21,111,719)	18,666,386 (15,734,503, 21,964,853)	18,585,694 (15,847,610, 21,526,787)	17,950,750 (15,548,588, 20,573,066)	13,428,669 (11,340,799, 15,768,019)	8,934,944 (6,529,076, 11,699,483)	0.5 (0.4, 0.8)	6.8 (5.5, 9.4)	13.9 (11.3, 17.1)	14.2 (12.0, 16.7)	12.7 (10.9, 14.7)	10.1 (8.8, 11.6)	6.9 (5.8, 8.1)	3.9 (2.9, 5.1)
**Communicable, maternal, perinatal and nutritional conditions**	0 (0, 0)	147,272 (61,852, 380,950)	1,572,577 (548,040, 2,670,501)	2,362,062 (1,003,711, 3,738,583)	2,414,133 (1,001,819, 3,872,554)	2,019,796 (778,291, 3,400,857)	1,392,323 (542,409, 2,470,335)	1,182,144 (498,086, 2,097,253)	0.0 (0.0, 0.0)	1.5 (0.6, 3.9)	7.1 (2.5, 12.0)	8.7 (3.7, 13.7)	10.0 (4.1, 16.0)	10.8 (4.1, 18.1)	9.0 (3.5, 16.0)	6.4 (2.7, 11.4)
Tuberculosis	0 (0, 0)	112,506 (22,853, 295,494)	1,265,199 (264,853, 2,331,400)	1,691,015 (363,708, 2,996,469)	1,773,137 (367,091, 3,165,893)	1,564,994 (331,436, 2,868,923)	1,033,106 (202,187, 2,024,364)	657,251 (118,681, 1,366,337)	0.0 (0.0, 0.0)	10.3 (2.1, 27.1)	27.0 (5.6, 49.7)	30.1 (6.5, 53.3)	30.3 (6.3, 54.1)	27.1 (5.7, 49.7)	22.9 (4.5, 44.9)	17.9 (3.2, 37.2)
HIV AIDS	0 (0, 0)	11,691 (7,090, 64,799)	152,515 (89,191, 235,980)	459,902 (269,819, 696,689)	405,879 (241,629, 608,519)	151,316 (90,663, 225,149)	35,557 (21,124, 53,104)	6,119 (3,513, 9,503)	0.0 (0.0, 0.0)	1.0 (0.6, 5.6)	3.8 (2.2, 5.8)	4.4 (2.6, 6.7)	4.7 (2.8, 7.1)	4.4 (2.6, 6.5)	4.0 (2.4, 6.0)	3.0 (1.7, 4.6)
Lower respiratory infections	0 (0, 0)	23,076 (6,651, 66,471)	154,863 (49,131, 281,481)	211,145 (98,639, 341,178)	235,117 (103,137, 387,470)	303,486 (131,200, 502,739)	323,660 (109,964, 585,964)	518,774 (134,794, 1,010,336)	0.0 (0.0, 0.0)	2.5 (0.7, 7.3)	7.6 (2.4, 13.9)	10.5 (4.9, 16.9)	10.5 (4.6, 17.3)	9.6 (4.2, 15.9)	7.4 (2.5, 13.5)	6.1 (1.6, 11.8)
**Noncommunicable diseases**	40,056 (40,056, 40,056)	693,213 (643,082, 799,390)	4,562,656 (4,236,431, 4,828,871)	7,324,123 (6,627,359, 7,829,704)	9,266,087 (8,254,354, 9,989,911)	10,694,661 (9,391,190, 11,746,762)	8,795,141 (7,214,509, 10,277,680)	5,773,055 (3,671,070, 8,113,590)	0.1 (0.1, 0.1)	3.2 (3.0, 3.7)	8.1 (7.6, 8.6)	10.4 (9.4, 11.1)	9.5 (8.5, 10.3)	7.7 (6.7, 8.4)	5.3 (4.4, 6.2)	2.9 (1.8, 4.1)
Malignant neoplasms	0 (0, 0)	0 (0, 0)	55,985 (41,727, 81,686)	435,287 (333,067, 541,753)	1,275,149 (1,006,995, 1,559,400)	2,555,817 (2,125,265, 2,996,003)	2,671,021 (2,238,918, 3,109,202)	1,987,684 (1,661,628, 2,375,098)	0.0 (0.0, 0.0)	0.0 (0.0, 0.0)	1.3 (1.0, 1.9)	6.4 (4.9, 7.9)	8.1 (6.4, 9.9)	8.5 (7.0, 9.9)	7.2 (6.0, 8.4)	5.4 (4.5, 6.4)
Lip and oral cavity cancer	0 (0, 0)	0 (0, 0)	12,074 (7,055, 18,800)	124,643 (86,785, 153,101)	268,333 (205,140, 314,703)	445,095 (364,084, 505,565)	366,904 (304,378, 419,217)	181,052 (151,601, 208,434)	0.0 (0.0, 0.0)	0.0 (0.0, 0.0)	8.4 (4.9, 13.1)	34.7 (24.2, 42.6)	38.3 (29.3, 44.9)	42.2 (34.5, 47.9)	41.7 (34.6, 47.6)	37.4 (31.3, 43.0)
Other pharynx cancers	0 (0, 0)	0 (0, 0)	3,016 (1,887, 4,629)	46,327 (33,553, 56,381)	180,224 (139,855, 210,242)	385,054 (317,931, 435,730)	326,638 (271,881, 372,496)	150,309 (123,750, 174,890)	0.0 (0.0, 0.0)	0.0 (0.0, 0.0)	8.5 (5.3, 13.0)	33.7 (24.4, 41.0)	39.0 (30.3, 45.5)	43.7 (36.1, 49.4)	42.4 (35.3, 48.3)	35.0 (28.8, 40.8)
Oesophagus cancer	0 (0, 0)	0 (0, 0)	2,399 (1,707, 3,564)	30,270 (24,741, 36,131)	182,531 (152,944, 210,911)	558,162 (464,199, 640,248)	687,938 (558,364, 801,111)	470,513 (371,784, 569,296)	0.0 (0.0, 0.0)	0.0 (0.0, 0.0)	4.4 (3.1, 6.6)	15.8 (12.9, 18.9)	22.7 (19.0, 26.2)	25.9 (21.5, 29.7)	25.6 (20.8, 29.8)	22.5 (17.8, 27.2)
Colon and rectum cancers	0 (0, 0)	0 (0, 0)	13,294 (11,161, 16,172)	77,288 (61,304, 94,314)	181,840 (143,223, 220,997)	388,141 (307,143, 471,459)	537,557 (427,837, 646,242)	630,699 (511,498, 755,515)	0.0 (0.0, 0.0)	0.0 (0.0, 0.0)	5.7 (4.8, 7.0)	13.9 (11.0, 17.0)	15.4 (12.2, 18.8)	17.2 (13.6, 20.9)	18.0 (14.3, 21.6)	18.2 (14.7, 21.8)
Liver cancer	0 (0, 0)	0 (0, 0)	24,512 (12,433, 45,658)	146,126 (68,309, 229,951)	398,611 (184,474, 633,590)	607,234 (294,239, 947,037)	564,440 (278,586, 878,484)	441,375 (219,282, 714,085)	0.0 (0.0, 0.0)	0.0 (0.0, 0.0)	5.3 (2.7, 9.8)	11.8 (5.5, 18.5)	12.3 (5.7, 19.5)	13.0 (6.3, 20.3)	13.3 (6.6, 20.7)	13.2 (6.6, 21.4)
Breast cancer	0 (0, 0)	0 (0, 0)	0 (0, 0)	0 (0, 0)	0 (0, 0)	0 (0, 0)	0 (0, 0)	0 (0, 0)	0.0 (0.0, 0.0)	0.0 (0.0, 0.0)	0.0 (0.0, 1.4)	0.0 (0.0, 0.0)	0.0 (0.0, 0.0)	0.0 (0.0, 0.0)	0.0 (0.0, 0.0)	0.0 (0.0, 0.0)
Larynx cancer	0 (0, 0)	0 (0, 0)	689 (430, 1,095)	10,633 (7,667, 13,674)	63,610 (47,213, 80,445)	172,130 (131,958, 213,482)	187,545 (142,368, 233,398)	113,737 (84,723, 144,355)	0.0 (0.0, 0.0)	0.0 (0.0, 0.0)	4.6 (2.9, 7.3)	20.2 (14.6, 26.0)	23.1 (17.1, 29.2)	26.1 (20.0, 32.3)	25.7 (19.5, 32.0)	22.6 (16.9, 28.7)
Diabetes mellitus	0 (0, 0)	3,001 (-254, 10,489)	20,436 (4,243, 45,207)	43,049 (7,932, 97,408)	90,509 (14,923, 208,374)	143,675 (16,666, 329,540)	160,368 (11,765, 372,893)	160,946 (3,338, 376,516)	0.0 (0.0, 0.0)	1.1 (-0.1, 3.9)	1.6 (0.3, 3.5)	1.8 (0.3, 4.0)	1.9 (0.3, 4.3)	1.9 (0.2, 4.4)	2.0 (0.1, 4.6)	2.0 (0.0, 4.6)

Cause of disease or injury	Alcohol-attributable DALYs lost								Population attributable fraction (%)							
	0 to 14	15 to 19	20 to 29	30 to 39	40 to 49	50 to 59	60 to 69	≥70	0 to 14	15 to 19	20 to 29	30 to 39	40 to 49	50 to 59	60 to 69	≥70
Alcohol use disorders	40,056 (40,056, 40,056)	454,251 (454,251, 454,251)	2,851,870 (2,851,870, 2,851,870)	3,343,124 (3,343,124, 3,343,124)	2,978,735 (2,978,735, 2,978,735)	2,331,722 (2,331,722, 2,331,722)	1,197,258 (1,197,258, 1,197,258)	416,703 (416,703, 416,703)	100.0 (100.0, 100.0)	100.0 (100.0, 100.0)	100.0 (100.0, 100.0)	100.0 (100.0, 100.0)	100.0 (100.0, 100.0)	100.0 (100.0, 100.0)	100.0 (100.0, 100.0)	100.0 (100.0, 100.0)
Epilepsy	0 (0, 0)	64,021 (45,405, 99,508)	284,941 (210,744, 357,820)	231,780 (173,396, 286,468)	186,090 (140,654, 228,158)	134,562 (101,400, 167,587)	92,374 (68,963, 115,803)	68,265 (51,320, 86,280)	0.0 (0.0, 0.0)	6.9 (4.9, 10.7)	18.2 (13.5, 22.9)	21.0 (15.7, 26.0)	22.6 (17.0, 27.7)	22.0 (16.6, 27.4)	20.3 (15.2, 25.4)	18.2 (13.7, 23.1)
Cardiovascular diseases	0 (0, 0)	23,811 (16,362, 54,118)	168,700 (61,156, 291,512)	469,829 (217,486, 679,042)	843,426 (345,947, 1,141,173)	1,482,263 (654,532, 2,086,958)	1,714,254 (383,772, 2,861,041)	1,713,998 (-361,931, 3,692,100)	0.0 (0.0, 0.0)	1.4 (0.9, 3.1)	2.7 (1.0, 4.7)	3.8 (1.8, 5.5)	3.5 (1.4, 4.8)	3.4 (1.5, 4.8)	2.9 (0.6, 4.8)	2.2 (-0.5, 4.7)
Hypertensive heart disease	0 (0, 0)	1,995 (1,372, 3,153)	23,629 (17,383, 28,764)	59,804 (45,133, 72,072)	128,188 (97,892, 154,568)	228,874 (175,594, 279,284)	320,736 (238,687, 395,062)	470,823 (340,500, 591,357)	0.0 (0.0, 0.0)	4.5 (3.1, 7.1)	12.7 (9.4, 15.5)	14.8 (11.1, 17.8)	15.5 (11.8, 18.7)	15.0 (11.5, 18.3)	14.1 (10.5, 17.4)	13.2 (9.6, 16.6)
Ischaemic heart disease	0 (0, 0)	351 (-6,428, 10,801)	-18,083 (-116,191, 82,559)	-1,177 (-233,437, 165,310)	3,172 (-437,151, 205,564)	112,549 (-703,410, 520,486)	182,876 (-1,055,934, 1,111,072)	201,094 (-1,752,510, 2,076,849)	0.0 (0.0, 0.0)	0.1 (-1.4, 2.3)	-0.7 (-4.7, 3.4)	0.0 (-3.5, 2.5)	0.0 (-3.2, 1.5)	0.4 (-2.8, 2.1)	0.6 (-3.3, 3.5)	0.5 (-4.4, 5.3)
Ischaemic stroke	0 (0, 0)	-783 (-1,404, 1,185)	-4,414 (-8,912, 8,970)	-6,133 (-16,031, 14,760)	-19,037 (-48,933, 29,352)	-38,302 (-120,172, 86,771)	-116,917 (-348,216, 185,104)	-225,284 (-654,606, 305,166)	0.0 (0.0, 0.0)	-1.1 (-1.9, 1.6)	-1.5 (-3.0, 3.0)	-1.1 (-3.0, 2.8)	-1.3 (-3.4, 2.1)	-1.0 (-3.0, 2.2)	-1.2 (-3.7, 1.9)	-1.5 (-4.3, 2.0)
Haemorrhagic stroke	0 (0, 0)	16,137 (9,972, 29,798)	123,045 (80,067, 168,105)	266,332 (176,879, 357,471)	575,595 (381,062, 772,682)	991,436 (668,911, 1,337,525)	1,217,555 (797,440, 1,694,629)	1,209,274 (774,135, 1,721,223)	0.0 (0.0, 0.0)	4.9 (3.0, 9.1)	10.0 (6.5, 13.6)	11.3 (7.5, 15.2)	11.8 (7.8, 15.9)	11.2 (7.6, 15.2)	10.7 (7.0, 14.9)	10.2 (6.6, 14.6)
Cardiomyopathy, myocarditis, endocarditis	0 (0, 0)	6,111 (4,876, 26,149)	44,522 (42,500, 74,040)	151,003 (149,637, 168,170)	155,507 (154,217, 169,169)	187,707 (186,096, 206,860)	110,004 (108,157, 130,629)	58,091 (56,610, 71,834)	0.0 (0.0, 0.0)	2.6 (2.1, 11.3)	6.6 (6.3, 11.0)	15.4 (15.3, 17.2)	14.3 (14.2, 15.6)	14.8 (14.7, 16.3)	9.7 (9.6, 11.6)	5.0 (4.9, 6.2)
Digestive diseases	0 (0, 0)	148,129 (104,949, 218,577)	1,180,724 (918,820, 1,381,730)	2,801,053 (2,258,287, 3,203,568)	3,892,177 (3,221,427, 4,407,540)	4,046,622 (3,379,296, 4,579,584)	2,959,866 (2,456,591, 3,401,420)	1,425,459 (1,151,655, 1,717,685)	0.0 (0.0, 0.0)	12.3 (8.7, 18.1)	29.4 (22.9, 34.4)	39.5 (31.8, 45.1)	41.2 (34.1, 46.7)	37.7 (31.4, 42.6)	30.8 (25.5, 35.4)	18.3 (14.8, 22.1)
Cirrhosis of the liver	0 (0, 0)	142,238 (99,538, 212,213)	1,082,967 (831,852, 1,279,007)	2,603,315 (2,075,875, 2,993,075)	3,677,996 (3,017,632, 4,181,372)	3,880,028 (3,242,213, 4,416,488)	2,841,519 (2,345,830, 3,279,184)	1,339,787 (1,070,494, 1,626,554)	0.0 (0.0, 0.0)	26.0 (18.2, 38.8)	50.1 (38.4, 59.1)	54.8 (43.7, 63.0)	56.1 (46.0, 63.8)	54.0 (45.1, 61.4)	50.7 (41.9, 58.6)	42.9 (34.3, 52.1)
Pancreatitis	0 (0, 0)	5,891 (3,749, 9,122)	97,758 (63,374, 132,439)	197,737 (137,721, 257,065)	214,182 (143,862, 279,113)	166,594 (116,045, 214,511)	118,347 (79,926, 156,507)	85,672 (55,500, 116,223)	0.0 (0.0, 0.0)	14.7 (9.4, 22.8)	32.7 (21.2, 44.4)	37.4 (26.1, 48.7)	37.9 (25.5, 49.4)	36.8 (25.6, 47.4)	33.6 (22.7, 44.4)	30.4 (19.7, 41.2)
**Injuries**	1,754,663 (1,283,473, 2,893,060)	2,432,457 (1,768,723, 3,606,228)	11,044,784 (8,075,117, 14,630,752)	8,980,202 (6,787,499, 11,640,277)	6,905,474 (5,264,051, 8,890,690)	5,236,292 (4,051,760, 6,761,005)	3,241,205 (2,484,641, 4,274,131)	1,979,746 (1,447,277, 2,735,553)	5.1 (3.7, 8.3)	14.3 (10.4, 21.2)	24.6 (18.0, 32.6)	26.8 (20.3, 34.8)	28.2 (21.5, 36.3)	26.9 (20.8, 34.8)	23.6 (18.1, 31.2)	17.8 (13.0, 24.6)
Unintentional injuries	1,754,663 (1,283,473, 2,893,060)	1,804,721 (1,336,389, 2,715,392)	7,550,874 (5,583,170, 10,244,230)	6,176,749 (4,621,692, 8,206,946)	5,198,433 (3,851,988, 6,906,740)	4,130,178 (3,056,829, 5,488,697)	2,706,607 (1,996,695, 3,656,981)	1,737,970 (1,229,445, 2,464,455)	5.6 (4.1, 9.3)	16.9 (12.5, 25.5)	28.3 (21.0, 38.5)	29.9 (22.3, 39.7)	30.4 (22.5, 40.4)	28.4 (21.0, 37.7)	24.6 (18.1, 33.2)	18.3 (12.9, 25.9)

Cause of disease or injury	Alcohol-attributable DALYs lost								Population attributable fraction (%)							
	0 to 14	15 to 19	20 to 29	30 to 39	40 to 49	50 to 59	60 to 69	≥70	0 to 14	15 to 19	20 to 29	30 to 39	40 to 49	50 to 59	60 to 69	≥70
Road injury	1,754,663 (1,283,473, 2,893,060)	1,221,189 (805,153, 1,960,871)	4,744,414 (3,229,198, 6,962,757)	3,387,326 (2,333,289, 4,920,695)	2,617,685 (1,786,125, 3,817,342)	1,877,837 (1,267,268, 2,759,393)	1,122,728 (761,889, 1,672,847)	574,587 (393,504, 880,888)	22.9 (16.7, 37.7)	20.5 (13.5, 32.9)	30.3 (20.6, 44.5)	31.7 (21.8, 46.0)	32.2 (22.0, 47.0)	30.5 (20.6, 44.8)	28.3 (19.2, 42.1)	24.1 (16.5, 36.9)
Poisonings	0 (0, 0)	22,105 (10,921, 38,510)	137,304 (73,196, 203,909)	108,842 (62,897, 155,829)	91,348 (52,670, 131,523)	64,105 (38,721, 91,342)	41,558 (24,427, 61,084)	19,585 (10,578, 31,120)	0.0 (0.0, 0.0)	11.7 (5.8, 20.4)	25.2 (13.4, 37.4)	27.8 (16.1, 39.8)	27.4 (15.8, 39.5)	26.2 (15.8, 37.4)	21.4 (12.6, 31.5)	14.0 (7.5, 22.2)
Falls	0 (0, 0)	102,153 (53,486, 168,473)	602,477 (336,690, 870,274)	766,495 (450,587, 1,084,226)	868,610 (509,529, 1,228,685)	881,883 (525,024, 1,260,678)	708,198 (416,085, 1,035,901)	644,152 (357,873, 991,882)	0.0 (0.0, 0.0)	13.7 (7.2, 22.7)	26.9 (15.0, 38.8)	28.5 (16.8, 40.4)	29.4 (17.2, 41.5)	27.2 (16.2, 38.8)	22.4 (13.2, 32.8)	16.2 (9.0, 24.9)
Fire, heat and hot substances	0 (0, 0)	26,533 (13,615, 45,910)	156,815 (86,787, 231,259)	181,112 (111,828, 252,863)	153,470 (97,377, 209,761)	134,134 (88,499, 182,178)	85,137 (55,037, 118,390)	48,912 (29,303, 72,768)	0.0 (0.0, 0.0)	11.3 (5.8, 19.6)	23.8 (13.2, 35.1)	27.2 (16.8, 38.0)	29.5 (18.7, 40.4)	28.7 (18.9, 38.9)	24.6 (15.9, 34.2)	17.6 (10.5, 26.1)
Drowning	0 (0, 0)	170,505 (85,511, 290,820)	510,295 (280,103, 749,188)	367,100 (225,369, 513,831)	248,492 (151,569, 347,528)	170,068 (105,586, 239,624)	104,243 (61,299, 151,039)	54,646 (29,613, 84,036)	0.0 (0.0, 0.0)	12.8 (6.4, 21.8)	26.0 (14.3, 38.2)	29.3 (18.0, 41.0)	29.4 (18.0, 41.2)	27.1 (16.9, 38.3)	23.0 (13.5, 33.3)	17.0 (9.2, 26.2)
Exposure to mechanical forces	0 (0, 0)	65,986 (33,688, 111,710)	381,430 (205,964, 559,406)	371,610 (213,334, 529,112)	337,986 (195,887, 482,831)	273,527 (158,808, 391,568)	166,928 (96,418, 242,474)	85,411 (48,935, 128,056)	0.0 (0.0, 0.0)	12.8 (6.5, 21.7)	26.0 (14.0, 38.1)	28.4 (16.3, 40.4)	29.9 (17.3, 42.7)	28.0 (16.3, 40.1)	24.4 (14.1, 35.5)	18.2 (10.4, 27.2)
Other unintentional injuries	0 (0, 0)	196,252 (97,818, 337,806)	1,018,139 (540,680, 1,513,642)	994,263 (575,868, 1,427,091)	880,843 (518,627, 1,250,110)	728,623 (451,217, 1,030,643)	477,815 (288,681, 693,109)	310,676 (172,546, 475,574)	0.0 (0.0, 0.0)	11.7 (5.8, 20.2)	25.0 (13.3, 37.2)	27.3 (15.8, 39.1)	27.8 (16.4, 39.4)	26.1 (16.1, 36.9)	21.8 (13.1, 31.6)	16.1 (8.9, 24.7)
Intentional injuries	0 (0, 0)	627,736 (138,015, 1,236,079)	3,493,910 (998,325, 5,775,035)	2,803,453 (1,056,510, 4,383,571)	1,707,041 (633,062, 2,655,070)	1,106,114 (476,247, 1,696,716)	534,598 (221,829, 845,188)	241,775 (99,941, 408,013)	0.0 (0.0, 0.0)	9.9 (2.2, 19.4)	19.2 (5.5, 31.7)	21.9 (8.3, 34.3)	23.1 (8.6, 35.9)	22.5 (9.7, 34.6)	19.8 (8.2, 31.3)	15.3 (6.3, 25.7)
Self-harm	0 (0, 0)	225,038 (57,843, 425,677)	1,556,703 (545,466, 2,493,931)	1,508,583 (660,801, 2,279,013)	1,040,955 (421,304, 1,577,295)	765,511 (338,286, 1,154,926)	398,950 (163,760, 632,683)	198,631 (80,036, 335,155)	0.0 (0.0, 0.0)	11.7 (3.0, 22.1)	23.9 (8.4, 38.3)	27.6 (12.1, 41.6)	28.2 (11.4, 42.7)	27.1 (12.0, 40.9)	22.7 (9.3, 36.0)	16.7 (6.7, 28.2)
Interpersonal violence	0 (0, 0)	402,697 (81,268, 807,069)	1,937,207 (445,756, 3,281,066)	1,294,870 (385,954, 2,109,171)	666,085 (208,761, 1,076,635)	340,603 (135,493, 540,619)	135,648 (58,809, 215,583)	43,144 (19,827, 72,043)	0.0 (0.0, 0.0)	11.9 (2.4, 23.9)	22.4 (5.1, 37.9)	24.2 (7.2, 39.5)	24.1 (7.5, 38.9)	22.4 (8.9, 35.5)	19.8 (8.6, 31.4)	13.9 (6.4, 23.2)

Table A15. Global alcohol-attributable disability adjusted life years (DALYs) lost by age and cause in 2016 among women and 95% uncertainty intervals

Cause of disease or injury	Alcohol-attributable DALYs lost								Population Attributable Fraction							
	0 to 14	15 to 19	20 to 29	30 to 39	40 to 49	50 to 59	60 to 69	≥70	0 to 14	15 to 19	20 to 29	30 to 39	40 to 49	50 to 59	60 to 69	≥70
**All Causes**	1,230,742 (909,404, 2,005,448)	1,242,424 (1,101,778, 1,728,433)	4,718,333 (4,133,398, 5,957,805)	4,819,773 (4,365,759, 6,096,609)	4,910,569 (4,452,286, 6,233,527)	5,019,878 (4,479,824, 6,565,197)	4,403,723 (3,633,197, 6,287,828)	5,258,816 (3,423,928, 8,270,405)	0.4 (0.3, 0.7)	3.0 (2.6, 4.1)	4.7 (4.1, 5.9)	4.5 (4.1, 5.7)	4.2 (3.8, 5.4)	3.8 (3.4, 4.9)	2.8 (2.3, 4.1)	2.2 (1.4, 3.4)
**Communicable, maternal, perinatal and nutritional conditions**	0 (0, 0)	96,549 (38,742, 308,687)	583,543 (220,582, 1,348,949)	666,304 (330,777, 1,551,150)	516,945 (247,788, 1,218,469)	368,725 (159,699, 943,761)	279,572 (121,265, 717,378)	306,416 (141,952, 728,432)	0.0 (0.0, 0.0)	0.8 (0.3, 2.4)	1.9 (0.7, 4.3)	2.3 (1.1, 5.3)	2.6 (1.3, 6.2)	2.8 (1.2, 7.1)	2.2 (0.9, 5.6)	1.6 (0.7, 3.7)
Tuberculosis	0 (0, 0)	72,287 (11,831, 235,790)	420,170 (60,075, 1,074,207)	412,660 (70,887, 1,060,149)	334,856 (58,269, 871,847)	260,880 (45,872, 744,407)	187,045 (31,356, 561,739)	128,770 (21,232, 421,125)	0.0 (0.0, 0.0)	6.6 (1.1, 21.6)	14.1 (2.0, 36.0)	14.1 (2.4, 36.3)	13.7 (2.4, 35.7)	10.4 (1.8, 29.8)	8.2 (1.4, 24.6)	5.4 (0.9, 17.6)
HIV AIDS	0 (0, 0)	9,198 (5,668, 57,630)	96,850 (56,774, 228,256)	180,208 (105,268, 512,558)	115,258 (68,284, 346,704)	29,399 (18,465, 108,208)	6,648 (4,156, 30,333)	869 (549, 7,846)	0.0 (0.0, 0.0)	0.9 (0.6, 5.7)	2.3 (1.3, 5.3)	2.2 (1.3, 6.4)	2.2 (1.3, 6.7)	1.6 (1.0, 6.0)	1.4 (0.9, 6.3)	0.8 (0.5, 7.0)
Lower respiratory infections	0 (0, 0)	15,064 (5,549, 56,786)	66,522 (27,127, 140,438)	73,436 (40,772, 144,937)	66,831 (35,809, 131,726)	78,445 (39,550, 162,699)	85,879 (37,495, 191,492)	176,777 (66,174, 384,534)	0.0 (0.0, 0.0)	1.8 (0.7, 6.7)	4.3 (1.7, 9.1)	5.4 (3.0, 10.6)	5.1 (2.7, 10.0)	3.9 (1.9, 8.0)	2.5 (1.1, 5.6)	2.0 (0.7, 4.3)
**Noncommunicable diseases**	23,244 (23,244, 23,244)	421,683 (383,165, 496,881)	2,011,835 (1,870,305, 2,243,437)	2,430,994 (2,235,041, 2,813,540)	2,814,683 (2,470,526, 3,501,907)	3,357,989 (2,831,805, 4,430,843)	3,115,843 (2,317,626, 4,626,114)	4,055,128 (2,195,789, 6,810,179)	0.0 (0.0, 0.0)	2.0 (1.8, 2.3)	3.7 (3.5, 4.2)	3.7 (3.4, 4.3)	3.2 (2.8, 4.0)	3.0 (2.6, 4.0)	2.3 (1.7, 3.5)	1.9 (1.0, 3.2)
Malignant neoplasms	0 (0, 0)	0 (0, 0)	28,253 (22,725, 41,748)	273,845 (225,903, 353,396)	566,994 (478,849, 714,820)	819,374 (707,628, 1,014,953)	716,294 (611,736, 894,656)	651,780 (524,941, 845,888)	0.0 (0.0, 0.0)	0.0 (0.0, 0.0)	0.8 (0.6, 1.1)	3.2 (2.6, 4.1)	3.4 (2.9, 4.3)	3.4 (3.0, 4.3)	3.0 (2.5, 3.7)	2.5 (2.0, 3.3)
Lip and oral cavity cancer	0 (0, 0)	0 (0, 0)	2,657 (1,762, 4,033)	20,791 (14,967, 27,640)	49,885 (35,185, 67,169)	76,440 (56,144, 102,184)	63,831 (46,865, 86,779)	46,583 (34,443, 64,535)	0.0 (0.0, 0.0)	0.0 (0.0, 0.0)	5.5 (3.6, 8.3)	16.6 (12.0, 22.1)	17.3 (12.2, 23.2)	18.2 (13.4, 24.4)	16.1 (11.8, 21.9)	15.1 (11.1, 20.9)
Other pharynx cancers	0 (0, 0)	0 (0, 0)	1,102 (712, 1,792)	8,774 (5,900, 11,999)	20,011 (13,822, 27,624)	31,433 (24,095, 41,591)	26,864 (20,576, 36,428)	16,096 (11,965, 22,695)	0.0 (0.0, 0.0)	0.0 (0.0, 0.0)	4.9 (3.2, 8.0)	15.6 (10.5, 21.3)	17.1 (11.8, 23.5)	19.2 (14.7, 25.4)	17.3 (13.2, 23.4)	14.6 (10.9, 20.6)
Oesophagus cancer	0 (0, 0)	0 (0, 0)	1,047 (619, 1,874)	10,067 (7,168, 14,374)	27,559 (20,225, 39,347)	57,748 (45,057, 77,431)	73,350 (53,995, 101,670)	68,826 (47,114, 103,561)	0.0 (0.0, 0.0)	0.0 (0.0, 0.0)	2.2 (1.3, 3.9)	8.1 (5.7, 11.5)	8.4 (6.2, 12.0)	9.6 (7.5, 12.9)	8.9 (6.5, 12.3)	7.4 (5.0, 11.1)
Colon and rectum cancers	0 (0, 0)	0 (0, 0)	2,948 (1,360, 5,579)	23,514 (13,946, 36,229)	48,145 (29,508, 73,704)	90,512 (55,604, 136,624)	101,344 (57,914, 158,160)	127,703 (63,156, 210,613)	0.0 (0.0, 0.0)	0.0 (0.0, 0.0)	1.5 (0.7, 2.9)	4.7 (2.8, 7.3)	5.0 (3.1, 7.7)	5.6 (3.5, 8.5)	5.1 (2.9, 7.9)	4.2 (2.1, 7.0)
Liver cancer	0 (0, 0)	0 (0, 0)	8,445 (4,167, 15,168)	35,764 (18,973, 60,520)	89,271 (47,353, 150,100)	147,072 (77,395, 245,957)	168,805 (86,489, 285,441)	193,677 (93,967, 331,239)	0.0 (0.0, 0.0)	0.0 (0.0, 0.0)	6.0 (3.0, 10.9)	10.6 (5.6, 17.9)	10.1 (5.4, 17.0)	10.4 (5.5, 17.4)	10.6 (5.4, 18.0)	10.9 (5.3, 18.6)
Breast cancer	0 (0, 0)	0 (0, 0)	11,852 (9,194, 18,615)	173,515 (135,171, 231,633)	328,710 (263,191, 422,640)	407,591 (332,058, 516,948)	272,552 (223,921, 344,259)	192,258 (157,972, 242,040)	0.0 (0.0, 0.0)	0.0 (0.0, 0.0)	2.4 (1.9, 3.8)	7.1 (5.5, 9.5)	7.5 (6.0, 9.6)	8.2 (6.7, 10.4)	7.5 (6.1, 9.5)	6.7 (5.5, 8.5)
Larynx cancer	0 (0, 0)	0 (0, 0)	204 (99, 398)	1,422 (877, 2,161)	3,414 (2,260, 5,123)	8,577 (5,962, 12,525)	9,548 (6,468, 14,425)	6,636 (4,010, 10,693)	0.0 (0.0, 0.0)	0.0 (0.0, 0.0)	3.0 (1.4, 5.8)	9.6 (5.9, 14.6)	10.2 (6.8, 15.4)	11.4 (7.9, 16.6)	10.6 (7.2, 16.1)	8.6 (5.2, 13.9)
Diabetes mellitus	0 (0, 0)	-9,863 (-14,082, -2,462)	-94,325 (-129,603, -51,275)	-174,734 (-238,982, -95,538)	-362,012 (-496,016, -199,578)	-516,878 (-719,077, -277,026)	-567,214 (-791,554, -290,787)	-562,454 (-801,061, -285,347)	0.0 (0.0, 0.0)	-3.7 (-5.2, -0.9)	-8.6 (-11.9, -4.7)	-8.8 (-12.0, -4.8)	-9.1 (-12.5, -5.0)	-7.7 (-10.7, -4.1)	-6.6 (-9.2, -3.4)	-5.6 (-8.0, -2.8)
Alcohol use disorders	23,244 (23,244, 23,244)	225,343 (225,343, 225,343)	1,225,343 (1,225,343, 1,225,343)	1,174,515 (1,174,515, 1,174,515)	913,783 (913,783, 913,783)	713,738 (713,738, 713,738)	376,306 (376,306, 376,306)	188,888 (188,888, 188,888)	100.0 (100.0, 100.0)	100.0 (100.0, 100.0)	100.0 (100.0, 100.0)	100.0 (100.0, 100.0)	100.0 (100.0, 100.0)	100.0 (100.0, 100.0)	100.0 (100.0, 100.0)	100.0 (100.0, 100.0)

Cause of disease or injury	Alcohol-attributable DALYs lost								Population Attributable Fraction							
	0 to 14	15 to 19	20 to 29	30 to 39	40 to 49	50 to 59	60 to 69	≥70	0 to 14	15 to 19	20 to 29	30 to 39	40 to 49	50 to 59	60 to 69	≥70
Epilepsy	0 (0, 0)	28,407 (21,823, 49,309)	107,652 (81,465, 150,528)	77,233 (59,219, 108,794)	59,300 (45,548, 81,344)	42,733 (32,990, 58,927)	28,673 (22,126, 39,677)	27,224 (21,216, 37,849)	0.0 (0.0, 0.0)	4.4 (3.4, 7.6)	9.2 (7.0, 12.9)	9.3 (7.1, 13.1)	9.7 (7.4, 13.3)	8.2 (6.3, 11.2)	6.9 (5.3, 9.5)	5.9 (4.6, 8.2)
Cardiovascular diseases	0 (0, 0)	27,874 (17,231, 61,666)	123,794 (49,616, 270,360)	280,681 (138,704, 545,143)	576,090 (282,295, 1,104,507)	964,637 (479,193, 1,858,768)	1,319,286 (536,235, 2,690,061)	2,905,542 (985,114, 5,547,425)	0.0 (0.0, 0.0)	1.8 (1.1, 4.0)	3.0 (1.2, 6.5)	3.9 (1.9, 7.7)	4.2 (2.0, 8.0)	3.8 (1.9, 7.3)	3.1 (1.2, 6.2)	3.3 (1.1, 6.4)
Hypertensive heart disease	0 (0, 0)	993 (467, 2,797)	6,724 (3,066, 12,899)	15,455 (7,368, 31,328)	33,499 (15,890, 68,873)	52,007 (24,992, 112,103)	63,552 (29,772, 149,338)	113,844 (52,866, 267,765)	0.0 (0.0, 0.0)	1.9 (0.9, 5.4)	4.5 (2.1, 8.7)	4.5 (2.1, 9.1)	4.4 (2.1, 9.1)	3.5 (1.7, 7.6)	2.7 (1.3, 6.3)	2.2 (1.0, 5.3)
Ischaemic heart disease	0 (0, 0)	6,408 (-741, 20,222)	29,053 (-38,919, 129,246)	72,178 (-60,398, 262,496)	143,453 (-121,309, 523,040)	362,412 (-118,918, 1,003,321)	735,312 (-97,670, 1,652,281)	2,318,932 (309,866, 4,046,604)	0.0 (0.0, 0.0)	1.9 (-0.2, 6.1)	2.3 (-3.1, 10.3)	2.7 (-2.3, 9.8)	2.5 (-2.1, 9.1)	3.2 (-1.1, 8.9)	3.7 (-0.5, 8.3)	5.4 (0.7, 9.5)
Ischaemic stroke	0 (0, 0)	-1,155 (-1,560, 12,256)	-7,525 (-12,187, 52,332)	-17,684 (-27,043, 36,293)	-64,013 (-98,918, 262)	-156,497 (-254,129, -14,540)	-282,696 (-527,192, 145,344)	-489,249 (-1,176,048, 738,876)	0.0 (0.0, 0.0)	-1.2 (-1.6, 12.5)	-2.6 (-4.2, 18.0)	-3.6 (-5.5, 7.4)	-5.2 (-8.1, 0.0)	-5.3 (-8.6, -0.5)	-3.7 (-6.8, 1.9)	-2.7 (-6.6, 4.2)
Haemorrhagic stroke	0 (0, 0)	20,863 (11,739, 38,770)	88,790 (51,461, 149,163)	188,194 (112,343, 314,493)	439,411 (260,972, 729,115)	673,238 (404,843, 1,120,459)	778,695 (462,913, 1,308,542)	927,342 (570,424, 1,555,497)	0.0 (0.0, 0.0)	6.7 (3.8, 12.4)	11.3 (6.5, 18.9)	11.4 (6.8, 19.0)	12.0 (7.1, 19.9)	10.3 (6.2, 17.1)	8.8 (5.2, 14.7)	7.8 (4.8, 13.1)
Cardiomyopathy, myocarditis, endocarditis	0 (0, 0)	765 (763, 831)	6,752 (6,749, 6,885)	22,537 (22,534, 22,654)	23,740 (23,737, 23,839)	33,477 (33,473, 33,568)	24,423 (24,420, 24,522)	34,673 (34,665, 34,819)	0.0 (0.0, 0.0)	0.5 (0.5, 0.6)	1.6 (1.6, 1.6)	4.7 (4.7, 4.7)	4.7 (4.7, 4.7)	5.7 (5.7, 5.7)	3.7 (3.7, 3.7)	2.7 (2.7, 2.7)
Digestive diseases	0 (0, 0)	149,923 (115,339, 194,513)	621,118 (507,195, 732,264)	799,454 (689,053, 915,748)	1,060,527 (926,907, 1,204,141)	1,334,385 (1,157,363, 1,539,172)	1,242,499 (1,055,380, 1,475,143)	844,149 (703,977, 1,058,618)	0.0 (0.0, 0.0)	12.5 (9.6, 16.2)	22.4 (18.3, 26.5)	24.5 (21.1, 28.1)	24.9 (21.8, 28.3)	24.4 (21.1, 28.1)	20.0 (17.0, 23.8)	10.9 (9.1, 13.7)
Cirrhosis of the liver	0 (0, 0)	147,873 (113,134, 191,822)	608,062 (496,161, 708,573)	777,490 (667,013, 883,570)	1,035,445 (898,718, 1,165,823)	1,305,870 (1,132,925, 1,498,701)	1,216,843 (1,031,697, 1,438,808)	817,063 (675,150, 1,023,503)	0.0 (0.0, 0.0)	32.2 (24.7, 41.8)	48.5 (39.6, 56.5)	51.1 (43.9, 58.1)	52.9 (45.9, 59.5)	48.6 (42.2, 55.8)	43.3 (36.7, 51.2)	34.9 (28.8, 43.7)
Pancreatitis	0 (0, 0)	2,050 (446, 5,886)	13,056 (3,191, 34,738)	21,963 (9,522, 49,253)	25,083 (7,686, 63,286)	28,515 (10,714, 68,357)	25,656 (10,194, 59,151)	27,086 (9,416, 62,298)	0.0 (0.0, 0.0)	6.5 (1.4, 18.5)	11.9 (2.9, 31.7)	14.1 (6.1, 31.7)	12.9 (3.9, 32.4)	11.9 (4.5, 28.5)	9.9 (3.9, 22.7)	8.5 (3.0, 19.5)
**Injuries**	1,207,498 (886,160, 1,982,204)	724,191 (580,786, 1,042,451)	2,122,955 (1,638,066, 2,915,748)	1,722,475 (1,395,229, 2,300,541)	1,578,941 (1,273,817, 2,124,054)	1,293,164 (1,085,726, 1,711,665)	1,008,308 (851,065, 1,358,955)	897,272 (744,657, 1,255,114)	4.8 (3.5, 7.9)	9.3 (7.4, 13.3)	13.6 (10.5, 18.7)	14.8 (12.0, 19.7)	16.2 (13.1, 21.8)	14.1 (11.8, 18.6)	11.7 (9.8, 15.7)	8.4 (7.0, 11.8)
Unintentional injuries	1,207,498 (886,160, 1,982,204)	556,586 (457,366, 808,258)	1,493,823 (1,209,871, 2,041,263)	1,268,968 (1,032,877, 1,725,661)	1,259,469 (1,021,307, 1,727,363)	1,090,815 (917,161, 1,477,429)	889,643 (742,913, 1,220,807)	830,433 (678,878, 1,171,236)	5.5 (4.0, 8.9)	12.5 (10.3, 18.2)	17.1 (13.9, 23.4)	17.1 (13.9, 23.2)	18.0 (14.6, 24.7)	15.1 (12.7, 20.5)	12.3 (10.2, 16.8)	8.6 (7.0, 12.1)
Road injury	1,207,498 (886,160, 1,982,204)	365,021 (286,712, 538,191)	834,659 (658,767, 1,183,981)	643,015 (518,656, 907,005)	638,969 (511,827, 911,392)	531,700 (427,900, 770,260)	414,731 (334,358, 605,893)	305,172 (248,045, 447,541)	23.4 (17.2, 38.5)	19.7 (15.5, 29.0)	23.6 (18.7, 33.5)	23.5 (18.9, 33.1)	24.0 (19.2, 34.2)	21.7 (17.4, 31.4)	20.9 (16.9, 30.6)	19.8 (16.1, 29.0)

Cause of disease or injury	Alcohol-attributable DALYs lost								Population Attributable Fraction							
	0 to 14	15 to 19	20 to 29	30 to 39	40 to 49	50 to 59	60 to 69	≥70	0 to 14	15 to 19	20 to 29	30 to 39	40 to 49	50 to 59	60 to 69	≥70
Poisonings	0 (0, 0)	11,290 (5,850, 21,700)	44,659 (23,838, 73,536)	32,653 (18,564, 52,398)	30,337 (16,750, 49,096)	18,003 (10,329, 29,025)	13,093 (7,602, 21,689)	6,442 (4,274, 10,669)	0.0 (0.0, 0.0)	8.2 (4.2, 15.7)	13.4 (7.1, 22.0)	14.5 (8.2, 23.2)	13.6 (7.5, 22.0)	10.2 (5.8, 16.4)	7.2 (4.2, 12.0)	5.2 (3.4, 8.6)
Falls	0 (0, 0)	41,681 (22,801, 69,562)	163,880 (99,049, 249,125)	185,418 (118,080, 273,598)	222,092 (139,426, 330,975)	250,221 (165,334, 369,198)	247,591 (167,695, 369,160)	335,011 (228,896, 514,531)	0.0 (0.0, 0.0)	9.0 (4.9, 15.0)	15.0 (9.0, 22.8)	15.7 (10.0, 23.1)	16.3 (10.2, 24.3)	12.9 (8.5, 19.0)	9.3 (6.3, 13.9)	6.5 (4.5, 10.0)
Fire, heat and hot substances	0 (0, 0)	28,081 (13,387, 50,709)	101,782 (54,802, 168,192)	85,552 (51,075, 135,779)	61,317 (38,225, 95,153)	46,811 (32,109, 69,454)	32,173 (22,841, 47,591)	24,924 (18,915, 36,860)	0.0 (0.0, 0.0)	5.7 (2.7, 10.4)	10.4 (5.6, 17.2)	10.9 (6.5, 17.3)	12.4 (7.7, 19.2)	10.7 (7.3, 15.9)	9.0 (6.4, 13.3)	7.0 (5.3, 10.4)
Drowning	0 (0, 0)	32,596 (16,991, 59,243)	69,853 (38,535, 113,327)	53,255 (31,923, 82,978)	42,568 (24,720, 67,146)	28,198 (16,401, 45,506)	21,416 (12,188, 36,229)	15,966 (9,531, 28,329)	0.0 (0.0, 0.0)	7.8 (4.1, 14.2)	13.4 (7.4, 21.7)	14.4 (8.6, 22.4)	15.4 (8.9, 24.3)	10.9 (6.3, 17.6)	8.3 (4.7, 14.0)	5.5 (3.3, 9.8)
Exposure to mechanical forces	0 (0, 0)	14,841 (7,975, 26,211)	54,256 (31,100, 84,856)	58,407 (35,067, 89,959)	62,605 (37,197, 97,146)	48,624 (31,163, 73,366)	34,699 (23,095, 52,204)	24,823 (17,780, 37,056)	0.0 (0.0, 0.0)	8.0 (4.3, 14.1)	13.8 (7.9, 21.6)	14.6 (8.7, 22.4)	15.3 (9.1, 23.8)	12.5 (8.0, 18.9)	10.3 (6.8, 15.5)	7.6 (5.5, 11.4)
Other unintentional injuries	0 (0, 0)	63,076 (33,588, 113,954)	224,735 (127,188, 363,289)	210,668 (128,940, 329,897)	201,582 (125,184, 311,483)	167,258 (110,757, 250,014)	125,940 (85,379, 189,834)	118,094 (79,050, 185,397)	0.0 (0.0, 0.0)	7.2 (3.8, 13.0)	12.1 (6.9, 19.6)	12.4 (7.6, 19.4)	13.2 (8.2, 20.4)	10.9 (7.2, 16.3)	8.6 (5.8, 13.0)	6.3 (4.2, 9.9)
Intentional injuries	0 (0, 0)	167,605 (55,489, 336,836)	629,131 (221,600, 1,151,867)	453,508 (209,833, 771,264)	319,472 (142,223, 548,199)	202,349 (105,114, 334,286)	118,665 (64,342, 196,953)	66,839 (43,569, 107,292)	0.0 (0.0, 0.0)	5.0 (1.6, 10.0)	9.1 (3.2, 16.7)	10.7 (4.9, 18.1)	11.6 (5.2, 20.0)	10.2 (5.3, 16.8)	8.5 (4.6, 14.2)	6.8 (4.4, 10.8)
Self-harm	0 (0, 0)	111,711 (34,599, 219,851)	424,690 (143,826, 785,551)	290,992 (123,703, 501,630)	217,706 (88,023, 382,233)	143,947 (65,990, 248,379)	87,105 (41,828, 151,515)	48,417 (28,922, 82,213)	0.0 (0.0, 0.0)	5.5 (1.7, 10.9)	9.9 (3.4, 18.3)	12.4 (5.3, 21.3)	13.4 (5.4, 23.6)	10.7 (4.9, 18.5)	8.5 (4.1, 14.8)	6.5 (3.9, 11.0)
Interpersonal violence	0 (0, 0)	55,894 (17,605, 114,104)	204,442 (75,842, 373,742)	162,516 (83,460, 272,858)	101,766 (54,117, 165,957)	58,402 (39,255, 84,424)	31,560 (22,630, 44,632)	18,422 (14,580, 25,259)	0.0 (0.0, 0.0)	6.9 (2.2, 14.1)	11.4 (4.2, 20.8)	12.9 (6.6, 21.7)	13.5 (7.2, 22.0)	12.8 (8.6, 18.5)	11.4 (8.2, 16.1)	9.2 (7.2, 12.6)

Table A16. Global alcohol-attributable disability adjusted life years (DALYs) lost by age and cause in 2016 and 95% uncertainty intervals

Cause of disease or injury	Alcohol-attributable DALYs lost								Population Attributable Fraction							
	0 to 14	15 to 19	20 to 29	30 to 39	40 to 49	50 to 59	60 to 69	≥70	0 to 14	15 to 19	20 to 29	30 to 39	40 to 49	50 to 59	60 to 69	≥70
All Causes	3,025,460 (2,232,750, 4,937,606)	4,515,366 (3,831,041, 6,081,656)	21,898,349 (18,596,964, 26,378,508)	23,486,159 (20,648,423, 27,358,081)	23,496,263 (20,799,000, 26,893,983)	22,970,628 (20,432,013, 26,195,643)	17,832,392 (15,512,620, 21,086,175)	14,193,760 (11,231,333, 18,613,217)	0.5 (0.3, 0.8)	5.0 (4.2, 6.7)	9.8 (8.3, 11.8)	9.9 (8.7, 11.5)	9.0 (7.9, 10.3)	7.4 (6.6, 8.4)	5.1 (4.4, 6.0)	3.0 (2.4, 3.9)
Communicable, maternal, perinatal and nutritional conditions	0 (0, 0)	243,822 (134,234, 617,727)	2,156,120 (1,041,764, 3,562,030)	3,028,366 (1,642,582, 4,727,415)	2,931,078 (1,500,212, 4,598,726)	2,388,521 (1,128,338, 3,958,566)	1,671,895 (819,336, 2,864,300)	1,488,560 (807,856, 2,541,784)	0.0 (0.0, 0.0)	1.1 (0.6, 2.7)	4.0 (2.0, 6.7)	5.4 (2.9, 8.4)	6.7 (3.4, 10.5)	7.4 (3.5, 12.3)	5.9 (2.9, 10.1)	3.9 (2.1, 6.7)
Tuberculosis	0 (0, 0)	184,793 (70,457, 454,999)	1,685,369 (578,855, 3,012,240)	2,103,675 (719,823, 3,679,726)	2,107,993 (688,521, 3,709,450)	1,825,875 (580,837, 3,337,356)	1,220,151 (379,153, 2,362,273)	786,021 (242,059, 1,612,259)	0.0 (0.0, 0.0)	8.5 (3.2, 20.9)	22.0 (7.5, 39.3)	24.6 (8.4, 43.1)	25.4 (8.3, 44.8)	22.1 (7.0, 40.4)	18.0 (5.6, 34.8)	13.0 (4.0, 26.6)
HIV AIDS	0 (0, 0)	20,889 (14,799, 111,080)	249,365 (172,862, 405,506)	640,110 (436,392, 1,037,490)	521,137 (357,338, 824,751)	180,715 (123,714, 291,328)	42,206 (28,229, 70,761)	6,988 (4,547, 14,567)	0.0 (0.0, 0.0)	1.0 (0.7, 5.1)	3.0 (2.1, 4.9)	3.5 (2.4, 5.6)	3.8 (2.6, 6.0)	3.5 (2.4, 5.6)	3.1 (2.1, 5.2)	2.2 (1.4, 4.6)
Lower respiratory infections	0 (0, 0)	38,139 (19,510, 116,683)	221,386 (112,364, 377,854)	284,581 (172,909, 439,668)	301,948 (175,622, 477,040)	381,932 (217,398, 606,834)	409,538 (203,206, 699,748)	695,551 (317,409, 1,241,566)	0.0 (0.0, 0.0)	2.2 (1.1, 6.6)	6.2 (3.1, 10.6)	8.4 (5.1, 13.0)	8.5 (4.9, 13.4)	7.4 (4.2, 11.7)	5.3 (2.6, 9.0)	4.0 (1.8, 7.1)
Noncommunicable diseases	63,300 (63,300, 63,300)	1,114,896 (1,048,381, 1,274,212)	6,574,491 (6,191,098, 6,975,203)	9,755,116 (8,997,457, 10,500,276)	12,080,770 (10,930,352, 13,201,846)	14,052,650 (12,590,947, 15,704,772)	11,910,985 (10,115,601, 14,231,329)	9,828,183 (6,971,988, 13,575,565)	0.0 (0.0, 0.0)	2.6 (2.4, 3.0)	6.0 (5.6, 6.3)	7.2 (6.6, 7.7)	6.6 (5.9, 7.2)	5.6 (5.0, 6.3)	4.0 (3.4, 4.8)	2.4 (1.7, 3.3)
Malignant neoplasms	0 (0, 0)	0 (0, 0)	84,238 (68,797, 116,992)	709,133 (587,159, 860,332)	1,842,143 (1,543,022, 2,202,273)	3,375,190 (2,890,037, 3,927,485)	3,387,315 (2,922,431, 3,921,667)	2,639,463 (2,273,817, 3,128,848)	0.0 (0.0, 0.0)	0.0 (0.0, 0.0)	1.1 (0.9, 1.5)	4.6 (3.8, 5.6)	5.7 (4.8, 6.8)	6.3 (5.4, 7.3)	5.5 (4.8, 6.4)	4.2 (3.6, 5.0)
Lip and oral cavity cancer	0 (0, 0)	0 (0, 0)	14,731 (9,612, 22,142)	145,434 (104,043, 177,783)	318,218 (246,648, 374,885)	521,535 (428,351, 596,614)	430,734 (360,100, 495,387)	227,635 (193,493, 264,436)	0.0 (0.0, 0.0)	0.0 (0.0, 0.0)	7.7 (5.0, 11.5)	30.0 (21.5, 36.7)	32.2 (24.9, 37.9)	35.4 (29.0, 40.5)	33.8 (28.2, 38.8)	28.7 (24.4, 33.3)
Other pharynx cancers	0 (0, 0)	0 (0, 0)	4,118 (2,777, 6,088)	55,100 (40,335, 67,090)	200,235 (156,589, 234,826)	416,487 (347,013, 472,210)	353,502 (296,090, 402,596)	166,405 (138,542, 193,898)	0.0 (0.0, 0.0)	0.0 (0.0, 0.0)	7.1 (4.8, 10.5)	28.4 (20.8, 34.6)	34.6 (27.0, 40.5)	39.9 (33.2, 45.2)	38.2 (32.0, 43.5)	30.9 (25.7, 35.9)
Oesophagus cancer	0 (0, 0)	0 (0, 0)	3,446 (2,537, 4,951)	40,337 (33,239, 48,358)	210,090 (178,270, 243,774)	615,910 (516,517, 707,668)	761,287 (623,282, 891,483)	539,339 (431,193, 654,223)	0.0 (0.0, 0.0)	0.0 (0.0, 0.0)	3.4 (2.5, 4.8)	12.8 (10.5, 15.3)	18.5 (15.7, 21.5)	22.4 (18.7, 25.7)	21.7 (17.7, 25.4)	17.8 (14.2, 21.6)
Colon and rectum cancers	0 (0, 0)	0 (0, 0)	16,242 (13,626, 20,663)	100,802 (82,279, 124,392)	229,984 (187,635, 280,598)	478,653 (392,836, 576,005)	638,901 (522,264, 765,555)	758,401 (622,720, 921,918)	0.0 (0.0, 0.0)	0.0 (0.0, 0.0)	3.8 (3.2, 4.8)	9.6 (7.8, 11.8)	10.8 (8.8, 13.2)	12.4 (10.2, 14.9)	12.8 (10.5, 15.3)	11.7 (9.6, 14.2)
Liver cancer	0 (0, 0)	0 (0, 0)	32,957 (20,002, 56,430)	181,890 (103,328, 271,083)	487,882 (271,740, 740,400)	754,306 (429,460, 1,128,539)	733,244 (435,591, 1,091,627)	635,052 (383,092, 947,245)	0.0 (0.0, 0.0)	0.0 (0.0, 0.0)	5.5 (3.3, 9.3)	11.5 (6.5, 17.1)	11.8 (6.6, 17.9)	12.4 (7.1, 18.6)	12.6 (7.5, 18.7)	12.4 (7.5, 18.5)
Breast cancer	0 (0, 0)	0 (0, 0)	11,852 (9,195, 18,629)	173,515 (135,171, 231,633)	328,710 (263,191, 422,640)	407,591 (332,058, 516,951)	272,552 (223,921, 344,259)	192,258 (157,973, 242,040)	0.0 (0.0, 0.0)	0.0 (0.0, 0.0)	2.4 (1.9, 3.8)	7.1 (5.5, 9.5)	7.5 (6.0, 9.6)	8.2 (6.7, 10.4)	7.4 (6.1, 9.4)	6.7 (5.5, 8.4)
Larynx cancer	0 (0, 0)	0 (0, 0)	893 (631, 1,353)	12,055 (8,977, 15,405)	67,024 (50,531, 84,301)	180,708 (140,102, 222,432)	197,093 (151,884, 244,253)	120,373 (90,387, 151,852)	0.0 (0.0, 0.0)	0.0 (0.0, 0.0)	4.1 (2.9, 6.2)	17.9 (13.3, 22.9)	21.7 (16.4, 27.3)	24.6 (19.0, 30.2)	24.1 (18.5, 29.8)	20.8 (15.6, 26.2)
Diabetes mellitus	0 (0, 0)	-6,862 (-11,927, 7,081)	-73,888 (-110,901, -21,443)	-131,684 (-202,287, -28,926)	-271,503 (-421,636, -60,527)	-373,203 (-600,449, -59,935)	-406,847 (-669,390, -45,262)	-401,508 (-687,120, -28,381)	0.0 (0.0, 0.0)	-1.3 (-2.2, 1.3)	-3.1 (-4.7, -0.9)	-3.0 (-4.6, -0.7)	-3.1 (-4.8, -0.7)	-2.6 (-4.2, -0.4)	-2.4 (-4.0, -0.3)	-2.2 (-3.8, -0.2)

Cause of disease or injury	Alcohol-attributable DALYs lost								Population Attributable Fraction							
	0 to 14	15 to 19	20 to 29	30 to 39	40 to 49	50 to 59	60 to 69	≥70	0 to 14	15 to 19	20 to 29	30 to 39	40 to 49	50 to 59	60 to 69	≥70
Alcohol use disorders	63,300 (63,300, 63,300)	679,595 (679,595, 679,595)	4,077,212 (4,077,212, 4,077,212)	4,517,639 (4,517,639, 4,517,639)	3,892,519 (3,892,519, 3,892,519)	3,045,460 (3,045,460, 3,045,460)	1,573,564 (1,573,564, 1,573,564)	605,591 (605,591, 605,591)	100.0 (100.0, 100.0)	100.0 (100.0, 100.0)	100.0 (100.0, 100.0)	100.0 (100.0, 100.0)	100.0 (100.0, 100.0)	100.0 (100.0, 100.0)	100.0 (100.0, 100.0)	100.0 (100.0, 100.0)
Epilepsy	0 (0, 0)	92,428 (73,752, 142,400)	392,593 (311,788, 484,542)	309,013 (246,966, 378,590)	245,389 (196,006, 297,993)	177,295 (142,127, 215,564)	121,047 (97,000, 148,129)	95,490 (77,385, 117,201)	0.0 (0.0, 0.0)	5.8 (4.7, 9.0)	14.4 (11.4, 17.7)	16.0 (12.8, 19.6)	17.1 (13.6, 20.7)	15.6 (12.5, 19.0)	13.9 (11.1, 17.0)	11.4 (9.3, 14.0)
Cardiovascular diseases	0 (0, 0)	51,684 (39,284, 103,866)	292,494 (167,683, 489,227)	750,511 (470,614, 1,091,239)	1,419,517 (843,801, 1,999,703)	2,446,900 (1,464,270, 3,481,331)	3,033,540 (1,514,236, 4,817,186)	4,619,540 (1,928,010, 8,055,246)	0.0 (0.0, 0.0)	1.6 (1.2, 3.2)	2.8 (1.6, 4.7)	3.9 (2.4, 5.6)	3.8 (2.2, 5.3)	3.6 (2.1, 5.1)	2.9 (1.5, 4.7)	2.8 (1.2, 4.9)
Hypertensive heart disease	0 (0, 0)	2,989 (2,162, 5,578)	30,353 (23,211, 39,205)	75,260 (58,949, 96,463)	161,688 (127,473, 207,488)	280,881 (221,771, 360,751)	384,288 (297,502, 500,310)	584,667 (446,294, 786,036)	0.0 (0.0, 0.0)	3.1 (2.2, 5.8)	9.1 (7.0, 11.8)	10.0 (7.9, 12.9)	10.2 (8.1, 13.1)	9.3 (7.4, 12.0)	8.3 (6.4, 10.8)	6.8 (5.2, 9.1)
Ischaemic heart disease	0 (0, 0)	6,760 (-3,098, 25,687)	10,970 (-100,787, 153,266)	71,001 (-189,501, 318,356)	146,625 (-374,166, 529,021)	474,961 (-441,163, 1,191,879)	918,188 (-500,443, 2,211,934)	2,520,027 (-211,940, 5,125,196)	0.0 (0.0, 0.0)	0.8 (-0.4, 3.2)	0.3 (-2.7, 4.1)	0.8 (-2.0, 3.4)	0.7 (-1.9, 2.7)	1.3 (-1.2, 3.3)	1.8 (-1.0, 4.3)	3.1 (-0.3, 6.2)
Ischaemic stroke	0 (0, 0)	-1,939 (-2,430, 12,189)	-11,939 (-17,399, 50,813)	-23,817 (-35,620, 38,849)	-83,050 (-126,943, 12,334)	-194,799 (-327,815, 26,588)	-399,614 (-722,893, 190,996)	-714,533 (-1,505,875, 780,763)	0.0 (0.0, 0.0)	-1.1 (-1.4, 7.2)	-2.0 (-3.0, 8.7)	-2.3 (-3.5, 3.8)	-3.1 (-4.8, 0.5)	-2.8 (-4.7, 0.4)	-2.3 (-4.2, 1.1)	-2.2 (-4.5, 2.4)
Haemorrhagic stroke	0 (0, 0)	37,000 (26,255, 61,833)	211,836 (155,815, 286,930)	454,527 (342,254, 613,104)	1,015,006 (755,801, 1,367,482)	1,664,673 (1,238,764, 2,228,628)	1,996,250 (1,448,641, 2,767,658)	2,136,615 (1,560,950, 2,992,448)	0.0 (0.0, 0.0)	5.8 (4.1, 9.6)	10.5 (7.7, 14.2)	11.3 (8.5, 15.3)	11.9 (8.8, 16.0)	10.8 (8.1, 14.5)	9.9 (7.1, 13.7)	9.0 (6.6, 12.6)
Cardiomyopathy, myocarditis, endocarditis	0 (0, 0)	6,875 (5,641, 26,918)	51,274 (49,256, 80,849)	173,540 (172,182, 190,710)	179,248 (177,960, 192,923)	221,184 (219,599, 240,363)	134,428 (132,595, 155,116)	92,764 (91,303, 106,549)	0.0 (0.0, 0.0)	1.8 (1.5, 7.2)	4.7 (4.5, 7.4)	11.9 (11.8, 13.1)	11.3 (11.2, 12.1)	11.9 (11.9, 13.0)	7.5 (7.4, 8.7)	3.8 (3.7, 4.3)
Digestive diseases	0 (0, 0)	298,052 (240,953, 386,506)	1,801,842 (1,488,195, 2,061,371)	3,600,506 (3,027,479, 4,041,152)	4,952,705 (4,251,217, 5,532,749)	5,381,007 (4,702,305, 5,983,523)	4,202,365 (3,654,117, 4,751,294)	2,269,607 (1,954,330, 2,660,148)	0.0 (0.0, 0.0)	12.4 (10.0, 16.0)	26.6 (22.0, 30.4)	34.7 (29.2, 39.0)	36.1 (31.0, 40.4)	33.2 (29.0, 36.9)	26.6 (23.1, 30.0)	14.6 (12.6, 17.1)
Cirrhosis of the liver	0 (0, 0)	290,111 (232,663, 378,993)	1,691,028 (1,386,767, 1,937,664)	3,380,806 (2,812,724, 3,805,999)	4,713,440 (4,027,135, 5,265,811)	5,185,899 (4,516,315, 5,779,750)	4,058,362 (3,519,385, 4,594,114)	2,156,849 (1,842,220, 2,534,981)	0.0 (0.0, 0.0)	28.9 (23.1, 37.7)	49.5 (40.6, 56.7)	53.9 (44.8, 60.7)	55.3 (47.3, 61.8)	52.5 (45.7, 58.5)	48.3 (41.9, 54.6)	39.5 (33.7, 46.4)
Pancreatitis	0 (0, 0)	7,941 (5,277, 13,260)	110,814 (76,011, 154,625)	219,700 (161,073, 287,633)	239,264 (172,474, 317,284)	195,109 (145,157, 259,280)	144,004 (105,491, 197,818)	112,758 (79,154, 162,905)	0.0 (0.0, 0.0)	11.1 (7.4, 18.5)	27.2 (18.6, 37.9)	32.1 (23.6, 42.1)	31.5 (22.7, 41.8)	28.2 (21.0, 37.4)	23.5 (17.2, 32.3)	18.8 (13.2, 27.1)
Injuries	2,962,160 (2,169,450, 4,874,306)	3,156,648 (2,459,336, 4,458,568)	13,167,738 (10,125,683, 17,003,119)	10,702,677 (8,472,033, 13,561,424)	8,484,415 (6,820,936, 10,705,939)	6,529,457 (5,295,709, 8,230,714)	4,249,513 (3,450,497, 5,431,095)	2,877,018 (2,309,468, 3,796,437)	4.9 (3.6, 8.1)	12.7 (9.9, 17.9)	21.8 (16.7, 28.1)	23.7 (18.8, 30.0)	24.8 (19.9, 31.3)	22.8 (18.5, 28.7)	19.0 (15.4, 24.3)	13.2 (10.6, 17.5)
Unintentional injuries	2,962,160 (2,169,450, 4,874,306)	2,361,307 (1,851,559, 3,434,686)	9,044,697 (7,027,113, 12,020,861)	7,445,716 (5,872,744, 9,690,826)	6,457,902 (5,120,428, 8,422,850)	5,220,993 (4,118,384, 6,741,559)	3,596,250 (2,844,773, 4,720,588)	2,568,403 (2,015,558, 3,449,989)	5.6 (4.1, 9.1)	15.6 (12.3, 22.7)	25.6 (19.9, 34.0)	26.5 (20.9, 34.5)	26.8 (21.2, 35.0)	24.0 (18.9, 31.0)	19.7 (15.6, 25.8)	13.4 (10.5, 18.0)

Cause of disease or injury	Alcohol-attributable DALYs lost								Population Attributable Fraction							
	0 to 14	15 to 19	20 to 29	30 to 39	40 to 49	50 to 59	60 to 69	≥70	0 to 14	15 to 19	20 to 29	30 to 39	40 to 49	50 to 59	60 to 69	≥70
Road injury	2,962,160 (2,169,450, 4,874,306)	1,586,210 (1,118,098, 2,487,277)	5,579,073 (3,971,083, 8,100,674)	4,030,342 (2,887,290, 5,795,446)	3,256,654 (2,332,968, 4,679,086)	2,409,537 (1,741,305, 3,511,358)	1,537,459 (1,117,165, 2,272,279)	879,758 (644,326, 1,331,790)	23.1 (16.9, 38.0)	20.3 (14.3, 31.8)	29.1 (20.7, 42.2)	30.0 (21.5, 43.1)	30.2 (21.6, 43.4)	28.0 (20.2, 40.8)	25.8 (18.8, 38.2)	22.4 (16.4, 33.9)
Poisonings	0 (0, 0)	33,394 (21,251, 54,353)	181,963 (116,149, 254,127)	141,495 (94,007, 193,706)	121,685 (80,535, 167,209)	82,108 (55,690, 111,211)	54,650 (36,744, 75,803)	26,028 (17,014, 38,158)	0.0 (0.0, 0.0)	10.2 (6.5, 16.6)	20.7 (13.2, 28.9)	22.9 (15.2, 31.4)	21.9 (14.5, 30.1)	19.5 (13.2, 26.4)	14.6 (9.8, 20.2)	9.8 (6.4, 14.4)
Falls	0 (0, 0)	143,834 (92,279, 217,295)	766,357 (489,118, 1,048,346)	951,913 (628,095, 1,284,331)	1,090,702 (721,716, 1,476,318)	1,132,104 (764,833, 1,524,864)	955,789 (656,641, 1,303,255)	979,163 (676,874, 1,384,573)	0.0 (0.0, 0.0)	11.9 (7.6, 18.0)	23.0 (14.7, 31.4)	24.6 (16.2, 33.2)	25.2 (16.7, 34.2)	21.8 (14.7, 29.4)	16.4 (11.3, 22.4)	10.7 (7.4, 15.2)
Fire, heat and hot substances	0 (0, 0)	54,613 (36,441, 85,632)	258,597 (174,512, 358,115)	266,664 (188,264, 356,501)	214,787 (156,025, 281,126)	180,946 (133,322, 234,861)	117,309 (85,865, 154,621)	73,836 (53,534, 101,210)	0.0 (0.0, 0.0)	7.6 (5.0, 11.8)	15.8 (10.7, 21.9)	18.4 (13.0, 24.6)	21.2 (15.4, 27.7)	20.0 (14.7, 25.9)	16.7 (12.2, 22.0)	11.6 (8.4, 15.9)
Drowning	0 (0, 0)	203,101 (116,531, 332,181)	580,148 (347,793, 819,587)	420,355 (276,776, 571,129)	291,059 (192,681, 394,330)	198,266 (131,398, 268,872)	125,659 (82,094, 175,477)	70,612 (45,025, 104,078)	0.0 (0.0, 0.0)	11.6 (6.7, 19.0)	23.3 (14.0, 33.0)	25.9 (17.0, 35.2)	26.0 (17.2, 35.2)	22.4 (14.8, 30.4)	17.6 (11.5, 24.6)	11.6 (7.4, 17.1)
Exposure to mechanical forces	0 (0, 0)	80,827 (47,202, 130,742)	435,686 (259,485, 615,796)	430,017 (269,802, 591,842)	400,590 (254,970, 548,324)	322,151 (206,297, 444,436)	201,627 (130,404, 280,721)	110,235 (73,716, 157,445)	0.0 (0.0, 0.0)	11.5 (6.7, 18.6)	23.4 (13.9, 33.1)	25.1 (15.8, 34.6)	26.0 (16.6, 35.6)	23.6 (15.1, 32.6)	19.8 (12.8, 27.5)	13.9 (9.3, 19.8)
Other unintentional injuries	0 (0, 0)	259,328 (158,224, 413,847)	1,242,873 (758,223, 1,754,153)	1,204,931 (776,684, 1,651,627)	1,082,425 (717,630, 1,479,803)	895,882 (608,488, 1,208,892)	603,755 (412,280, 829,317)	428,771 (286,543, 615,570)	0.0 (0.0, 0.0)	10.2 (6.2, 16.2)	21.0 (12.8, 29.6)	22.5 (14.5, 30.9)	23.0 (15.3, 31.5)	20.7 (14.0, 27.9)	16.5 (11.3, 22.7)	11.3 (7.5, 16.2)
Intentional injuries	0 (0, 0)	795,341 (318,446, 1,446,528)	4,123,041 (1,673,550, 6,469,147)	3,256,961 (1,524,116, 4,882,996)	2,026,513 (969,300, 3,011,524)	1,308,464 (676,049, 1,931,367)	653,263 (344,469, 983,676)	308,614 (168,631, 480,287)	0.0 (0.0, 0.0)	8.2 (3.3, 14.8)	16.4 (6.7, 25.8)	19.1 (8.9, 28.7)	20.0 (9.6, 29.7)	19.0 (9.8, 28.0)	16.0 (8.4, 24.1)	12.0 (6.5, 18.7)
Self-harm	0 (0, 0)	336,749 (165,197, 578,392)	1,981,393 (947,146, 2,998,888)	1,799,575 (952,161, 2,621,975)	1,258,661 (645,863, 1,839,977)	909,458 (484,000, 1,329,379)	486,055 (251,548, 734,894)	247,048 (129,306, 388,402)	0.0 (0.0, 0.0)	8.5 (4.2, 14.6)	18.4 (8.8, 27.8)	23.0 (12.2, 33.5)	23.7 (12.2, 34.6)	21.9 (11.6, 31.9)	17.5 (9.1, 26.5)	12.8 (6.7, 20.1)
Interpersonal violence	0 (0, 0)	458,592 (144,341, 873,710)	2,141,648 (694,773, 3,498,471)	1,457,386 (580,464, 2,277,244)	767,852 (327,991, 1,182,426)	399,006 (198,834, 604,737)	167,208 (91,724, 249,518)	61,566 (38,620, 91,584)	0.0 (0.0, 0.0)	10.9 (3.4, 20.9)	20.5 (6.6, 33.5)	22.1 (8.8, 34.5)	21.8 (9.3, 33.6)	20.2 (10.0, 30.6)	17.3 (9.5, 25.9)	12.0 (7.5, 17.9)

Table A17. Global alcohol-attributable premature disability adjusted life years (DALYs) lost by cause and sex in 2016 and 95% uncertainty intervals

Cause of disease or injury	Men		Women		Total	
	Alcohol-attributable burden	PAF (%)	Alcohol-attributable burden	PAF (%)	Alcohol-attributable burden	PAF (%)
**All Causes**	90,879,176 (76,378,685, 108,428,540)	7.8 (6.5, 9.3)	26,345,442 (23,075,646, 34,874,848)	2.7 (2.4, 3.6)	117,224,618 (102,052,811, 138,931,652)	5.5 (4.8, 6.5)
**Communicable, maternal, perinatal and nutritional conditions**	9,908,163 (3,936,122, 16,533,780)	2.7 (1.1, 4.6)	2,511,639 (1,118,853, 6,088,395)	0.7 (0.3, 1.8)	12,419,802 (6,266,466, 20,328,764)	1.8 (0.9, 2.9)
Tuberculosis	7,439,957 (1,552,128, 13,682,543)	25.4 (5.3, 46.6)	1,687,899 (278,291, 4,548,138)	10.4 (1.7, 28.0)	9,127,856 (3,017,647, 16,556,043)	20.0 (6.6, 36.3)
HIV AIDS	1,216,859 (719,517, 1,884,240)	3.6 (2.1, 5.6)	437,562 (258,615, 1,283,690)	1.7 (1.0, 5.0)	1,654,421 (1,133,333, 2,740,916)	2.8 (1.9, 4.6)
Lower respiratory infections	1,251,347 (498,721, 2,165,302)	2.1 (0.8, 3.7)	386,178 (186,302, 828,077)	0.7 (0.4, 1.6)	1,637,524 (901,009, 2,717,827)	1.5 (0.8, 2.4)
**Noncommunicable diseases**	41,375,937 (36,406,981, 45,512,375)	6.7 (5.9, 7.4)	14,176,272 (12,131,712, 18,135,966)	2.6 (2.3, 3.4)	55,552,208 (49,937,137, 61,950,937)	4.8 (4.3, 5.4)
Malignant neoplasms	6,993,259 (5,745,973, 8,288,043)	7.0 (5.7, 8.3)	2,404,760 (2,046,840, 3,019,572)	3.0 (2.5, 3.7)	9,398,019 (8,011,446, 11,028,749)	5.2 (4.4, 6.1)
Lip and oral cavity cancer	1,217,050 (967,441, 1,411,386)	38.1 (30.3, 44.1)	213,603 (154,922, 287,805)	16.3 (11.8, 22.0)	1,430,652 (1,148,754, 1,666,810)	31.7 (25.5, 37.0)
Other pharynx cancers	941,259 (765,106, 1,079,478)	40.8 (33.2, 46.8)	88,183 (65,105, 119,434)	16.6 (12.3, 22.5)	1,029,442 (842,803, 1,182,810)	36.3 (29.7, 41.7)
Oesophagus cancer	1,461,300 (1,201,954, 1,691,963)	24.7 (20.3, 28.6)	169,771 (127,064, 234,697)	8.8 (6.6, 12.1)	1,631,071 (1,353,846, 1,896,234)	20.8 (17.2, 24.2)
Colon and rectum cancers	1,198,119 (950,668, 1,449,184)	16.4 (13.0, 19.8)	266,463 (158,332, 410,296)	5.0 (3.0, 7.7)	1,464,582 (1,198,640, 1,767,212)	11.6 (9.5, 14.0)
Liver cancer	1,740,924 (838,042, 2,734,721)	12.4 (5.9, 19.4)	449,356 (234,378, 757,186)	10.1 (5.2, 16.9)	2,190,279 (1,260,119, 3,288,079)	11.8 (6.8, 17.7)
Breast cancer	0 (0, 0)	0.0 (0.0, 0.0)	1,194,219 (963,536, 1,534,095)	7.5 (6.0, 9.6)	1,194,219 (963,537, 1,534,112)	7.4 (6.0, 9.5)
Larynx cancer	434,608 (329,636, 542,094)	24.9 (18.9, 31.0)	23,165 (15,666, 34,632)	10.2 (6.9, 15.2)	457,773 (352,125, 567,744)	23.2 (17.8, 28.7)
Diabetes mellitus	461,038 (55,275, 1,063,911)	1.9 (0.2, 4.3)	-1,725,025 (-2,389,315, -916,666)	-7.5 (-10.5, -4.0)	-1,263,988 (-2,016,589, -209,011)	-2.7 (-4.2, -0.4)
Alcohol use disorders	13,197,017 (13,197,017, 13,197,017)	100.0 (100.0, 100.0)	4,652,271 (4,652,271, 4,652,271)	100.0 (100.0, 100.0)	17,849,288 (17,849,288, 17,849,288)	100.0 (100.0, 100.0)
Epilepsy	993,768 (740,562, 1,255,344)	13.0 (9.7, 16.4)	343,997 (263,170, 488,579)	5.5 (4.2, 7.9)	1,337,765 (1,067,640, 1,667,219)	9.7 (7.7, 12.0)
Cardiovascular diseases	4,702,283 (1,679,256, 7,113,844)	3.1 (1.1, 4.7)	3,292,363 (1,503,273, 6,530,505)	3.4 (1.5, 6.7)	7,994,646 (4,499,888, 11,982,552)	3.2 (1.8, 4.8)
Hypertensive heart disease	763,227 (576,060, 932,902)	14.4 (10.9, 17.6)	172,231 (81,555, 377,337)	3.3 (1.6, 7.3)	935,458 (731,067, 1,209,795)	8.9 (7.0, 11.6)
Ischaemic heart disease	279,689 (-2,552,552, 2,095,792)	0.3 (-3.2, 2.6)	1,348,816 (-437,955, 3,590,607)	3.3 (-1.1, 8.7)	1,628,505 (-1,609,157, 4,430,143)	1.3 (-1.3, 3.6)
Ischaemic stroke	-185,588 (-543,668, 326,141)	-1.2 (-3.4, 2.1)	-529,570 (-921,027, 231,946)	-4.1 (-7.2, 1.8)	-715,158 (-1,233,100, 331,769)	-2.5 (-4.3, 1.2)
Haemorrhagic stroke	3,190,101 (2,114,332, 4,360,210)	10.7 (7.1, 14.6)	2,189,192 (1,304,271, 3,660,542)	9.7 (5.8, 16.2)	5,379,292 (3,967,530, 7,325,634)	10.3 (7.6, 14.0)
Cardiomyopathy, myocarditis, endocarditis	654,855 (645,483, 775,017)	11.0 (10.8, 13.0)	111,694 (111,676, 112,299)	3.3 (3.3, 3.3)	766,549 (757,234, 886,879)	8.2 (8.1, 9.5)
Digestive diseases	15,028,572 (12,339,370, 17,192,419)	32.5 (26.6, 37.1)	5,207,906 (4,451,236, 6,060,981)	19.3 (16.5, 22.4)	20,236,477 (17,364,266, 22,756,595)	27.6 (23.7, 31.0)
Cirrhosis of the liver	14,228,062 (11,612,940, 16,361,339)	50.8 (41.5, 58.5)	5,091,583 (4,339,647, 5,887,297)	43.0 (36.7, 49.7)	19,319,645 (16,494,988, 21,762,330)	48.5 (41.4, 54.6)
Pancreatitis	800,509 (544,677, 1,048,757)	35.3 (24.0, 46.3)	116,323 (41,752, 280,670)	11.4 (4.1, 27.5)	916,832 (665,484, 1,229,901)	27.9 (20.3, 37.4)
**Injuries**	39,595,076 (29,715,265, 52,696,142)	21.1 (15.8, 28.1)	9,657,532 (7,710,848, 13,435,618)	11.0 (8.8, 15.3)	49,252,608 (38,793,643, 64,265,164)	17.9 (14.1, 23.3)
Unintentional injuries	29,322,224 (21,730,237, 40,112,045)	22.2 (16.5, 30.4)	7,766,802 (6,267,656, 10,982,985)	12.1 (9.8, 17.1)	37,089,026 (29,004,452, 49,905,675)	18.9 (14.8, 25.5)
Road injury	16,725,842 (11,466,395, 24,986,965)	28.7 (19.7, 42.9)	4,635,593 (3,624,381, 6,898,925)	22.7 (17.8, 33.9)	21,361,435 (15,337,359, 31,720,427)	27.2 (19.5, 40.4)
Poisonings	465,261 (262,833, 682,197)	13.4 (7.6, 19.7)	150,034 (82,932, 247,445)	5.9 (3.3, 9.8)	615,296 (404,376, 856,410)	10.2 (6.7, 14.3)
Falls	3,929,816 (2,291,401, 5,648,237)	21.6 (12.6, 31.0)	1,110,883 (712,384, 1,661,618)	10.3 (6.6, 15.4)	5,040,699 (3,352,682, 6,854,409)	17.4 (11.5, 23.6)
Fire, heat and hot substances	737,201 (453,143, 1,040,360)	16.0 (9.8, 22.5)	355,716 (212,439, 566,879)	6.6 (4.0, 10.6)	1,092,916 (774,430, 1,470,856)	11.0 (7.8, 14.7)
Drowning	1,570,702 (909,436, 2,292,029)	11.9 (6.9, 17.3)	247,886 (140,758, 404,429)	3.9 (2.2, 6.4)	1,818,588 (1,147,273, 2,561,574)	9.3 (5.9, 13.1)
Exposure to mechanical forces	1,597,466 (904,099, 2,317,101)	18.5 (10.5, 26.8)	273,431 (165,597, 423,741)	7.2 (4.4, 11.2)	1,870,898 (1,168,161, 2,611,861)	15.1 (9.4, 21.0)
Other unintentional injuries	4,295,935 (2,472,890, 6,252,401)	17.1 (9.8, 24.8)	993,259 (611,036, 1,558,470)	6.7 (4.1, 10.5)	5,289,194 (3,431,528, 7,337,638)	13.2 (8.6, 18.3)
Intentional injuries	10,272,852 (3,523,988, 16,591,660)	18.4 (6.3, 29.6)	1,890,730 (798,601, 3,339,406)	8.0 (3.4, 14.1)	12,163,582 (5,505,931, 18,725,238)	15.3 (6.9, 23.5)
Self-harm	5,495,741 (2,187,462, 8,563,523)	24.3 (9.7, 37.9)	1,276,150 (497,970, 2,289,160)	9.8 (3.8, 17.6)	6,771,890 (3,445,915, 10,103,506)	19.0 (9.7, 28.4)
Interpersonal violence	4,777,111 (1,316,040, 8,030,143)	19.9 (5.5, 33.4)	614,581 (292,908, 1,055,718)	9.2 (4.4, 15.7)	5,391,692 (2,038,128, 8,686,106)	17.5 (6.6, 28.3)

PAF: Population Attributable Fraction

References

1. Shield, K.; Manthey, J.; Rylett, M.; Probst, C.; Wettlaufer, A.; Parry, C.D.; Rehm, J. National, regional, and global burdens of disease from 2000 to 2016 attributable to alcohol use: a comparative risk assessment study. *Lancet Public Health* **2020**, *5*, e51-e61.
2. World Health Organization. *WHO Road traffic death database*; World Health Organization: Geneva, Switzerland, 2018.
3. Manthey, J.; Shield, K.D.; Rylett, M.; Hasan, O.S.; Probst, C.; Rehm, J. Global alcohol exposure between 1990 and 2017 and forecasts until 2030: a modelling study. *Lancet* **2019**, *393*, 22-28.
4. Gmel, G.; Rehm, J. Measuring alcohol consumption. *Contemporary Drug Problems* **2004**, *31*, 467.
5. King, A.C. Enhancing the self-report of alcohol consumption in the community: two questionnaire formats. *Am. J. Public Health* **1994**, *84*, 294-296.
6. Rehm, J. Measuring alcohol consumption: how about adopting usual epidemiological standards? **1998**, *93*, 970–972.
7. Feunekes, G.I.; van't Veer, P.; van Staveren, W.A.; Kok, F.J. Alcohol intake assessment: the sober facts. *Am. J. Epidemiol.* **1999**, *150*, 105-112.
8. Shield, K.D.; Rehm, J. Difficulties with telephone-based surveys on alcohol consumption in high-income countries: the Canadian example. *Int. J. Methods Psychiatr. Res.* **2012**, *21*, 17-28.
9. Rehm, J.; Kehoe, T.; Gmel, G.; Stinson, F.; Grant, B.; Gmel, G. Statistical modeling of volume of alcohol exposure for epidemiological studies of population health: the US example. *Popul Health Metr* **2010**, *8*.
10. Kehoe, T.; Gmel, G.; Shield, K.D.; Gmel, G.; Rehm, J. Determining the best population-level alcohol consumption model and its impact on estimates of alcohol-attributable harms. *Popul Health Metr* **2012**, *10*.
11. Imtiaz, S.; Shield, K.D.; Roerecke, M.; Samokhvalov, A.V.; Lönnroth, K.; Rehm, J. Alcohol consumption as a risk factor for tuberculosis: meta-analyses and burden of disease. *Eur. Respir. J.* **2017**, *50*, 1700216.
12. Rehm, J.; Samokhvalov, A.V.; Neuman, M.G.; Room, R.; Parry, C.; Lönnroth, K.; Patra, J.; Poznyak, V.; Popova, S. The association between alcohol use, alcohol use disorders and tuberculosis (TB). A systematic review. *BMC Public Health* **2009**, *9*.
13. Rehm, J.; Probst, C.; Shield, K.; Shuper, P. Does alcohol use have a causal effect on HIV incidence and disease progression? A review of the literature and a modeling strategy for quantifying the effect. *Popul Health Metr* **2017**, *15*.
14. Scott-Sheldon, L.A.; Carey, K.B.; Cunningham, K.; Johnson, B.T.; Carey, M.P.; Team, M.R. Alcohol use predicts sexual decision-making: a systematic review and meta-analysis of the experimental literature. *AIDS Behav.* **2016**, *20*, 19-39.
15. Samokhvalov, A.; Irving, H.; Rehm, J. Alcohol consumption as a risk factor for pneumonia: a systematic review and meta-analysis. *Epidemiol. Infect.* **2010**, *138*, 1789-1795.
16. Traphagen, N.; Tian, Z.; Allen-Gipson, D. Chronic ethanol exposure: pathogenesis of pulmonary disease and dysfunction. *Biomolecules* **2015**, *5*, 2840-2853.

17. Simet, S.M.; Sisson, J.H. Alcohol's effects on lung health and immunity. *Alcohol Res* **2015**, *37*, 199.
18. Bagnardi, V.; Rota, M.; Botteri, E.; Tramacere, I.; Islami, F.; Fedirko, V.; Scotti, L.; Jenab, M.; Turati, F.; Pasquali, E.; et al. Alcohol consumption and site-specific cancer risk: a comprehensive dose-response meta-analysis. *Br. J. Cancer* **2015**, *112*, 580-593, doi:10.1038/bjc.2014.579.
19. Marron, M.; Boffetta, P.; Zhang, Z.-F.; Zaridze, D.; Wünsch-Filho, V.; Winn, D.M.; Wei, Q.; Talamini, R.; Szeszenia-Dabrowska, N.; Sturgis, E.M. Cessation of alcohol drinking, tobacco smoking and the reversal of head and neck cancer risk. *Int. J. Epidemiol.* **2010**, *39*, 182-196.
20. International Agency for Research on Cancer. *IARC monographs on the evaluation of carcinogenic risks to humans: volume 100E - personal habits and indoor combustions*; International Agency for Research on Cancer: Lyon, France, 2009.
21. International Agency for Research on Cancer. *IARC monographs on the evaluation of carcinogenic risks to humans: volume 96 - alcohol consumption and ethyl carbamate*; International Agency for Research on Cancer: Lyon, France, 2007.
22. Schütze, M.; Boeing, H.; Pischon, T.; Rehm, J.; Kehoe, T.; Gmel, G.; Olsen, A.; Tjønneland, A.M.; Dahm, C.C.; Overvad, K. Alcohol attributable burden of incidence of cancer in eight European countries based on results from prospective cohort study. *BMJ* **2011**, *342*, d1584.
23. Turati, F.; Galeone, C.; Rota, M.; Pelucchi, C.; Negri, E.; Bagnardi, V.; Corrao, G.; Boffetta, P.; La Vecchia, C. Alcohol and liver cancer: a systematic review and meta-analysis of prospective studies. *Ann. Oncol.* **2014**, *25*, 1526-1535.
24. World Cancer Research Fund International; American Institute for Cancer Research. *Continuous Update Project report: food, nutrition, physical activity, and the prevention of liver cancer*; World Cancer Research Fund International: London, UK, 2015.
25. Knott, C.; Bell, S.; Britton, A. Alcohol consumption and the risk of type 2 diabetes: a systematic review and dose-response meta-analysis of more than 1.9 million individuals from 38 observational studies. *Diabetes Care* **2015**, *38*, 1804-1812.
26. Rehm, J.; Baliunas, D.; Borges, G.L.; Graham, K.; Irving, H.; Kehoe, T.; Parry, C.D.; Patra, J.; Popova, S.; Poznyak, V.; et al. The relation between different dimensions of alcohol consumption and burden of disease: an overview. *Addiction* **2010**, *105*, 817-843, doi:10.1111/j.1360-0443.2010.02899.x.
27. Samokhvalov, A.V.; Irving, H.; Mohapatra, S.; Rehm, J. Alcohol consumption, unprovoked seizures, and epilepsy: A systematic review and meta-analysis. *Epilepsia* **2010**, *51*, 1177-1184.
28. Bartolomei, F.; Suchet, L.; Barrie, M.; Gastaut, J.-L. Alcoholic epilepsy: a unified and dynamic classification. *Eur. Neurol.* **1997**, *37*, 13-17.
29. Barclay, G.; Barbour, J.; Stewart, S.; Day, C.; Gilvarry, E. Adverse physical effects of alcohol misuse. *Adv Psychiatr Treat* **2008**, *14*, 139-151.
30. Leach, J.P.; Mohanraj, R.; Borland, W. Alcohol and drugs in epilepsy: pathophysiology, presentation, possibilities, and prevention. *Epilepsia* **2012**, *53*, 48-57.
31. World Health Organization. *Global status report on alcohol and health, 2018*; World Health Organization: Geneva, Switzerland, 2018.

32. Puddey, I.B.; Rakic, V.; Dimmitt, S.; Beilin, L. Influence of pattern of drinking on cardiovascular disease and cardiovascular risk factors-a review. *Addiction* **1999**, *94*, 649-663.
33. O'Keefe, J.H.; Bhatti, S.K.; Bajwa, A.; DiNicolantonio, J.J.; Lavie, C.J. Alcohol and cardiovascular health: the dose makes the poison… or the remedy. *Mayo Clin. Proc.* **2014**, *89*, 382-393.
34. Rehm, J.; Shield, K.D.; Roerecke, M.; Gmel, G. Modelling the impact of alcohol consumption on cardiovascular disease mortality for comparative risk assessments: an overview *BMC Public Health* **2016**, *16*, 363.
35. Roerecke, M.; Rehm, J. The cardioprotective association of average alcohol consumption and ischaemic heart disease: a systematic review and meta-analysis. *Addiction* **2012**, *107*, 1246-1260.
36. Roerecke, M.; Rehm, J. Ischemic heart disease mortality and morbidity in former drinkers: a meta-analysis. *Am J Epidemiol* **2011**, *173*, 245-258, doi:10.1093/aje/kwq364.
37. Roerecke, M.; Rehm, J. Alcohol consumption, drinking patterns, and ischemic heart disease: a narrative review of meta-analyses and a systematic review and meta-analysis of the impact of heavy drinking occasions on risk for moderate drinkers. *BMC Med* **2014**, *12*, 182, doi:10.1186/s12916-014-0182-6.
38. Mukamal, K.J.; Rimm, E.B. Alcohol's effects on the risk for coronary heart disease. *Alcohol Res Health* **2001**, *25*, 255-261.
39. Collins, M.A.; Neafsey, E.J.; Mukamal, K.J.; Gray, M.O.; Parks, D.A.; Das, D.K.; Korthuis, R.J. Alcohol in moderation, cardioprotection, and neuroprotection: epidemiological considerations and mechanistic studies. *Alcohol Clin Exp Res* **2009**, *33*, 206-219.
40. Patra, J.; Taylor, B.; Irving, H.; Roerecke, M.; Baliunas, D.; Mohapatra, S.; Rehm, J. Alcohol consumption and the risk of morbidity and mortality from different stroke types - a systematic review and meta-analysis. *BMC Public Health* **2010**, *10*, 258.
41. Puddey, I.B.; Rakic, V.; Dimmitt, S.B.; Beilin, L.J. Influence of pattern of drinking on cardiovascular disease and cardiovascular risk factors - a review. *Addiction* **1999**, *94*, 649-663.
42. Mazzaglia, G.; Britton, R.; Altmann, D.R.; Chenet, L. Exploring the relationship between alcohol consumption and non-fatal or fatal stroke: a systematic review. *Addiction* **2001**, *96*, 1743-1756.
43. Larsson, S.C.; Wallin, A.; Wolk, A.; Markus, H.S. Differing association of alcohol consumption with different stroke types: a systematic review and meta-analysis. *BMC Med* **2016**, *14*, 178.
44. Gao, B.; Bataller, R. Alcoholic liver disease: pathogenesis and new therapeutic targets. *Gastroenterology* **2011**, *141*, 1572-1585.
45. Braganza, J.M.; Lee, S.H.; McCloy, R.F.; McMahon, M.J. Chronic pancreatitis. *Lancet* **2011**, *377*, 1184-1197.
46. Yadav, D.; Lowenfels, A.B. The epidemiology of pancreatitis and pancreatic cancer. *Gastroenterology* **2013**, *144*, 1252-1261.
47. Lankisch, P.; Apte, M.; Banks, P. Acute pancreatitis. *Lancet* **2015**, *386*, 2058-2058.
48. Majumder, S.; Chari, S.T. Chronic pancreatitis. *Lancet* **2016**, *387*, 1957-1966.

49. Samokhvalov, A.V.; Rehm, J.; Roerecke, M. Alcohol consumption as a risk factor for acute and chronic pancreatitis: a systematic review and a series of meta-analyses. *EBioMedicine* **2015**, *2*, 1996-2002.
50. World Health Organization. *Alcohol and injuries: emergency department studies in an international perspective*; World Health Organization Geneva, Switzerland, 2009.
